# Causal Relationship Between Various Vitamins and Different Diabetic Complications: A Mendelian Randomization Study

**DOI:** 10.1002/fsn3.70536

**Published:** 2025-07-07

**Authors:** Sijia Cai, Weitao Man, Wenqing Liu, Bowu Li, Zhongchen He, Guman Duan

**Affiliations:** ^1^ Department of Endocrinology Beijing Hepingli Hospital Beijing China; ^2^ Department of Neurosurgery, Beijing Tsinghua Changgung Hospital, School of Clinical Medicine, Tsinghua Medicine Tsinghua University Beijing China; ^3^ Orthopedics & Sports Medicine Center, Beijing Tsinghua Changgung Hospital, School of Clinical Medicine, Tsinghua Medicine Tsinghua University Beijing China

**Keywords:** diabetic complication, inverse‐variance weighted, mendelian randomization analysis, vitamins

## Abstract

Epidemiological evidence regarding the association between vitamins and diabetic complications remains inconsistent. This study aims to explore the potential causal relationships between diabetic complications and circulating vitamins in diabetics. We selected vitamin A (VitA) genetic variants (*n* = 5,006), vitamin B_6_ (VitB_6_) genetic variants (*n* = 64,974), vitamin C (VitC) genetic variants (*n* = 52,018) and vitamin D (VitD) genetic variants (*n* = 441,291) as exposures of interest from large‐scale Genome‐Wide Association Studies (GWAS) databases. Then we performed two‐sample Mendelian randomization (MR) analyses to evaluate the causal association of plasma vitamin levels with diabetic complications, which included maculopathy, ketoacidosis, hypoglycemia, neuropathy, nephropathy and retinopathy. Multiple methods were performed and used including the inverse‐variance weighted (IVW), the weighted median, MR‐Egger and MR‐PRESSO regression. Heterogeneity and sensitivity analyses were conducted. The results of the IVW method revealed that the level of VitB_6_ were associated with diabetic hypoglycemia with (OR: 8.54; 95% CI: 1.77 to 41.2; *p*: 0.01). No association was detected between other vitamins (VitA, VitC or VitD) and diabetic complications (maculopathy, ketoacidosis, hypoglycemia, neuropathy, nephropathy or retinopathy). After MR‐PRESSO analysis, there was no causal relationship detected between VitD and diabetic hypoglycemia (OR: 0.867, CI: 0.64 to 1.18, *p*: 0.37), diabetic ketosis (OR: 0.763515, CI: 0.58 to 1.00, *p*: 0.055), diabetic maculopathy (OR: 0.72, CI: 0.48 to 1.07, *p*: 0.105). This analysis provided genetic evidence that the level of VitB_6_ may be the risk factor for diabetic hypoglycemia. VitA, VitC, or VitD were not associated with various diabetic complications. Monitoring for excess VitB_6_ and suitable supplements might be important, and other vitamins may have limited effects in complications prevention, and further investigations were needed to unveil the mechanisms.

## Graphical abstract

The potential causal relationships between six diabetic complications and four circulating vitamins in diabetics were explored through two‐sample Mendelian randomization analyses.

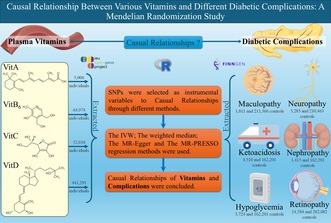

## 1 Introduction

Diabetes mellitus (DM) is a chronic metabolic disease characterized by elevated blood glucose levels and inadequate insulin secretion (Ahmad et al. 2022). According to the International Diabetes Federation (IDF), DM is estimated to be responsible for 6.7 million deaths among adults under 70 years of age (Sun, Saeedi, et al. 2022). Various diabetic complications including diabetic maculopathy, diabetic ketoacidosis, diabetic hypoglycemia, diabetic neuropathy, diabetic nephropathy and diabetic retinopathy are all threatening the aging populations. As these multiple serious complications, it is important to investigate the monitor, prevention and treatment about these complications.

Vitamins (Vit) play an important role in the process of human growth, metabolism and development, maintaining normal physiological functions. Vitamin D (VitD) deficiency is associated with high burden (Erkus et al. 2018) of inflammation and correlated with poor glucose control in diabetic subjects (Erkus et al. 2019). In addition, vitamin C (VitC) levels are linked to the amelioration of the inflammatory burden in human (Li, Liu, et al. 2021). Another vitamin, vitamin A (VitA) have proved to reduces the inflammation during infections (Gholizadeh et al. 2022). Moreover, vitamin B_6_ (VitB_6_) have proved to alleviates inflammation (Mikkelsen et al. 2023). On the other hand, DM and its complications such as diabetic kidney disease (Aktas et al. 2023, 2024), diabetic retinopathy (Aktas 2023), and diabetic neuropathy (Aktas 2024) are characterized with high burden of chronic inflammation. Not only DM but also related conditions such as prediabetes (Balci et al. 2024), obesity (Duman et al. 2025), metabolic syndrome (Basaran and Aktas 2025), and fatty liver disease (Kosekli and Aktas 2025) are associated with chronic inflammation. Hence, studying the association between Vit levels and DM and its complications is reasonable.

Multiple studies have demonstrated robust and consistent associations between diabetes or complications with Vit (Christou et al. 2012; Erikstrup et al. 2009; Suh et al. 2010). Firstly, VitA were significantly lower in patients with T2DM than in healthy controls (Taneera et al. 2021), and has been reported plays an important role in islet regulation and pancreatic development (Rhee and Plutzky 2012). Adequate intake of VitA also proved to help protecting against diabetes (Su et al. 2022). In addition, a meta‐analysis showed that VitD reduced risk for diabetes by 15% (Pittas et al. 2023). Moreover, higher concentrations of pro‐vitamin A carotenoids were also associated with diabetic retinopathy (Brazionis et al. 2009). It has been also reported that the antioxidant Vit in T2DM patients was lower that healthy controls, which may causing the occurrence of vascular complications (Onyesom et al. 2011).

Although interactions between Vit and DM were deemed through previous studies, no studies were performed to conclude these relationships in multiple Vit or in large populations. The Mendelian randomization (MR) study is a genetic epidemiological approach to estimate the causal relationships between exposure and risk of disease which use genetic variants that are strongly associated with exposure factors as instrumental variables (IVs) for causal inference at the genetic level (Smith and Ebrahim 2003). Through this type of study, more convincing evidence can be provided by minimizing the potential bias arising from potential confounders, since genetic variants are randomly assigned.

In this study, we applied a MR approach to estimate the putative causal relationships between Vit and risk of diabetic complications, including diabetic nephropathy, diabetic neuropathy, diabetic retinopathy, diabetic maculopathy, diabetic ketoacidosis and diabetic hypoglycemia, to understand whether the circulating Vit level and diabetic complications interact through large populations based on MR analysis.

## 2 Materials and Methods

### 2.1 Study Design

**FIGURE 1:**
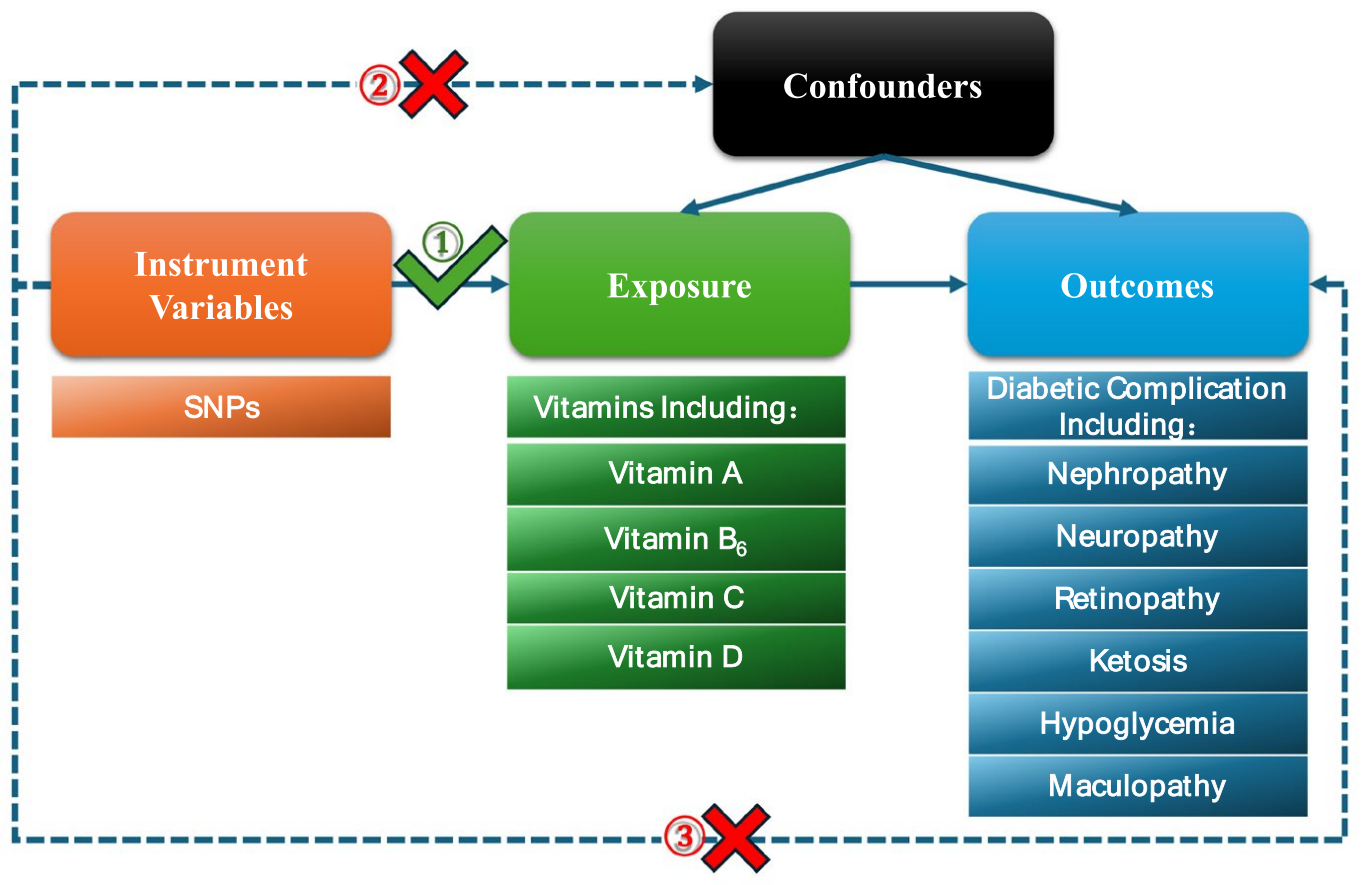
Flow chart of the Mendelian randomization (MR) study and three key assumptions. Firstly, the selected single nucleotide polymorphisms (SNPs) are strongly associated with exposure; vitamins are used as exposure factors in this article. Secondly, there is no association between the SNPs and confounders. Finally, the SNPs can only impact outcomes (diabetic complications) through vitamins directly.

This study followed the Strengthening the Reporting of Observational Studies in Epidemiology Using Mendelian Randomization (STROBE‐MR) (Skrivankova et al. 2021), and detailed information were listed in Appendix S11. We conducted a two‐sample MR to explore the association between genetic instrumental variables and outcomes. There are three assumptions that must be satisfied for IVs, including the relevance assumption, independence assumption, and exclusion restriction assumption. The genetic instrumental variables used in this study were conformed with three principles: (1) the single nucleotide polymorphisms (SNPs) are selected strongly associated with exposure and the vitamins are used as exposure factors in this article; (2) There is no association between the SNPs and confounders; (3) the SNPs can only impact various diabetic complications as an outcome through vitamins directly (Figure 1).

### 2.2 Genetic Instrumental Variables for Vitamin

Genetic instruments were identified using results from the available meta‐analysis of Genome‐Wide Association Studies (GWASs). The GWASs were restricted to individuals of European descent to limit bias from population stratification. Data for VitA, VitB_6_, and VitD were obtained from the GWASs database (Collins 2012). The SNPs associated with VitC were selected from a genome‐wide association study (Zheng et al. 2021), which included four studies of European ancestry (European Prospective Investigation into Cancer and Nutrition–Cardiovascular Disease study (EPIC‐CVD, *n* = 7,650) (Danesh et al. 2007), Fenland study (*n* = 10,771) (Ashor et al. 2017), EPIC Norfolk study (*n* = 16,756) (Day et al. 1999), and EPIC‐InterAct study (*n* = 16,841) (Langenberg et al. 2011)). In EPIC‐InterAct and EPIC‐CVD study, the overlapping individuals were excluded. The sample size of the retinol was 5006 individuals, the VitB_6_ was from 64,974 individuals, the VitC was from 52,018 individuals, and the VitD was from 441,291 individuals.

### 2.3 Genetic Association Datasets for Diabetic Complications

The Summary data from GWAS is for outcome data extraction and was available from the FinnGen consortium. The FinnGen is a large public‐private partnership aiming to collect and analyze genome and health data from 500,000 Finnish biobank participants (Kurki et al. 2023). Many controls and diabetic complications was included in the analysis in these studies, and six diabetic complications were selected as outcomes, including diabetic nephropathy (3,283 and 210,463 controls), diabetic neuropathy (1,415 and 162,201 controls), diabetic retinopathy (14,584 and 202,082 controls), diabetic maculopathy (1,811 and 211,566 controls), diabetic ketoacidosis (4,510 and 162,201 controls) and diabetic hypoglycemia (3,724 and 162,201 controls). The details information for characteristics of diabetic complications are summarized in Table 1.

**TABLE 1:** GWAS information for characteristics of diabetic complications.

Diabetic outcome	Ancestry	Cases/Controls	Number of SNPs	Build	GWAS ID
Nephropathy	European	3,283/210,463	16,380,453	HG19/GRCh37	finn‐b‐DM_NEPHROPATHY
Neuropathy	European	1,415/162,201	16,380,195	HG19/GRCh37	finn‐b‐DM_NEUROPATHY
Retinopathy	European	14,584/202,082	16,380,459	HG19/GRCh37	finn‐b‐DM_RETINOPATHY
Ketosis	European	4,510/162,201	16,380,176	HG19/GRCh37	finn‐b‐DM_KETOACIDOSIS
Hypoglycemia	European	3,724/162,201	16,380,203	HG19/GRCh37	finn‐b‐DM_HYPOGLYC
Maculopathy	European	1,811/211,566	16,380,450	HG19/GRCh37	finn‐b‐DM_MACULOPATHY

### 2.4 Selection of Genetic Instrumental Variables

The SNPs reached a genome‐wide significance level (*p* < 5 × 10^−8^) were selected as instrumental variables. To ensure the independence of genetic variants, the linkage imbalance was removed. These SNPs were further pruned with the stringent pairwise *r* ^2^ > 0.001 and distance threshold = 10,000 kb. As SNPs for incompatible alleles (rs7740812), it was correlated (*r* ^2^ < 0.01) in linkage disequilibrium (LD) analysis, the remaining 10 independent SNPs were included to establish the genetic IVs for VitC (*p* < 5 × 10^−8^). The SNPs for VitD, after removing the SNPs for being palindromic with intermediate allele frequencies (rs136224, rs2286779, rs2470937, rs2618487, rs4484519, rs589030, rs61826000 and rs7660883), 160 SNPs were included to establish the genetic IVs for VitD levels (*p* < 5 × 10^−8^). After selecting the IVs, 2 SNPs for VitA, 1 for VitB_6_, 10 for VitC, and 166 for VitD were included in the primary analysis. The information on characteristics of vitamins was summarized in Table 2. The *F*‐statistic was used to reflect the strength of association between SNP and exposure factors, when *F* > 10, it can be considered that there is no weak genetic tool bias between SNPs and exposure factors, the *F* statistics was calculated using the following formula (Burgess and Thompson 2011):

$$F=\frac{R^{2}\left(N-k-1\right)}{\left(1-R^{2}\right)\times k}$$

$$\begin{array}{c} R^{2}=2\times \mathrm{EAF}\times \left(1-\mathrm{EAF}\right)\times {\text{Beta}}^{2}/\\ \left[\left(2\times \mathrm{EAF}\times \left(1-\mathrm{EAF}\right)\times {\text{Beta}}^{2}+2\times \mathrm{EAF}\times \left(1-\mathrm{EAF}\right)\times N\times {\mathrm{SE}}^{2}\right)\right] \end{array}$$

where *R* ^2^ is the proportion of variance explained in the instrument, *N* is the sample size of the GWAS, and *k* is the number of IVs included in the instrument (Brion et al. 2013). More detailed information about SNPs can be found in Appendix S1.

**TABLE 2:** GWAS information for characteristics of vitamins.

Exposure	Data sources	Ancestry	Participants	SNPs (*n*)	*p*	GWAS ID
Vitamin A	PMID 21878437	European	5,006	2	5 × 10^−8^	—
Vitamin B_6_	UK biobank	European	64,979	1	5 × 10^−8^	ukb‐b‐7864
Vitamin C	EPIC‐CVD study	European	52,018	10	5 × 10^−8^	—
	Fenland study					
	EPIC Norfolk study					
	EPIC‐InterAct study					
Vitamin D	IEU	European	441,291	166	5 × 10^−8^	ieu‐b‐4808

### 2.5 Statistical Analysis

In this study, we used different statistical methods including the inverse‐variance weighted method (IVW) (Lee et al. 2016), MR‐Egger, Weighted Median Estimator (WME), and weighted mode (Hartwig et al. 2017). The IVW method was deemed as the primary MR approach to assume the absence of invalid genetic instruments, and the sensitivity analyses were performed to test the reliability of the MR causal impact. Cochran's *Q* method (Neupane et al. 2012) was used to test the heterogeneity of IVW method, and significant significance was concluded when the Cochran's *Q* is lower than 0.05, and there is potential heterogeneity among IVs. A random effects model was applied further other than the fixed effects model.

The MR‐Egger analysis method was used to test the pleiotropy of MR (Verbanck et al. 2018). It was considered statistically significant when *p* < 0.05, and the horizontal pleiotropy would lead to bias in the results. To test whether the results of MR were caused by the disproportionate influence of a single SNP, the leave‐one‐out analysis method was used for sensitivity analysis in this study, indicating that the most causal association signals were driven by any single SNP. The SNPs included in the study were excluded one by one at a time, and the remaining IVs were analyzed by IVW to test the stability of the results, to identify the SNP that is primarily affected by pleiotropy. The MR‐pleiotropy residual sum and outlier (MR‐PRESSO) (Burgess and Thompson 2017) was performed to remove the outliers that could affect the causal effect evaluation. After removing the outliers, we repeated Cochran's *Q* test, MR‐Egger intercept, and MR analysis until there was no indication of pleiotropy. The *p*‐value < 0.05 was regarded as suggestive evidence for a possible causal relationship. Results were presented as the odds ratio (OR) and 95% confidence interval (CI). The two‐sample MR (version 0.5.9) and MR‐PRESSO pack in the R software (version 4.3.1) were used for the two‐sample MR analysis.

## 3 Results

After selecting genetic instrumental variables, all the *F*‐values of the instrumental variables were all greater than 10, indicating robustness against weak instrumental variables (Appendix S1).

**FIGURE 2:**
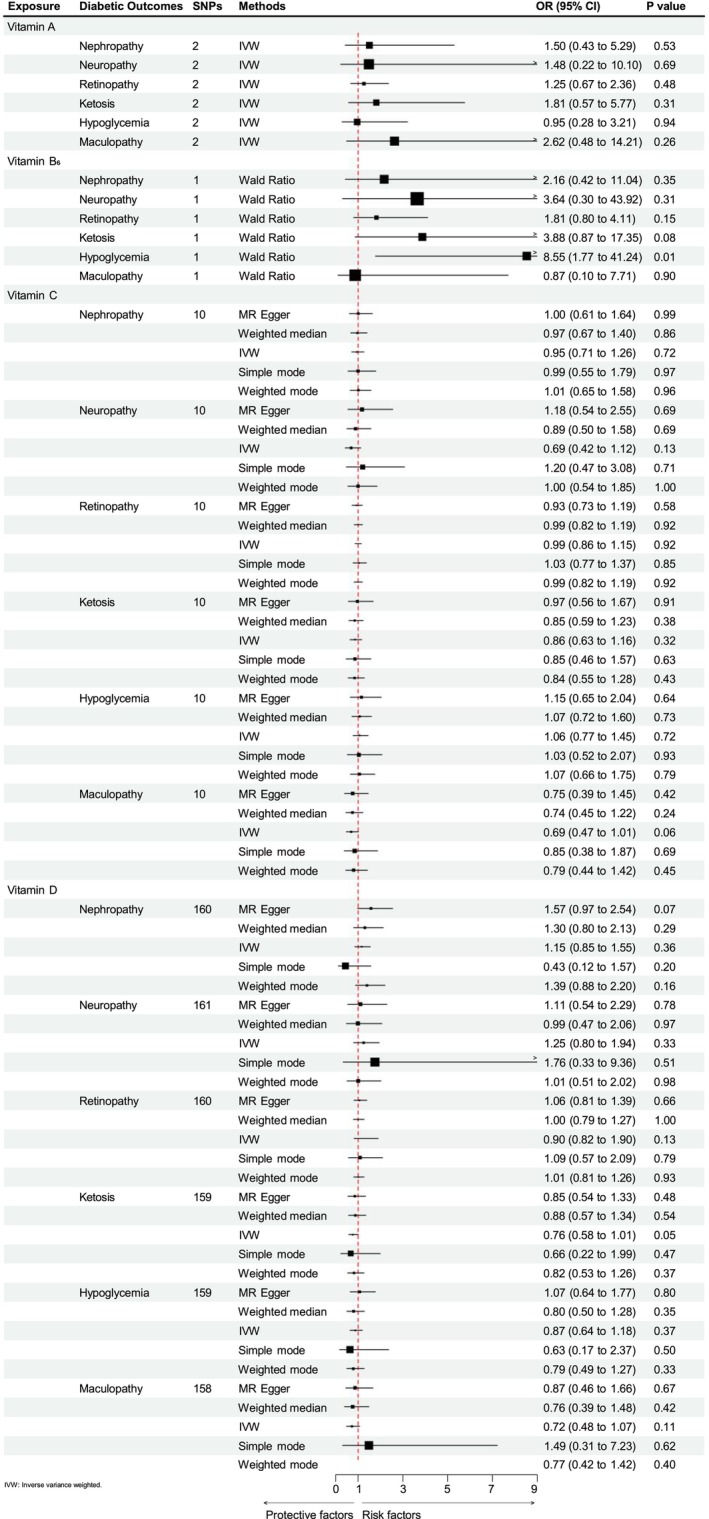
The forest plots and the results of the MR results of various vitamins and different diabetic complications. The width of the horizontal line represents the 95% CI of the individual methods, and the square represents the pooled OR and the size represents the standard error of each pooling (CI, confidence interval; OR, odds ratio).

The plasma retinol is the main constitute of VitA, and the retinol was used as an exposure factor in this MR analysis. As a higher plasma retinol level did not detected a statistically significant causal effect with any of the diabetic complications through genetically prediction, such as diabetic nephropathy (OR = 1.50, CI: 0.43 to 5.29, *p* = 0.53), diabetic neuropathy (OR = 1.48, CI: 0.22 to 10.10, *p* = 0.69), diabetic retinopathy (OR = 1.25, CI: 0.67 to 2.36, *p* = 0.48), diabetic ketosis (OR = 1.81, CI: 0.57 to 5.77, *p* = 0.31), diabetic hypoglycemia (OR = 0.95, CI: 0.28 to 3.21, *p* = 0.94) and diabetic maculopathy (OR = 2.62, CI: 0.48 to 14.2, *p* = 0.26). There were little data to determine heterogeneity and gene polymorphism (Figure 2).

VitB_6_ is associated with an increased risk of diabetic hypoglycemia. For VitB_6_, which had only one SNP as an instrumental variable, we analyzed its association with frailty using the Wald ratio method. Genetically predicted plasma VitB_6_ levels were associated with diabetic hypoglycemia (OR = 8.54, CI: 1.77 to 41.2, *p* = 0.0075). The VitB_6_ levels were not significantly associated with other diabetic complications, such as diabetic nephropathy (OR = 2.16, CI: 0.42 to 11.04, *p* = 0.35), diabetic neuropathy (OR = 3.64, CI: 0.30 to 43.9, *p* = 0.31), diabetic retinopathy (OR = 1.81, CI: 0.80 to 4.11, *p* = 0.15), diabetic ketosis (OR = 3.88, CI: 0.86 to 17.35, *p* = 0.076) and diabetic maculopathy (OR = 0.87, CI: 0.09 to 7.71, *p* = 0.90). There was no sufficient data to calculate heterogeneity and polymorphism in this aspect (Figure 2).

However, there were no causal relationships between plasma VitC with diabetic complications, such as diabetic nephropathy (OR = 0.95, CI: 0.71 to 1.26, *p* = 0.72), diabetic neuropathy (OR = 0.69, CI: 0.42 to 1.12, *p* = 0.13), diabetic retinopathy (OR = 0.99, CI: 0.86 to 1.15, *p* = 0.92), diabetic ketosis (OR = 0.86, CI: 0.63 to 1.16, *p* = 0.32), diabetic hypoglycemia (OR = 1.06, CI: 0.77 to 1.45, *p* = 0.72), diabetic maculopathy (OR = 0.69, CI: 0.47 to 1.00, *p* = 0.06) (Figure 2 and Appendix S2). The results of MR‐Egger, weighted median, simple mode, and weighted mode analyses were similar with those of the IVW. Several sensitivity analyses were performed to further investigate the potential pleiotropy between exposure and outcome. The MR‐Egger intercepts did not provide evidence of horizontal pleiotropy, and neither did MR‐PRESSO identify outliers, and no heterogeneity was detected by Cochran's *Q* test (Table 3). The results of the leave‐one‐variant‐out analysis did not identify any SNP within VitC that had a significant influence on the results of MR studies which means that excluded exaggerated influence on the combined effect (Appendix S4). The funnel plot presents a relatively symmetrical distribution of variant effects, indicating no directional pleiotropy (Appendix S3). The scatter plot was listed in Appendix S5.

**TABLE 3:** Sensitivity analysis of the causal association between vitamin C, vitamin D, and diabetic complications.

Exposure	Outcome	Methods^b^	Heterogeneity		Horizontal pleiotropy test		MR‐PRESSO outlier test					
			Cochran *Q* test				Before correction			After correction^a^		
			*Q* value	*p*	MR‐Egger intercept	*p*	MR analysis causal estimate	SD	*p*	MR analysis causal estimate	SD	*p*
Vitamin C	Diabetic nephropathy	MR‐Egger	3.233	0.919	−0.004	0.795	−0.052	0.088	0.572	NA	NA	NA
		IVW	3.305	0.951								
	Diabetic neuropathy	MR.Egger	8.461	0.390	−0.044	0.132	−0.378	0.251	0.166	NA	NA	NA
		IVW	11.434	0.247								
	Diabetic retinopathy	MR.Egger	4.627	0.797	0.005	0.543	−0.007	0.055	0.900	NA	NA	NA
		IVW	5.032	0.832								
	Diabetic hypoglycemia	MR.Egger	11.435	0.178	−0.007	0.734	0.057	0.160	0.730	NA	NA	NA
		IVW	11.612	0.236								
	Diabetic maculopathy	MR.Egger	3.868	0.869	−0.007	0.752	−0.373	0.129	0.018	NA	NA	NA
		IVW	3.975	0.913								
	Diabetic ketosis	MR‐Egger	11.614	0.169	−0.010	0.609	−0.154	0.155	0.345	NA	NA	NA
		IVW	12.024	0.212								
Vitamin D	Diabetic nephropathy	MR‐Egger	180.823	0.103	−0.008	0.109	0.172	0.148	0.248	NA	NA	NA
		IVW	183.792	0.087								
	Diabetic neuropathy	MR‐Egger	177.570	0.149	0.003	0.689	0.266	0.222	0.232	NA	NA	NA
		IVW	177.750	0.160								
	Diabetic retinopathy	MR‐Egger	222.058	0.001	−0.004	0.124	−0.111	0.082	0.177	NA	NA	NA
		IVW	225.427	0.000								
	Diabetic hypoglycemia	MR‐Egger	227.366	0.001	−0.005	0.360	−0.162	0.155	0.296	−0.140	0.152	0.357
		IVW	228.532	0.001								
	Diabetic maculopathy	MR‐Egger	180.192	0.090	−0.005	0.465	−0.393	0.213	0.067	−0.319	0.199	0.111
		IVW	180.813	0.094								
	Diabetic ketosis	MR‐Egger	185.835	0.058	−0.004	0.334	−0.269	0.146	0.067	−0.237	0.138	0.088
		IVW	186.265	0.062								

^a^ The after correction were performed if necessary.

^b^ IVW: Inverse variance weighted.

Genetically predicted plasma VitD levels were associated with diabetic ketosis (OR = 0.74, CI: 0.55 to 0.99, *p* = 0.04). The levels of VitD in plasma were not significantly associated with diabetic complications (Figure 2 and Appendix S6), such as diabetic nephropathy (OR = 1.15, CI: 0.85 to 1.55, *p* = 0.36), diabetic neuropathy (OR = 1.25, CI: 0.80 to 1.94, *p* = 0.33), diabetic retinopathy (OR = 0.90, CI: 0.82 to 1.90, *p* = 0.13), diabetic hypoglycemia (OR = 0.86, CI: 0.64 to 1.18, *p* = 0.37) and diabetic maculopathy (OR = 0.67, CI: 0.43 to 1.02, *p* = 0.06). The results of MR‐Egger, weighted median, simple mode, and weighted mode analyses were like the IVW. In the sensitivity analysis of VitD and diabetes complications, the MR‐Egger intercepts did not provide evidence of horizontal pleiotropy, the heterogeneity was detected by Cochran's *Q* test in diabetic hypoglycemia, diabetic maculopathy, diabetic ketosis, so we choose the random effects model. To determine the horizontal pleiotropy and correct the potential outliers, the MR‐PRESSO analysis was performed (Verbanck et al. 2018), identifying that the outliers of diabetic ketosis (rs11602347), diabetic hypoglycemia (rs41290120) and diabetic maculopathy (rs11602347 and rs68033110). However, VitD was still not associated with the risk of diabetic ketosis (OR = 0.76, CI: 0.58 to 1.00, *p* = 0.055), diabetic hypoglycemia (OR = 0.867, CI: 0.64 to 1.18, *p* = 0.37) and diabetic maculopathy (OR = 0.72, CI: 0.48 to 1.07, *p* = 0.105) after omitting the potential outliers. Furthermore, other sensitivity analyses were also performed. The robustness of results was confirmed by the leave‐one‐out sensitivity test, turning out that the result was not significantly affected by removing any single SNP (Appendix S8). No directional pleiotropy was detected through a relatively symmetrical distribution of the Funnel plot within VitD and complications (Appendix S7). The Scatter plot (Appendix S9) was also listed.

## 4 Discussion

Although the association of circulating vitamins with diabetes or related complications has been reported in several observational studies, it seemed all based on small sample size. In this study, based on large‐scale GWAS summary statistics, we performed MR analyses to investigate the causal relationship between vitamins and diabetic complications and try to demonstrate the vitamins in regulating blood glucose or decreasing complications.

As a form of VitA in plasma, retinol has been acknowledged for its critical function in embryonic development both in vision and the nervous system. Some studies have shown the relationship between retinol and diabetes. A recent case–control study showed that high retinol intake (100 μg per day) was associated with a lower risk of diabetic retinopathy in T2DM patients (OR: 0.83; CI: 0.70 to 0.98; *p* = 0.032), and they concluded that higher dietary intake of retinol equivalent is a protective factor for diabetic retinopathy (Zhang et al. 2019). However, a limited subject suggests more research to confirm this issue. In addition, animal studies proved that VitA is a protective factor in diabetes. For example, the severity of insulin inflammation in mice was significantly reduced after being treated with VitA, suggesting that VitA can protect against autoimmune inflammatory attacks on the islet beta cells and has the potential to reduce the onset and pathogenesis of autoimmune diabetes (Zunino et al. 2007). Another animal study proved that a VitA‐deficient diet had reduced glucose‐stimulated insulin secretion and a loss of intestinal glucagon‐like peptide‐1 (GLP‐1) expression (Zhou, Zhou, Zhang, et al. 2020), which may be because of the RARβ signaling pathway (Yu et al. 2024). Moreover, VitA plays an essential role in pancreatic homeostasis, because it has been proved that islet function was reduced by activating islet stellate cells (ISCs) in VitA deficiency in mice (Zhou, Zhou, Sun, et al. 2020). However, these studies were all animal studies, and far from clinical evidence of studies in the pathogenesis in the human body. As the level of retinol in plasma was not significantly associated with each diabetic complication through this large sample size MR study, more research is needed to confirm VitA and diabetes complications to guide supplementing or reducing VitA intake.

There were studies evidence of the high level of VitB_6_ improving DM and complications. A clinical study in Japan proved that high VitB_6_ intake was associated with a lower incidence of diabetic retinopathy in T2DM patients (Horikawa et al. 2020). The VitB_6_ (300 mg per day) were proved to decreasing the level of indicator such as the fasting blood glucose (FPG), glycosylated hemoglobin (HbA1c), fasting serum insulin and homeostasis model assessment of insulin resistance (HOMA‐IR) in DM patients (Dawood et al. 2023), and significantly decreasing the postprandial blood glucose in healthy individuals (Kim et al. 2019). There were studies showed that lower level of VitB_6_ may be associated with the development of diabetes (Ahn et al. 2011). Also, it had been proved that there is a strong negative correlation between FPG levels and pyridoxine (VitB_6_) deficiency. The nerve conduction velocity was proved significantly reduced in VitB_6_ deficient patients and which would inducing diabetic peripheral neuropathy (Khobrani et al. 2023).

There were some potential mechanisms of VitB_6_ to improve DM and complications. Firstly, as a structural or functional pattern of the vascular changes after hyperglycemia, the formation of advanced glycation end products (AGEs) is one of the main leading events, and the AGEs are broadly present in the serum, vasculature, retina, and renal compartments of DM patients. The VitB_6_ was proved to eliminate the AGEs so as to prevent diabetic complications (Vernì 2024). Besides, VitB_6_ is also an important coenzyme in the metabolism of Homocysteine (Hcy), which was deemed a toxic factor in damaging the endothelial cells in microvessels and inducing vascular diseases through hyperhomocysteinemia (HHcy) (Sen and Tyagi 2010). VitB_6_ can work together with other nutrients to degrade Hcy by methylation (Zhu et al. 2024); as a result, decreasing the related vascular disease risks.

There is a paradox about VitB_6_ seemingly. The VitB_6_ is associated with an increased risk of diabetic hypoglycemia in this MR study. On the other hand, the previous evidence indicates that VitB_6_ supplementation could reduce blood glucose levels within surgical intensive care patients (Hou et al. 2011). There is seemingly a paradox in terms of the patient's gains and losses; however, levels of VitB_6_ and glucose levels were consistently negatively correlative here. Therefore, the subsequent blood glucose levels decreased both normally, which is deemed a positive aspect delaying related neurovascular complications, and abnormally, which is deemed a negative aspect suffering hypoglycemia and its comorbidity, such as falling fractures or even hypoglycemic coma. Deducing, there may also be a U‐shaped relationship of VitB_6_ where both deficiency and excess are problematic in blood glucose levels (Wang et al. 2022). Similarly, the VitB_6_ has been found to lower blood sugar in animal studies; the level of blood glucose in rats was significantly reduced after taking VitB_6_, and the oral glucose tolerance tests also showed that VitB_6_ has hypoglycemic effects (Abdullah et al. 2019).

There were also some potential mechanisms regarding VitB_6_ and hypoglycemia. Firstly, as VitB_6_ acts as a coenzyme in over 100 enzymatic reactions, gluconeogenesis and glycogenolysis are included in its regulation (Brown et al. 2025), and because of its potential hypoglycemic activity, the relation between VitB_6_ and hypoglycemia are rational. In addition, normally, Phosphoinositide 3‐kinase (PI3K) regulates insulin signaling, and its inactivating mutations are associated with insulin resistance (IR). Activity VitB_6_ rescues DNA damage treating impaired PI3K, so it can enhance insulin sensitivity and attenuating insulin resistance (Mascolo et al. 2022), and VitB_6_ has been proved that VitB_6_ can induced autophagy inhibiting the apoptosis of β‐cells (Zhang et al. 2022) via the mTOR‐dependent pathway. However, excessive VitB_6_, by inference, may overly elaborate the above process, cause hypoglycemia, which needs more study to elusive the true mechanism. Furthermore, in case reports (Bacharach et al. 2017; van Hunsel et al. 2018), clinical studies (Li, Chen, et al. 2021), and even animal studies (Sharp and Fedorovich 2015), it has been found that high doses and long‐term use of VitB_6_ supplements may lead to neuropathy and other injured symptoms. Also, reverse MR of diabetic hypoglycemia and VitB_6_, was performed suggesting that there is no potential reverse causality (Appendix S10). How the exact mechanism of VitB_6_ form hypoglycemic to hypoglycemia is not known calling future research.

VitC is an effective water‐soluble antioxidant and is also known as ascorbic acid. Many studies have shown that the level of VitC is closely related to the occurrence and development of diabetes. A shorter survival time was seen in diabetics with low levels of VitC in serum than in other patients (Sun, Karp, et al. 2022). It is also reported that the risk of diabetes was increased in the future in prediabetes for those who lack VitC (Das 2019). In addition, a study has shown that VitC supplementing can regulate fasting blood sugar (FBG), HbA1c, and improve insulin resistance in DM patients (Mason et al. 2016).

It is one of the most common and serious diabetic complications is diabetic retinopathy (DR) (Antonetti et al. 2012), which include the diabetic macular edema (DME). A DME can occur at any stage of DR and damage the visual function. Importantly, the VitC is deemed correlation with DR and DME. The level of circulating VitC has been found lower within DR patients than others in a cross‐sectional study (Xiong et al. 2022). Studies have shown that retinopathy and other diabetic complications were all mediated by hyperglycemia‐induced cell damage, reactive oxygen species (ROS), and oxidative stress (Barman and Srinivasan 2017; Malepati and Grant 2025). So, the possibly mechanism is that the antioxidants and the enzymes who responsible for the metabolism such as superoxide dismutase (SOD), glutathione reeducates, glutathione peroxidase, and catalase are suffered damage in retinal cells (Haskins et al. 2003). A randomized controlled trial investigated the utility of antioxidants native to healthy retinas and the effect of their supplementation on progression of macular degeneration, turned out that the consumption of higher quantities of antioxidants had strong prevention in disease progression compared to placebo after mean follow‐up of 6.3 years (Evans and Lawrenson 2023).

This study showed there was no correlation between VitC level and diabetic complications. The previous studies try to conclude the connection of VitC in DR patients. There was a significant positive correlation be found between VitC (Fahmy et al. 2021) and macular thickness, and the gradually increased thickness of macular were also correlate with the progression of DM (Weinberger et al. 1998). Additionally, a retrospective review was conducted with 479 patients with non‐proliferative DR (NPDR), the incidence of diabetic macular degeneration was reduced in patients taking oral statins, and the reduction was even more significant when VitC intake was taken into account (Gurreri et al. 2019). Identically, vitreous level of VitC in proliferative DR (PDR) patients observed a tenfold decrease, meaning the degree of macular ischemia is also associated with VitC (Park et al. 2019), showed the VitC supplementation can prevent macular ischemia to a certain extent. Therefore, for early detection and prevention of DME, it is advisable for diabetic patients to have a diet rich in antioxidants such as VitC. However, consistent with the previous studies, there were no association between VitC levels and DR (Sasaki et al. 2015; She et al. 2021). Further high‐quality clinical trials are necessary to clarify the benefit of VitC supplementation to prevent DR.

According to the previous study, there have been reported relationships between VitD and diabetic complications. A retrospective study showed that 25(OH)D concentrations were negatively correlated with insulin resistance (Dong et al. 2024) and HOMA‐IR in DM patients without osteoporosis (Zhang et al. 2021). Okuyan et al. (2024) also reported the relationship between VitD, inflammatory markers, and insulin resistance in childhood; it turns out that VitD deficiency is related to increased levels of circulating inflammatory markers and decreased insulin sensitivity, suggesting that VitD supplementation may improve insulin sensitivity in patients (Sacerdote et al. 2019). Moreover, higher VitD was also reported to be associated with the risk of diabetic microvascular complications, such as DR, diabetic nephropathy, and diabetic neuropathy. Clinical studies had proved that T2DM patients with microvascular complications had lower VitD levels, and lower VitD was independently associated with the presence of microvascular complications (Maamar El Asri et al. 2022; Zoppini et al. 2015). Moreover, a prospective observational clinical study showed that patients with DR had significantly lower VitD levels than others (Zhuang et al. 2024). In addition, a meta‐analysis involving 10,007 participants showed a statistically significant reduction in VitD in patients with DR compared with controls (Zhang et al. 2017). However, a cross‐sectional study of 815 patients with T2DM showed that the level of 25(OH)D was related to diabetic peripheral neuropathy and diabetic nephropathy, but without correlation with DR (Zhao et al. 2021).

Although the VitD level was not significantly associated with diabetic complications through this large sample size MR study, the result needs to be treated with caution. There were still pre‐adjustment trends (e.g., ketoacidosis *p* = 0.04) in appearing significant results. According to experimental evidence of VitD in modulating pancreatic resilience and inflammatory cascades (Okuyan et al. 2024). Although inconclusive until now, potential dose‐dependent or context‐specific effects that may attain significance in expanded cohorts through future research with large sample size, deserves more deep research in these pathophysiological mechanisms in VitD and DM patients and their complications.

There were some limitations in this study. Firstly, the results were only based on participants of European ancestry, and its conclusions may not be suitable for other ancestries around the world. Limits to MR studies themselves should be considered (such as limited numbers of instrument variables). Secondly, only 4 common vitamins and not all the diabetic complications (e.g., diabetic cardiovascular disease) were included, and further investigation was needed. Moreover, the statistical power may be relatively insufficient with a wide CI associated with VitB_6_ and hypoglycemia, reflecting the possibility of overestimation. Also, a single SNP was employed, taking into account the possibility of instrument bias. So, the result needs to be treated with caution. Future research involving larger datasets is needed to suit the statistical power and reveal a precise relation.

However, there were still several strengths in this study. Firstly, this is the first MR study to explore the relationship between vitamins and diabetic complications including diabetic hypoglycemia, diabetic macular degeneration, and diabetic ketosis, and causal relationships were explored. Secondly, through the MR analysis method, the impact of confounding factors and reverse causality can be minimized. The IVs were obtained from the large‐scale available GWAS datasets, and the *F* statistics for each of the instrumental variables were more than 10, indicating little instrumental variable bias. Additionally, we hint at the need for vitamin supplementation in people with diabetic complications, which is a useful evaluation and reference clinically. Finally, this research also discussed current research and pointed out future research directions.

## 5 Conclusion

In conclusion, this MR analysis provided genetic evidence that plasma VitB_6_ may be the risk factor for diabetic hypoglycemia. No other vitamins' effects were found in any further diabetic complications such as maculopathy, ketoacidosis, hypoglycemia, neuropathy, nephropathy, and retinopathy. Monitoring VitB_6_ and supplements might be important. On the contrary, the effects of other vitamins and complication prevention were limited. The findings will help identify the mechanisms of how vitamins act as influential factors for diabetic complications and provide potential implications in the prognosis and treatment further. However, a stricter attitude should be taken toward results and calling for large amounts of sample size studies both in basic mechanisms research and clinical research in the future to confirm and identify the mechanisms of these causal links.

## Author Contributions

**Sijia Cai:** conceptualization (equal), data curation (equal), formal analysis (equal), funding acquisition (equal), investigation (equal), methodology (equal), project administration (equal), resources (equal), software (equal), supervision (equal), validation (equal), visualization (equal), writing – original draft (equal), writing – review and editing (equal). **Weitao Man:** data curation (equal), investigation (equal). **Wenqing Liu:** data curation (equal), investigation (equal). **Bowu Li:** data curation (equal), investigation (equal). **Zhongchen He:** data curation (equal), investigation (equal). **Guman Duan:** conceptualization (equal), data curation (equal), formal analysis (equal), funding acquisition (equal), investigation (equal), methodology (equal), project administration (equal), resources (equal), software (equal), supervision (equal), validation (equal), visualization (equal), writing – original draft (equal), writing – review and editing (equal).

## Ethics Statement

The authors have nothing to report, since the study is based on summary‐level data. In all original studies, ethical approval and consent to participate had been obtained.

## Consent

Written informed consent for publication was obtained from all participants.

## Conflicts of Interest

The authors declare no conflicts of interest.

## Supporting information


**Appendix S1.** Detailed information about SNPs in this study.

**Appendix S2.** Forest plot of vitamin C for Diabetic complications, such as (A) Diabetic hypoglycemia, (B) Diabetic ketoacidosis, (C) Diabetic maculopathy, (D) Diabetic nephropathy, (E) Diabetic neuropathy, and (F) Diabetic retinopathy.

**Appendix S3.** Funnel plot of vitamin C for Diabetic complications, such as (A) Diabetic hypoglycemia, (B) Diabetic ketoacidosis, (C) Diabetic maculopathy, (D) Diabetic nephropathy, (E) Diabetic neuropathy, and (F) Diabetic retinopathy.

**Appendix S4.** Leave−one−out sensitivity analysis for Vitamin C on Diabetic complications, such as (A) Diabetic hypoglycemia, (B) Diabetic ketoacidosis, (C) Diabetic maculopathy, (D) Diabetic nephropathy, (E) Diabetic neuropathy and (F) Diabetic retinopathy.

**Appendix S5.** Scatter plot of vitamin C for Diabetic complications, such as (A) Diabetic hypoglycemia, (B) Diabetic ketoacidosis, (C) Diabetic maculopathy, (D) Diabetic nephropathy, (E) Diabetic neuropathy and (F) Diabetic retinopathy.

**Appendix S6.** Forest plot of vitamin D for Diabetic complications, such as (A) Diabetic hypoglycemia, (B) Diabetic ketoacidosis, (C) Diabetic maculopathy, (D) Diabetic nephropathy, (E) Diabetic neuropathy, and (F) Diabetic retinopathy.

**Appendix S7.** Funnel plot of vitamin D for Diabetic complications, such as (A) Diabetic hypoglycemia, (B) Diabetic ketoacidosis, (C) Diabetic maculopathy, (D) Diabetic nephropathy, (E) Diabetic neuropathy, and (F) Diabetic retinopathy.

**Appendix S8.** Leave−one−out sensitivity analysis for Vitamin D on Diabetic complications, such as (A) Diabetic hypoglycemia, (B) Diabetic ketoacidosis, (C) Diabetic maculopathy, (D) Diabetic nephropathy, (E) Diabetic neuropathy and (F) Diabetic retinopathy.

**Appendix S9.** Scatter plot of vitamin D for Diabetic complications, such as (A) Diabetic hypoglycemia, (B) Diabetic ketoacidosis, (C) Diabetic maculopathy, (D) Diabetic nephropathy, (E) Diabetic neuropathy and (F) Diabetic retinopathy.

**Appendix S10.** The STROBE‐MR checklist.

**Appendix S11.** Detailed results and sensitive analysis about reverse mendelian randomization of diabetic hypoglycemia and VitB6.

## Data Availability

All data in this study are available through publicly accessible data bases. Summary GWAS statistics related to dietary vitamins are publicly available at https://gwas.mrcieu.ac.uk. The FinnGen Consortium GWAS summary statistics for diabetic complications are available at https://www.finngen.fi/en.
