# Supplementary material for: Causal Relationship Between Various Vitamins and Different Diabetic Complications: A Mendelian Randomization Study
Source: Food Sci Nutr. 2025 Jul 7;13(7):e70536. doi: 10.1002/fsn3.70536 (PMC12230352; doi:10.1002/fsn3.70536)
Supplement: Supplementary file 1 — Appendix S1. Detailed information about SNPs in this study. [file FSN3-13-e70536-s003.docx]

Supplementary material 1: Detailed information about SNPs in this study.

| Exposure |  | Chr | Pos | Effect allele | Other allele | EAF | Beta | SE | P | Sample Size | R^2^ | F-statistic |  |
| --- | --- | --- | --- | --- | --- | --- | --- | --- | --- | --- | --- | --- | --- |
| Vitamin A | rs10882272 | 10 | 95348182 | C | T | 0.350 | -0.030 | 0.004 | 6.51E-15 | 5006 | 0.0111 | 56.228 |  |
| Vitamin A | rs1667255 | 18 | 29187279 | C | A | 0.310 | 0.030 | 0.004 | 6.35E-14 | 5006 | 0.0111 | 56.228 |  |
| Vitamin B_6_ | rs155599 | 2 | 158307375 | C | T | 0.705 | 0.034 | 0.006 | 1E-08 | 64979 | 0.0005 | 32.758 |  |
| Vitamin C | rs6693447 | 1 | 2330190 | T | G | 0.551 | 0.039 | 0.006 | 6.25E-10 | 52018 | 0.0008 | 42.248 |  |
|  | rs33972313 | 5 | 138715502 | C | T | 0.968 | 0.360 | 0.018 | 4.61E-90 | 52018 | 0.0076 | 399.98 |  |
|  | rs10051765 | 5 | 176799992 | C | T | 0.342 | 0.039 | 0.007 | 3.64E-09 | 52018 | 0.0006 | 31.04 |  |
|  | rs7740812 | 6 | 52725787 | G | A | 0.594 | 0.038 | 0.006 | 1.88E-09 | 52018 | 0.0008 | 40.11 |  |
|  | rs117885456 | 12 | 96249111 | A | G | 0.087 | 0.078 | 0.012 | 1.70E-11 | 52018 | 0.0008 | 42.248 |  |
|  | rs2559850 | 12 | 102093459 | A | G | 0.598 | 0.058 | 0.006 | 6.30E-20 | 52018 | 0.0018 | 93.441 |  |
|  | rs10136000 | 14 | 105253581 | A | G | 0.283 | 0.040 | 0.007 | 1.33E-08 | 52018 | 0.0006 | 32.652 |  |
|  | rs56738967 | 16 | 79740541 | C | G | 0.321 | 0.041 | 0.007 | 7.62E-10 | 52018 | 0.0007 | 34.305 |  |
|  | rs9895661 | 17 | 59456589 | C | G | 0.321 | 0.041 | 0.007 | 7.62E-10 | 52018 | 0.0007 | 34.305 |  |
| Vitamin D | rs10017024 | 4 | 57744248 | C | T | 0.505 | -0.011 | 0.002 | 1E-200 | 441291 | 0.0001 | 31.711 |  |
|  | rs10045097 | 5 | 143838590 | A | G | 0.384 | -0.011 | 0.002 | 1E-200 | 441291 | 0.0001 | 29.526 |  |
|  | rs10070734 | 5 | 87940026 | C | T | 0.704 | 0.012 | 0.002 | 1E-200 | 441291 | 0.0001 | 31.562 |  |
|  | rs1022669 | 14 | 29737713 | G | A | 0.589 | -0.010 | 0.002 | 1E-200 | 441291 | 0.0001 | 27.721 |  |
|  | rs1026337 | 4 | 69528250 | T | A | 0.103 | -0.022 | 0.003 | 1E-200 | 441291 | 0.0001 | 41.013 |  |
|  | rs1047891 | 2 | 211540507 | A | C | 0.316 | -0.014 | 0.002 | 1E-200 | 441291 | 0.0001 | 46.042 |  |
|  | rs10518055 | 4 | 69301762 | G | T | 0.184 | -0.014 | 0.003 | 1E-200 | 441291 | 0.0001 | 25.89 |  |
|  | rs10771085 | 12 | 24561938 | T | C | 0.567 | -0.011 | 0.002 | 1E-200 | 441291 | 0.0001 | 28.632 |  |
|  | rs10876448 | 12 | 53779321 | G | A | 0.175 | -0.013 | 0.003 | 1E-200 | 441291 | 0.0001 | 25.741 |  |
|  | rs10888010 | 1 | 17557039 | C | T | 0.428 | -0.012 | 0.002 | 1E-200 | 441291 | 0.0001 | 39.174 |  |
|  | rs11060406 | 12 | 123339117 | T | C | 0.037 | -0.027 | 0.005 | 1E-200 | 441291 | 0.0001 | 26.946 |  |
|  | rs11150592 | 16 | 30555658 | A | G | 0.841 | -0.014 | 0.003 | 1E-200 | 441291 | 0.0001 | 27.499 |  |
|  | rs111757536 | 11 | 70509171 | A | G | 0.027 | 0.034 | 0.006 | 1E-200 | 441291 | 0.0001 | 31.875 |  |
|  | rs11209943 | 1 | 72750500 | G | A | 0.599 | -0.010 | 0.002 | 1E-200 | 441291 | 0.0001 | 27.807 |  |
|  | rs11227307 | 11 | 65581135 | A | G | 0.647 | 0.012 | 0.002 | 1E-200 | 441291 | 0.0001 | 32.156 |  |
|  | rs11253202 | 10 | 5530385 | C | T | 0.211 | 0.012 | 0.002 | 1E-200 | 441291 | 0.0001 | 25.668 |  |
|  | rs114165281 | 4 | 99798284 | T | C | 0.028 | 0.037 | 0.006 | 1E-200 | 441291 | 0.0001 | 37.546 |  |
|  | rs114165349 | 1 | 27021913 | C | G | 0.023 | -0.036 | 0.007 | 1E-200 | 441291 | 0.0001 | 29.783 |  |
|  | rs114188086 | 4 | 74516128 | A | G | 0.035 | 0.028 | 0.005 | 1E-200 | 441291 | 0.0001 | 27.666 |  |
|  | rs114952461 | 4 | 72203140 | C | T | 0.013 | -0.081 | 0.009 | 1E-200 | 441291 | 0.0002 | 85.082 |  |
|  | rs1149555 | 7 | 133062665 | A | G | 0.802 | -0.012 | 0.002 | 1E-200 | 441291 | 0.0001 | 25.461 |  |
|  | rs11582620 | 1 | 152286126 | G | A | 0.122 | -0.015 | 0.003 | 1E-200 | 441291 | 0.0001 | 26.43 |  |
|  | rs11591147 | 1 | 55505647 | T | G | 0.018 | 0.047 | 0.007 | 1E-200 | 441291 | 0.0001 | 40.823 |  |
|  | rs11600578 | 11 | 15381736 | T | C | 0.040 | -0.048 | 0.005 | 1E-200 | 441291 | 0.0002 | 93.686 |  |
|  | rs11602347 | 11 | 2176852 | G | C | 0.407 | 0.010 | 0.002 | 1E-200 | 441291 | 0.0001 | 25.655 |  |
|  | rs11635491 | 15 | 58719741 | A | G | 0.276 | -0.021 | 0.002 | 1E-200 | 441291 | 0.0002 | 93.735 |  |
|  | rs11714324 | 3 | 49925741 | T | C | 0.177 | 0.014 | 0.003 | 1E-200 | 441291 | 0.0001 | 27.123 |  |
|  | rs117562170 | 12 | 96367480 | C | A | 0.044 | -0.032 | 0.005 | 1E-200 | 441291 | 0.0001 | 45.915 |  |
|  | rs117939970 | 11 | 14979029 | C | T | 0.035 | -0.027 | 0.005 | 1E-200 | 441291 | 0.0001 | 25.457 |  |
|  | rs118032309 | 11 | 70958267 | C | T | 0.013 | -0.045 | 0.009 | 1E-200 | 441291 | 0.0001 | 26.06 |  |
|  | rs11830764 | 12 | 111515020 | C | G | 0.068 | 0.022 | 0.004 | 1E-200 | 441291 | 0.0001 | 31.532 |  |
|  | rs11885466 | 2 | 213923915 | T | C | 0.075 | -0.019 | 0.004 | 1E-200 | 441291 | 0.0001 | 25.933 |  |
|  | rs11928368 | 3 | 85661265 | T | G | 0.328 | -0.013 | 0.002 | 1E-200 | 441291 | 0.0001 | 37.891 |  |
|  | rs12035012 | 1 | 41750648 | A | C | 0.222 | 0.014 | 0.002 | 1E-200 | 441291 | 0.0001 | 35.488 |  |
|  | rs12423650 | 12 | 96210766 | A | G | 0.105 | -0.016 | 0.003 | 1E-200 | 441291 | 0.0001 | 25.744 |  |
|  | rs12503220 | 4 | 2850142 | A | G | 0.189 | -0.013 | 0.002 | 1E-200 | 441291 | 0.0001 | 29.009 |  |
|  | rs12546526 | 8 | 143587121 | C | T | 0.859 | 0.015 | 0.003 | 1E-200 | 441291 | 0.0001 | 26.534 |  |
|  | rs12554549 | 9 | 112239077 | T | C | 0.065 | 0.021 | 0.004 | 1E-200 | 441291 | 0.0001 | 27.341 |  |
|  | rs12797447 | 11 | 15862968 | T | A | 0.248 | 0.012 | 0.002 | 1E-200 | 441291 | 0.0001 | 26.751 |  |
|  | rs12798050 | 11 | 71223256 | T | C | 0.824 | 0.107 | 0.003 | 1E-200 | 441291 | 0.0038 | 1701.1 |  |
|  | rs12800076 | 11 | 14977481 | T | G | 0.025 | -0.032 | 0.006 | 1E-200 | 441291 | 0.0001 | 26.351 |  |
|  | rs12913937 | 15 | 77316131 | A | G | 0.354 | 0.010 | 0.002 | 1E-200 | 441291 | 0.0001 | 25.856 |  |
|  | rs12997900 | 2 | 58795962 | G | A | 0.262 | -0.012 | 0.002 | 1E-200 | 441291 | 0.0001 | 27.33 |  |
|  | rs13065677 | 3 | 52321788 | T | C | 0.051 | 0.023 | 0.004 | 1E-200 | 441291 | 0.0001 | 26.523 |  |
|  | rs13187496 | 5 | 118613707 | G | T | 0.345 | 0.011 | 0.002 | 1E-200 | 441291 | 0.0001 | 29.071 |  |
|  | rs13197862 | 6 | 80014585 | A | G | 0.130 | 0.015 | 0.003 | 1E-200 | 441291 | 0.0001 | 26.045 |  |
|  | rs133075 | 22 | 41081164 | T | G | 0.553 | 0.010 | 0.002 | 1E-200 | 441291 | 0.0001 | 27.034 |  |
|  | rs139047585 | 4 | 73315042 | T | C | 0.023 | -0.036 | 0.007 | 1E-200 | 441291 | 0.0001 | 25.602 |  |
|  | rs141970801 | 2 | 21152755 | A | G | 0.040 | 0.028 | 0.005 | 1E-200 | 441291 | 0.0001 | 30.99 |  |
|  | rs143375244 | 4 | 71644659 | G | A | 0.013 | -0.048 | 0.009 | 1E-200 | 441291 | 0.0001 | 29.072 |  |
|  | rs143610797 | 20 | 52717229 | A | G | 0.021 | -0.036 | 0.007 | 1E-200 | 441291 | 0.0001 | 26.75 |  |
|  | rs144366778 | 14 | 39542637 | T | C | 0.031 | 0.031 | 0.006 | 1E-200 | 441291 | 0.0001 | 30.121 |  |
|  | rs144628971 | 20 | 52720848 | A | G | 0.031 | 0.033 | 0.006 | 1E-200 | 441291 | 0.0001 | 33.135 |  |
|  | rs145662623 | 4 | 100510550 | A | G | 0.061 | 0.020 | 0.004 | 1E-200 | 441291 | 0.0001 | 25.454 |  |
|  | rs146735006 | 11 | 13520260 | T | C | 0.019 | -0.040 | 0.007 | 1E-200 | 441291 | 0.0001 | 29.61 |  |
|  | rs148747986 | 4 | 72883517 | A | G | 0.020 | 0.042 | 0.007 | 1E-200 | 441291 | 0.0001 | 33.653 |  |
|  | rs150057262 | 19 | 19320825 | G | C | 0.012 | 0.051 | 0.010 | 1E-200 | 441291 | 0.0001 | 28.161 |  |
|  | rs151302903 | 2 | 62857411 | C | G | 0.686 | -0.011 | 0.002 | 1E-200 | 441291 | 0.0001 | 25.52 |  |
|  | rs1564366 | 12 | 21335749 | C | G | 0.785 | 0.012 | 0.002 | 1E-200 | 441291 | 0.0001 | 25.329 |  |
|  | rs16830473 | 3 | 173504091 | C | T | 0.091 | 0.018 | 0.003 | 1E-200 | 441291 | 0.0001 | 26.721 |  |
|  | rs1694929 | 3 | 85002871 | T | C | 0.430 | 0.010 | 0.002 | 1E-200 | 441291 | 0.0001 | 25.346 |  |
|  | rs16961568 | 18 | 28842485 | G | A | 0.044 | -0.026 | 0.005 | 1E-200 | 441291 | 0.0001 | 29.315 |  |
|  | rs16980051 | 19 | 46345886 | C | T | 0.511 | -0.010 | 0.002 | 1E-200 | 441291 | 0.0001 | 25.603 |  |
|  | rs16998964 | 20 | 52738470 | T | C | 0.055 | 0.031 | 0.004 | 1E-200 | 441291 | 0.0001 | 48.984 |  |
|  | rs17185287 | 15 | 63830404 | A | G | 0.157 | -0.014 | 0.003 | 1E-200 | 441291 | 0.0001 | 28.319 |  |
|  | rs17309874 | 11 | 27667236 | A | G | 0.262 | -0.011 | 0.002 | 1E-200 | 441291 | 0.0001 | 26.384 |  |
|  | rs1800440 | 2 | 38298139 | C | T | 0.186 | -0.014 | 0.002 | 1E-200 | 441291 | 0.0001 | 31.437 |  |
|  | rs190343325 | 11 | 70793711 | C | T | 0.032 | -0.030 | 0.006 | 1E-200 | 441291 | 0.0001 | 28.962 |  |
|  | rs1909585 | 3 | 124687460 | T | C | 0.346 | 0.011 | 0.002 | 1E-200 | 441291 | 0.0001 | 25.705 |  |
|  | rs1943681 | 18 | 47134844 | T | A | 0.607 | -0.010 | 0.002 | 1E-200 | 441291 | 0.0001 | 26.515 |  |
|  | rs1957065 | 14 | 39378646 | G | C | 0.075 | 0.021 | 0.004 | 1E-200 | 441291 | 0.0001 | 33.055 |  |
|  | rs2023910 | 7 | 21557854 | A | G | 0.264 | -0.012 | 0.002 | 1E-200 | 441291 | 0.0001 | 28.084 |  |
|  | rs2043084 | 15 | 58682496 | C | G | 0.225 | -0.021 | 0.002 | 1E-200 | 441291 | 0.0002 | 78.991 |  |
|  | rs2068190 | 5 | 148007013 | A | G | 0.437 | 0.010 | 0.002 | 1E-200 | 441291 | 0.0001 | 27.251 |  |
|  | rs2071408 | 14 | 103987078 | A | G | 0.366 | 0.012 | 0.002 | 1E-200 | 441291 | 0.0001 | 32.665 |  |
|  | rs2207132 | 20 | 39142516 | A | G | 0.033 | -0.029 | 0.006 | 1E-200 | 441291 | 0.0001 | 27.957 |  |
|  | rs2229742 | 21 | 16339172 | C | G | 0.104 | -0.026 | 0.003 | 1E-200 | 441291 | 0.0001 | 65.098 |  |
|  | rs2246281 | 6 | 131814278 | T | G | 0.225 | -0.012 | 0.002 | 1E-200 | 441291 | 0.0001 | 28.527 |  |
|  | rs2282619 | 11 | 71183645 | C | T | 0.953 | 0.085 | 0.005 | 1E-200 | 441291 | 0.0007 | 309.26 |  |
|  | rs2295659 | 14 | 101141544 | T | G | 0.130 | -0.015 | 0.003 | 1E-200 | 441291 | 0.0001 | 25.734 |  |
|  | rs2304634 | 16 | 4500544 | T | C | 0.686 | 0.011 | 0.002 | 1E-200 | 441291 | 0.0001 | 25.386 |  |
|  | rs2306390 | 12 | 58002599 | T | C | 0.257 | -0.012 | 0.002 | 1E-200 | 441291 | 0.0001 | 27.089 |  |
|  | rs2519093 | 9 | 136141870 | T | C | 0.184 | -0.015 | 0.003 | 1E-200 | 441291 | 0.0001 | 35.26 |  |
|  | rs2607838 | 10 | 87993073 | G | A | 0.930 | -0.020 | 0.004 | 1E-200 | 441291 | 0.0001 | 26.451 |  |
|  | rs2642438 | 1 | 220970028 | G | A | 0.700 | -0.013 | 0.002 | 1E-200 | 441291 | 0.0001 | 40.109 |  |
|  | rs2843128 | 1 | 2315680 | G | A | 0.516 | 0.010 | 0.002 | 1E-200 | 441291 | 0.0001 | 26.079 |  |
|  | rs2999559 | 1 | 151997199 | C | A | 0.258 | -0.011 | 0.002 | 1E-200 | 441291 | 0.0001 | 26.342 |  |
|  | rs336605 | 3 | 18656350 | T | G | 0.726 | -0.011 | 0.002 | 1E-200 | 441291 | 0.0001 | 25.666 |  |
|  | rs34560261 | 15 | 90734426 | T | C | 0.170 | 0.014 | 0.003 | 1E-200 | 441291 | 0.0001 | 26.015 |  |
|  | rs34871842 | 19 | 58353003 | A | G | 0.084 | 0.019 | 0.003 | 1E-200 | 441291 | 0.0001 | 29.511 |  |
|  | rs3745535 | 19 | 51520487 | C | A | 0.645 | 0.010 | 0.002 | 1E-200 | 441291 | 0.0001 | 26.354 |  |
|  | rs3761077 | 19 | 19325963 | T | G | 0.111 | 0.017 | 0.003 | 1E-200 | 441291 | 0.0001 | 31.273 |  |
|  | rs3814995 | 19 | 36342212 | T | C | 0.312 | -0.015 | 0.002 | 1E-200 | 441291 | 0.0001 | 48.801 |  |
|  | rs3817588 | 2 | 27731212 | C | T | 0.197 | 0.015 | 0.002 | 1E-200 | 441291 | 0.0001 | 35.511 |  |
|  | rs41266415 | 1 | 34684617 | T | A | 0.215 | 0.013 | 0.002 | 1E-200 | 441291 | 0.0001 | 30.719 |  |
|  | rs41290120 | 19 | 45382675 | A | G | 0.050 | 0.028 | 0.004 | 1E-200 | 441291 | 0.0001 | 38.996 |  |
|  | rs4364259 | 4 | 15892159 | A | G | 0.205 | 0.016 | 0.002 | 1E-200 | 441291 | 0.0001 | 42.546 |  |
|  | rs4466239 | 6 | 22755139 | G | A | 0.624 | 0.011 | 0.002 | 1E-200 | 441291 | 0.0001 | 29.459 |  |
|  | rs4553272 | 10 | 21838380 | T | C | 0.477 | -0.010 | 0.002 | 1E-200 | 441291 | 0.0001 | 27.996 |  |
|  | rs4631704 | 1 | 230293530 | T | C | 0.608 | 0.011 | 0.002 | 1E-200 | 441291 | 0.0001 | 29.771 |  |
|  | rs4663871 | 2 | 234581587 | A | G | 0.227 | -0.019 | 0.002 | 1E-200 | 441291 | 0.0001 | 66.036 |  |
|  | rs484195 | 19 | 45421877 | G | A | 0.613 | -0.015 | 0.002 | 1E-200 | 441291 | 0.0001 | 53.737 |  |
|  | rs4971020 | 1 | 150522242 | C | T | 0.647 | 0.011 | 0.002 | 1E-200 | 441291 | 0.0001 | 26.759 |  |
|  | rs512083 | 1 | 46027355 | C | T | 0.461 | 0.010 | 0.002 | 1E-200 | 441291 | 0.0001 | 26.242 |  |
|  | rs513533 | 11 | 116519358 | G | A | 0.103 | -0.020 | 0.003 | 1E-200 | 441291 | 0.0001 | 38.633 |  |
|  | rs537089 | 11 | 120078452 | G | C | 0.080 | -0.022 | 0.004 | 1E-200 | 441291 | 0.0001 | 36.353 |  |
|  | rs56059718 | 15 | 38836777 | A | C | 0.191 | -0.013 | 0.002 | 1E-200 | 441291 | 0.0001 | 25.671 |  |
|  | rs56158152 | 16 | 84734147 | T | G | 0.355 | 0.011 | 0.002 | 1E-200 | 441291 | 0.0001 | 26.293 |  |
|  | rs6018088 | 20 | 35998625 | A | C | 0.116 | -0.015 | 0.003 | 1E-200 | 441291 | 0.0001 | 25.711 |  |
|  | rs60500353 | 1 | 62898984 | T | C | 0.156 | 0.015 | 0.003 | 1E-200 | 441291 | 0.0001 | 31.491 |  |
|  | rs60954647 | 11 | 116566933 | C | T | 0.547 | -0.010 | 0.002 | 1E-200 | 441291 | 0.0001 | 26.591 |  |
|  | rs62215568 | 20 | 52632984 | A | G | 0.023 | 0.035 | 0.007 | 1E-200 | 441291 | 0.0001 | 28.068 |  |
|  | rs62299542 | 4 | 69633226 | A | G | 0.131 | 0.024 | 0.003 | 1E-200 | 441291 | 0.0001 | 65.129 |  |
|  | rs62493791 | 8 | 9168897 | G | T | 0.242 | 0.012 | 0.002 | 1E-200 | 441291 | 0.0001 | 27.612 |  |
|  | rs62568181 | 9 | 107645674 | C | T | 0.104 | 0.016 | 0.003 | 1E-200 | 441291 | 0.0001 | 25.847 |  |
|  | rs6657811 | 1 | 109807283 | T | A | 0.131 | 0.015 | 0.003 | 1E-200 | 441291 | 0.0001 | 28.811 |  |
|  | rs673335 | 11 | 75450576 | C | T | 0.160 | 0.016 | 0.003 | 1E-200 | 441291 | 0.0001 | 39.048 |  |
|  | rs6750649 | 2 | 148353141 | G | T | 0.609 | -0.010 | 0.002 | 1E-200 | 441291 | 0.0001 | 26.165 |  |
|  | rs68033110 | 18 | 57914679 | A | G | 0.247 | -0.012 | 0.002 | 1E-200 | 441291 | 0.0001 | 25.645 |  |
|  | rs6970645 | 7 | 100798274 | G | C | 0.752 | 0.011 | 0.002 | 1E-200 | 441291 | 0.0001 | 25.635 |  |
|  | rs6985620 | 8 | 59370159 | C | T | 0.665 | 0.011 | 0.002 | 1E-200 | 441291 | 0.0001 | 26.667 |  |
|  | rs7027254 | 9 | 125605840 | C | T | 0.147 | 0.014 | 0.003 | 1E-200 | 441291 | 0.0001 | 27.063 |  |
|  | rs7189954 | 16 | 79735047 | T | C | 0.361 | -0.010 | 0.002 | 1E-200 | 441291 | 0.0001 | 26.179 |  |
|  | rs7258060 | 19 | 4332105 | T | A | 0.329 | -0.011 | 0.002 | 1E-200 | 441291 | 0.0001 | 26.378 |  |
|  | rs72651860 | 4 | 72492037 | A | G | 0.010 | -0.114 | 0.010 | 1E-200 | 441291 | 0.0003 | 123.78 |  |
|  | rs72680100 | 14 | 50512347 | G | A | 0.039 | -0.027 | 0.005 | 1E-200 | 441291 | 0.0001 | 28.046 |  |
|  | rs72739147 | 15 | 58571401 | T | A | 0.130 | 0.016 | 0.003 | 1E-200 | 441291 | 0.0001 | 30.206 |  |
|  | rs7297538 | 12 | 33693438 | C | T | 0.357 | 0.011 | 0.002 | 1E-200 | 441291 | 0.0001 | 28.259 |  |
|  | rs7330 | 12 | 12874917 | A | C | 0.588 | -0.011 | 0.002 | 1E-200 | 441291 | 0.0001 | 30.609 |  |
|  | rs7419651 | 2 | 101417047 | A | G | 0.119 | -0.018 | 0.003 | 1E-200 | 441291 | 0.0001 | 35.403 |  |
|  | rs74342059 | 11 | 13390072 | T | C | 0.034 | 0.031 | 0.005 | 1E-200 | 441291 | 0.0001 | 31.418 |  |
|  | rs7511513 | 22 | 50841035 | T | C | 0.343 | -0.010 | 0.002 | 1E-200 | 441291 | 0.0001 | 25.399 |  |
|  | rs75865451 | 6 | 25588285 | A | G | 0.105 | -0.016 | 0.003 | 1E-200 | 441291 | 0.0001 | 26.366 |  |
|  | rs76183418 | 3 | 47352998 | C | T | 0.179 | 0.013 | 0.003 | 1E-200 | 441291 | 0.0001 | 25.434 |  |
|  | rs7640441 | 3 | 125118082 | A | C | 0.241 | 0.013 | 0.002 | 1E-200 | 441291 | 0.0001 | 33.35 |  |
|  | rs7651161 | 3 | 48333546 | C | T | 0.528 | -0.011 | 0.002 | 1E-200 | 441291 | 0.0001 | 30.984 |  |
|  | rs76935526 | 2 | 21386353 | T | C | 0.179 | -0.013 | 0.003 | 1E-200 | 441291 | 0.0001 | 27.426 |  |
|  | rs77194050 | 16 | 70687185 | G | A | 0.052 | 0.023 | 0.004 | 1E-200 | 441291 | 0.0001 | 27.13 |  |
|  | rs77257135 | 4 | 72432363 | A | C | 0.017 | 0.048 | 0.008 | 1E-200 | 441291 | 0.0001 | 38.795 |  |
|  | rs77885114 | 1 | 154974619 | A | G | 0.103 | -0.017 | 0.003 | 1E-200 | 441291 | 0.0001 | 25.486 |  |
|  | rs7794142 | 7 | 106892044 | A | G | 0.521 | 0.011 | 0.002 | 1E-200 | 441291 | 0.0001 | 34.371 |  |
|  | rs78117488 | 12 | 96089917 | T | C | 0.062 | 0.021 | 0.004 | 1E-200 | 441291 | 0.0001 | 26.744 |  |
|  | rs7900214 | 10 | 81965655 | A | G | 0.279 | 0.012 | 0.002 | 1E-200 | 441291 | 0.0001 | 28.776 |  |
|  | rs7924820 | 11 | 12995734 | A | G | 0.044 | -0.024 | 0.005 | 1E-200 | 441291 | 0.0001 | 25.895 |  |
|  | rs79687284 | 1 | 214150821 | C | G | 0.034 | -0.027 | 0.005 | 1E-200 | 441291 | 0.0001 | 25.417 |  |
|  | rs7981402 | 13 | 60676803 | A | G | 0.340 | 0.010 | 0.002 | 1E-200 | 441291 | 0.0001 | 25.849 |  |
|  | rs800531 | 8 | 116843273 | C | T | 0.777 | -0.013 | 0.002 | 1E-200 | 441291 | 0.0001 | 29.571 |  |
|  | rs80067728 | 4 | 72545970 | G | A | 0.013 | 0.054 | 0.009 | 1E-200 | 441291 | 0.0001 | 37.441 |  |
|  | rs80204526 | 18 | 47066144 | A | C | 0.011 | -0.048 | 0.009 | 1E-200 | 441291 | 0.0001 | 26.201 |  |
|  | rs897438 | 18 | 61233124 | G | A | 0.771 | -0.012 | 0.002 | 1E-200 | 441291 | 0.0001 | 26.38 |  |
|  | rs942380 | 6 | 121854778 | G | A | 0.595 | 0.011 | 0.002 | 1E-200 | 441291 | 0.0001 | 33.314 |  |
|  | rs951914 | 8 | 25878995 | C | G | 0.711 | -0.011 | 0.002 | 1E-200 | 441291 | 0.0001 | 27.814 |  |
|  | rs9536961 | 13 | 55678332 | G | A | 0.347 | -0.012 | 0.002 | 1E-200 | 441291 | 0.0001 | 32.27 |  |
|  | rs982890 | 11 | 13897749 | C | T | 0.473 | 0.014 | 0.002 | 1E-200 | 441291 | 0.0001 | 51.826 |  |
|  | rs9861009 | 3 | 141654685 | C | T | 0.722 | 0.012 | 0.002 | 1E-200 | 441291 | 0.0001 | 32.336 |  |
|  | rs9889884 | 17 | 66394054 | C | T | 0.756 | 0.013 | 0.002 | 1E-200 | 441291 | 0.0001 | 33.126 |  |
|  | rs9926530 | 16 | 72723613 | G | T | 0.943 | 0.022 | 0.004 | 1E-200 | 441291 | 0.0001 | 26.208 |  |
|  | rs9928757 | 16 | 20352863 | C | G | 0.203 | -0.013 | 0.002 | 1E-200 | 441291 | 0.0001 | 29.235 |  |
|  | rs9989419 | 16 | 56985139 | G | A | 0.605 | -0.012 | 0.002 | 1E-200 | 441291 | 0.0001 | 36.956 |  |
|  | rs9998874 | 4 | 71724362 | G | T | 0.064 | 0.027 | 0.004 | 1E-200 | 441291 | 0.0001 | 45.81 |  |
| 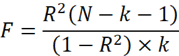  R^2^=2×EAF×(1−EAF)×Beta^2^/[(2×EAF×(1−EAF)×Beta^2^+2×EAF×(1−EAF)×N×SE^2^)]   \|  \| \| --- \| |  |  |  |  |  |  |  |  |  |  |  |  |  |
|  |  |  |  |  |  |  |  |  |  |  |  |  |  |
|  |  |  |  |  |  |  |  |  |  |  |  |  |  |
|  |  |  |  |  |  |  |  |  |  |  |  |  |  |
| The F-statistics were calculated as follow,where R^2^ is the proportion of variance explained in the instrument, k is the number of IVs included in the instrument. N is Sample Size. | | | | | | | | | | | | |  |
|  |  |  |  |  |  |  |  |  |  |  |  |  |  |
