# Supplementary material for: Causal Relationship Between Various Vitamins and Different Diabetic Complications: A Mendelian Randomization Study
Source: Food Sci Nutr. 2025 Jul 7;13(7):e70536. doi: 10.1002/fsn3.70536 (PMC12230352; doi:10.1002/fsn3.70536)
Supplement: Supplementary file 2 — Appendix S2. Forest plot of vitamin C for Diabetic complications, such as (A) Diabetic hypoglycemia, (B) Diabetic ketoacidosis, (C) Diabetic maculopathy, (D) Diabetic nephropathy, (E) Diabetic neuropathy, and (F) Diabetic retinopathy. [file FSN3-13-e70536-s008.docx]

(A) Forest plot of VitC for Diabetic hypoglycemia


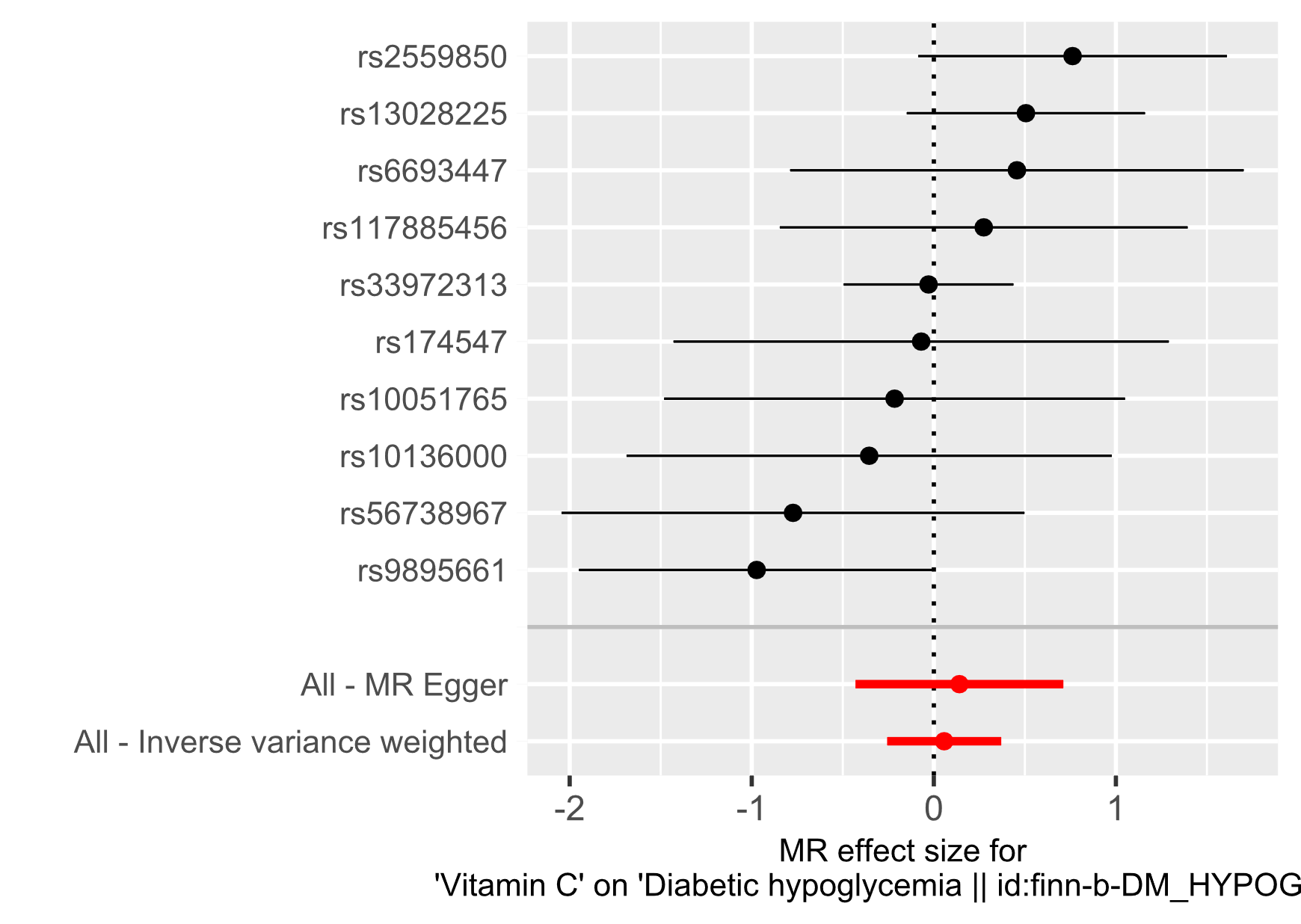


(B) Forest plot of VitC for Diabetic ketoacidosis


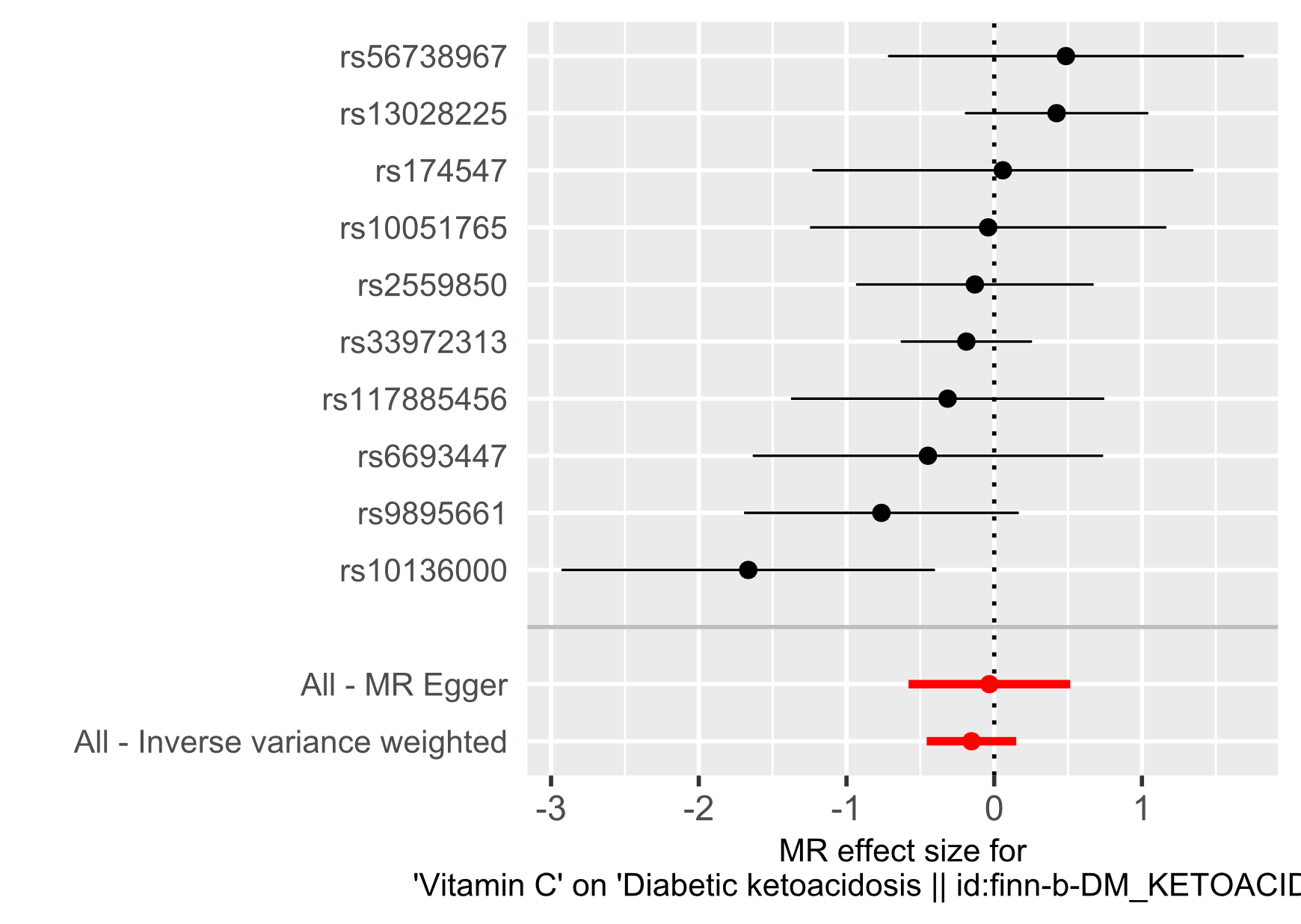


(C) Forest plot of VitC for Diabetic maculopathy


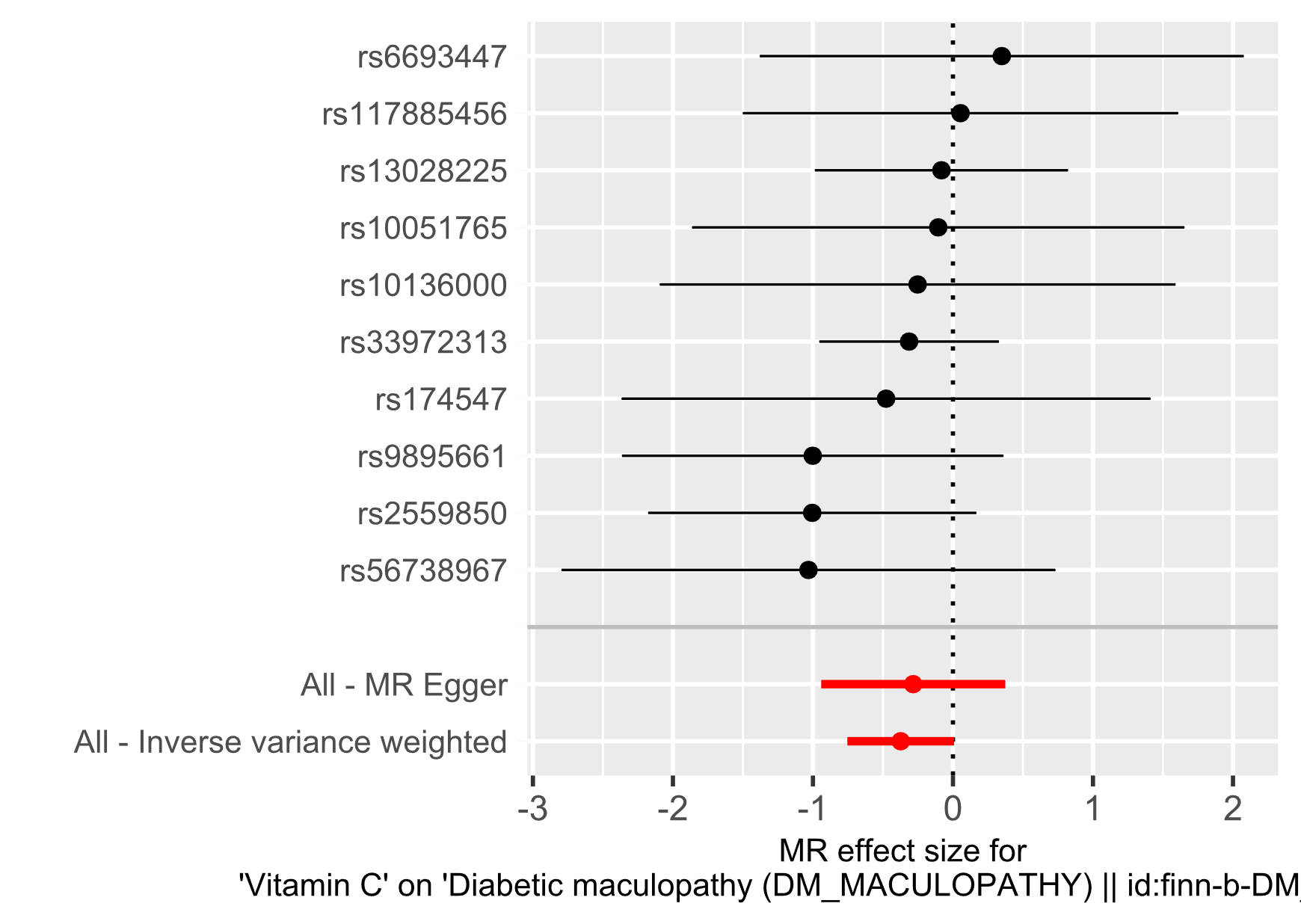


(D) Forest plot of VitC for Diabetic nephropathy


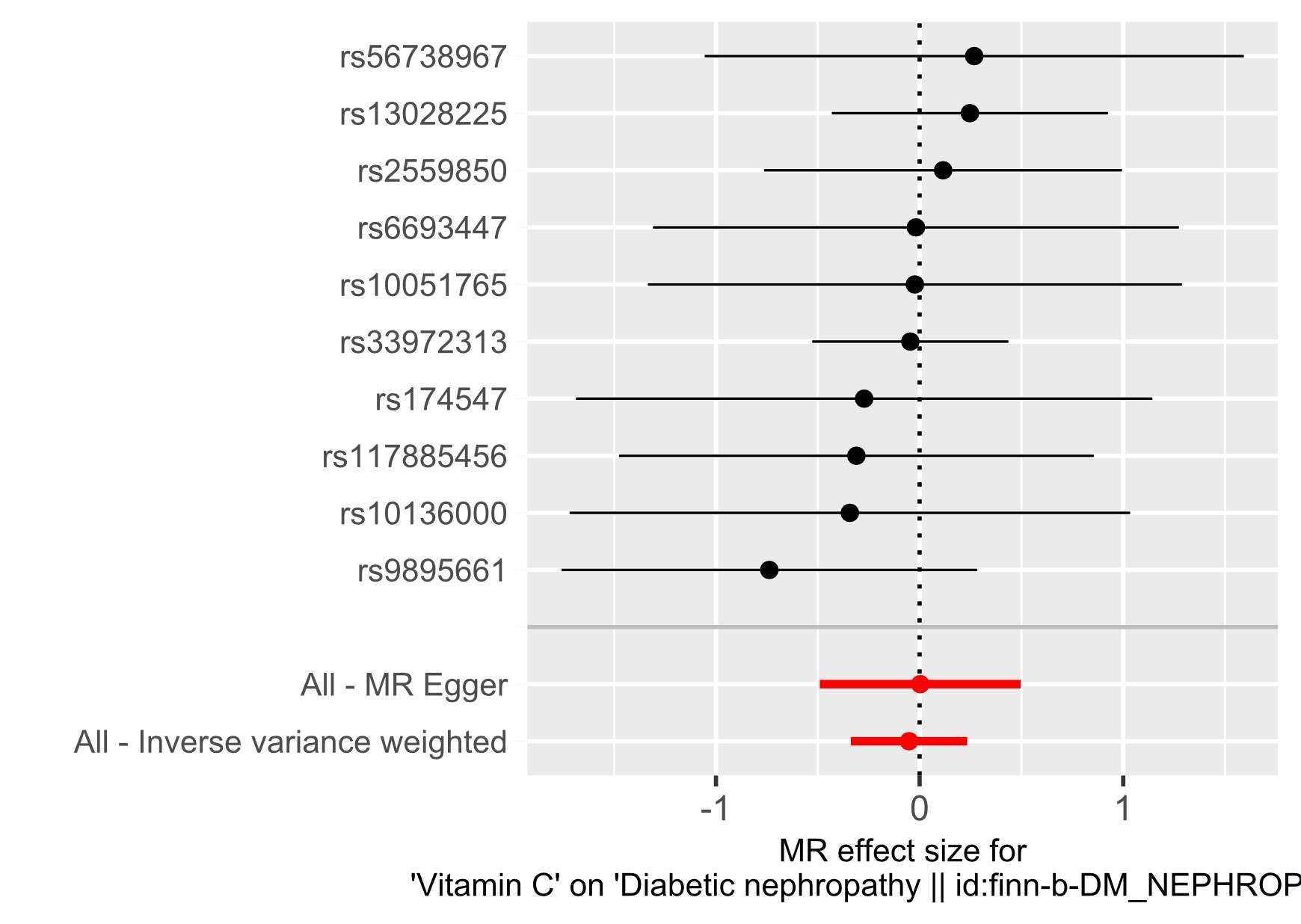


(E) Forest plot of VitC for Diabetic neuropathy


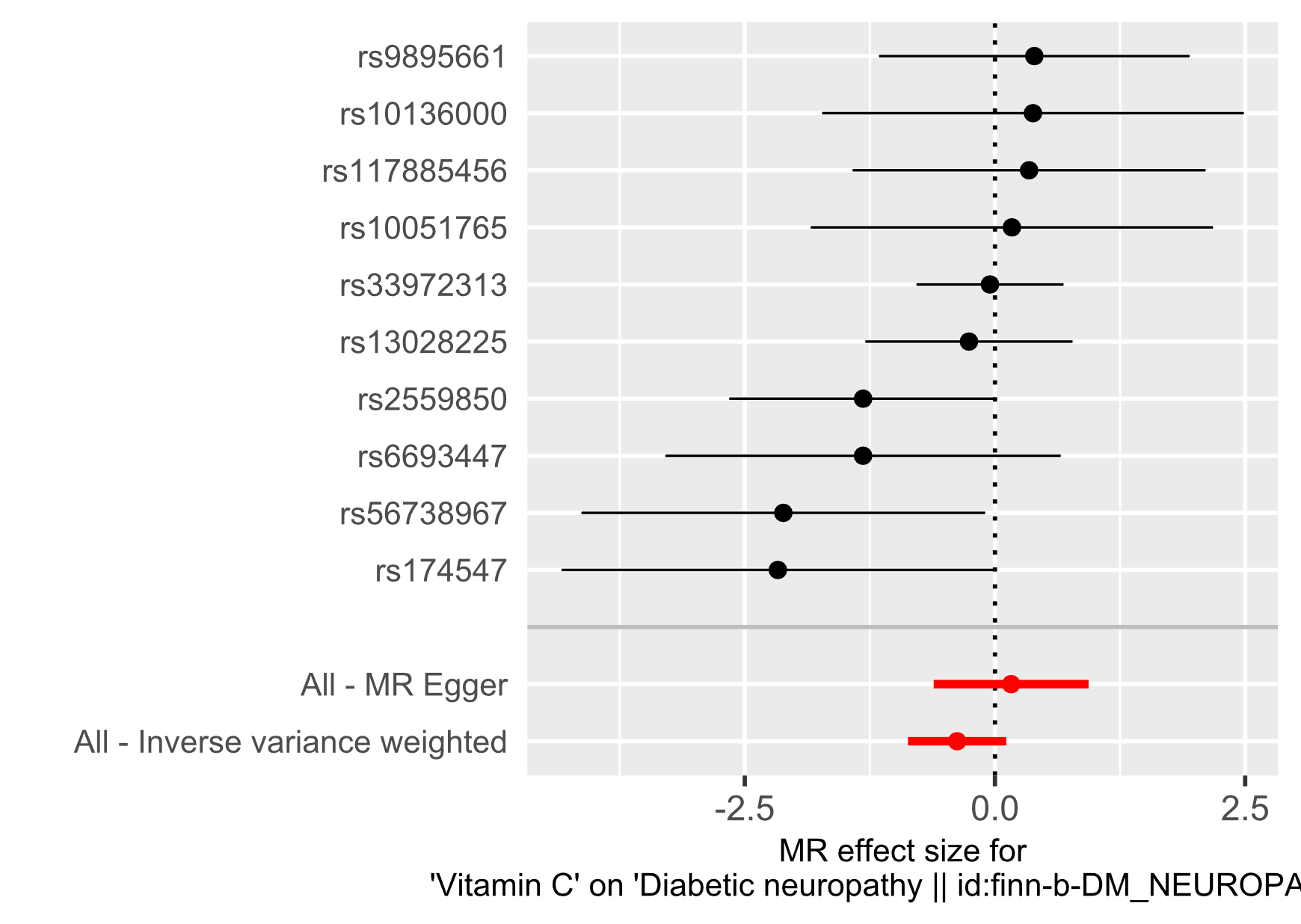


(F) Forest plot of VitC for Diabetic retinopathy


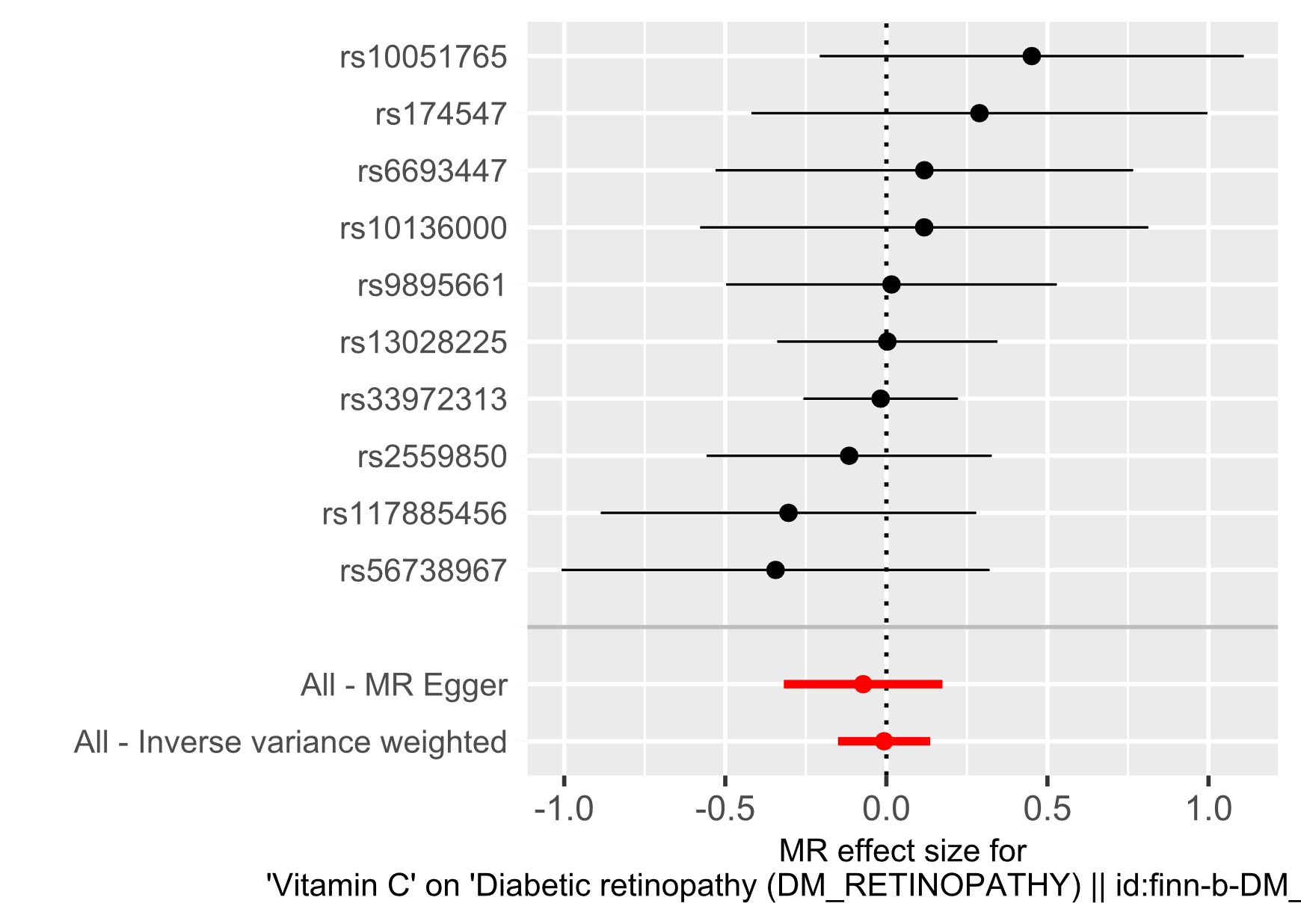


Supplementary material 2: Forest plot of vitamin C for Diabetic complications, such as (A)Diabetic hypoglycemia, (B) Diabetic ketoacidosis, (C) Diabetic maculopathy, (D) Diabetic nephropathy, (E) Diabetic neuropathy and (F) Diabetic retinopathy.
