# Supplementary material for: Causal Relationship Between Various Vitamins and Different Diabetic Complications: A Mendelian Randomization Study
Source: Food Sci Nutr. 2025 Jul 7;13(7):e70536. doi: 10.1002/fsn3.70536 (PMC12230352; doi:10.1002/fsn3.70536)
Supplement: Supplementary file 3 — Appendix S3. Funnel plot of vitamin C for Diabetic complications, such as (A) Diabetic hypoglycemia, (B) Diabetic ketoacidosis, (C) Diabetic maculopathy, (D) Diabetic nephropathy, (E) Diabetic neuropathy, and (F) Diabetic retinopathy. [file FSN3-13-e70536-s010.docx]

(A) Funnel plot of VitC for Diabetic hypoglycemia


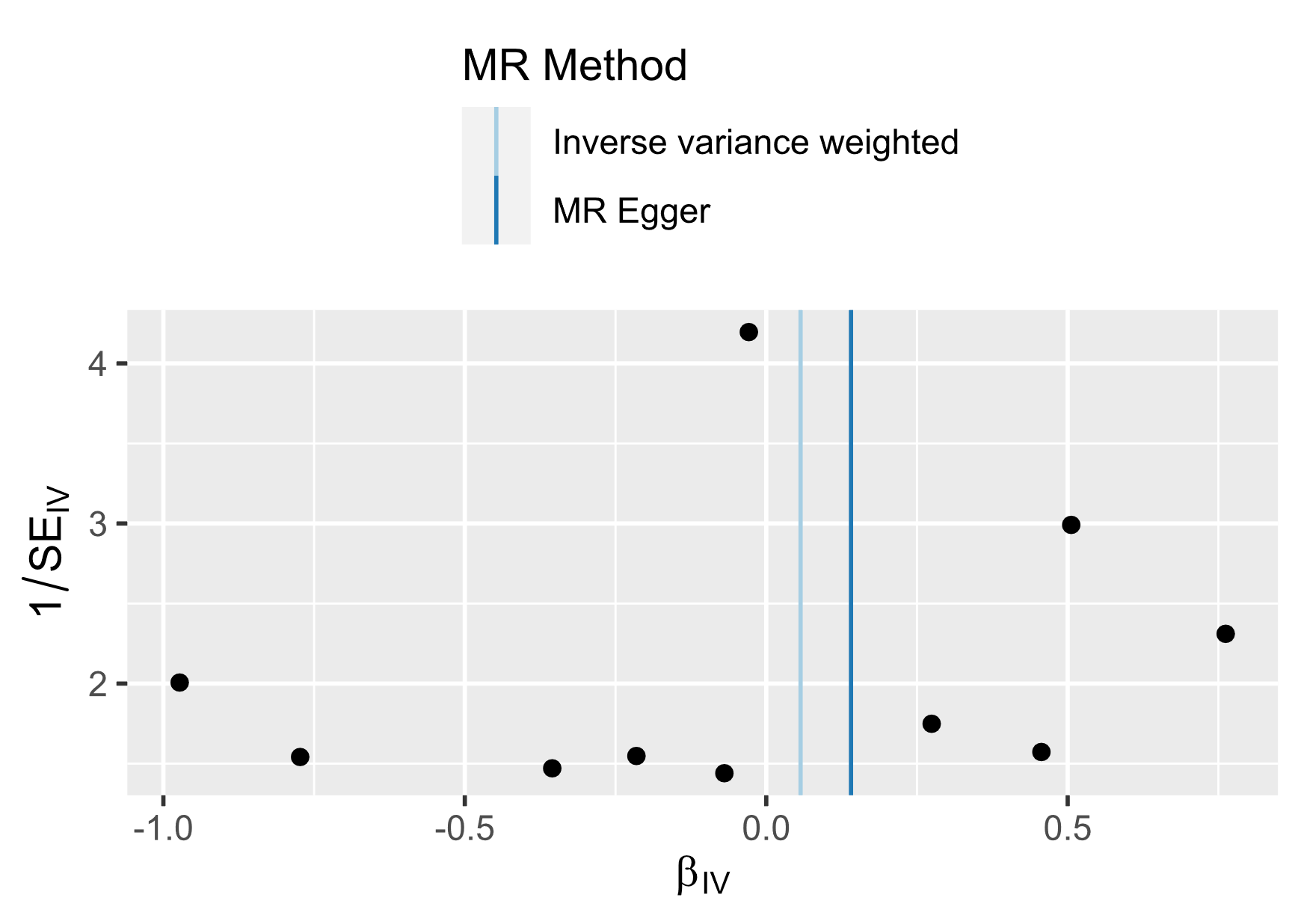


(B) Funnel plot of VitC for Diabetic ketoacidosis


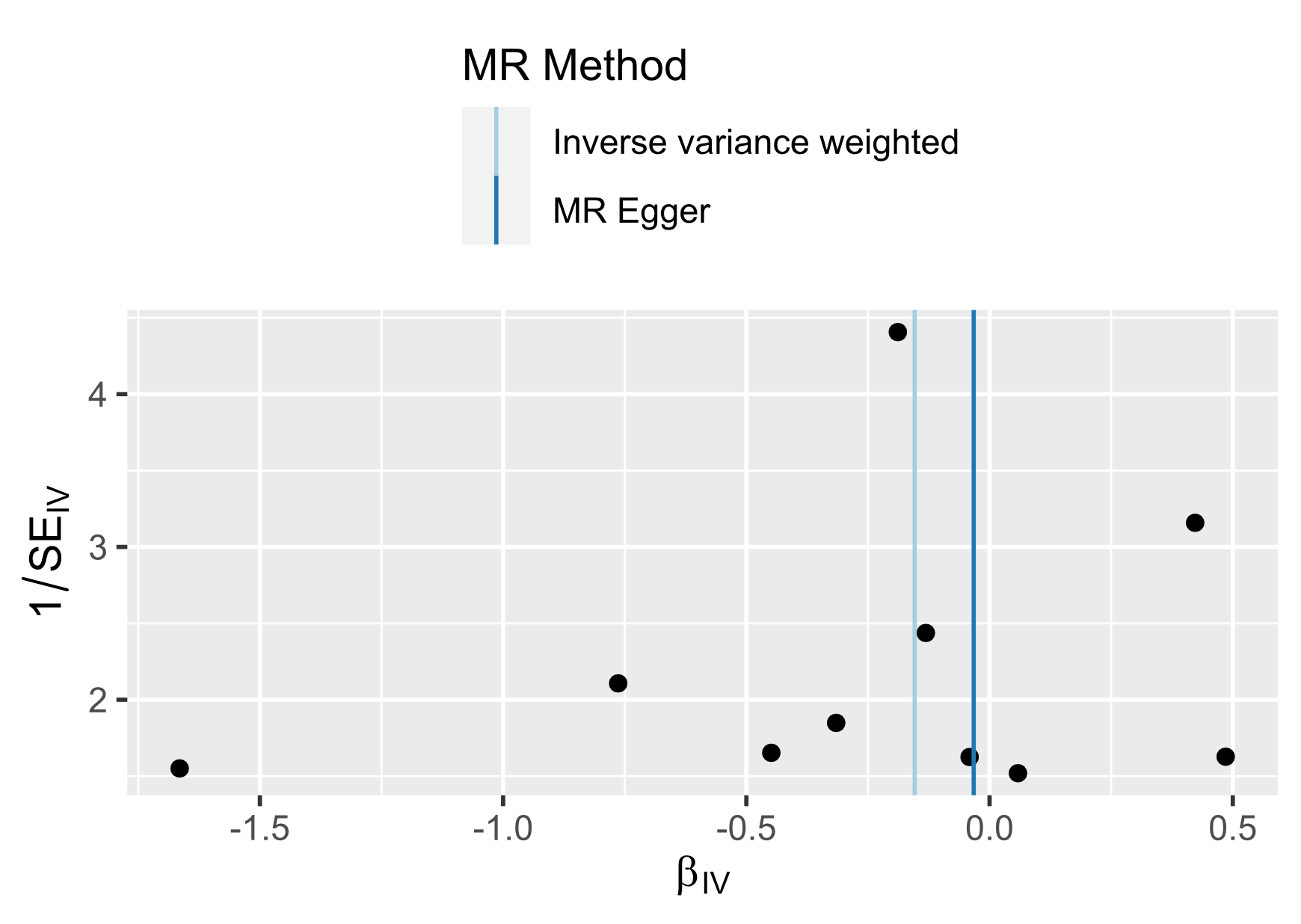


(C) Funnel plot of VitC for Diabetic maculopathy


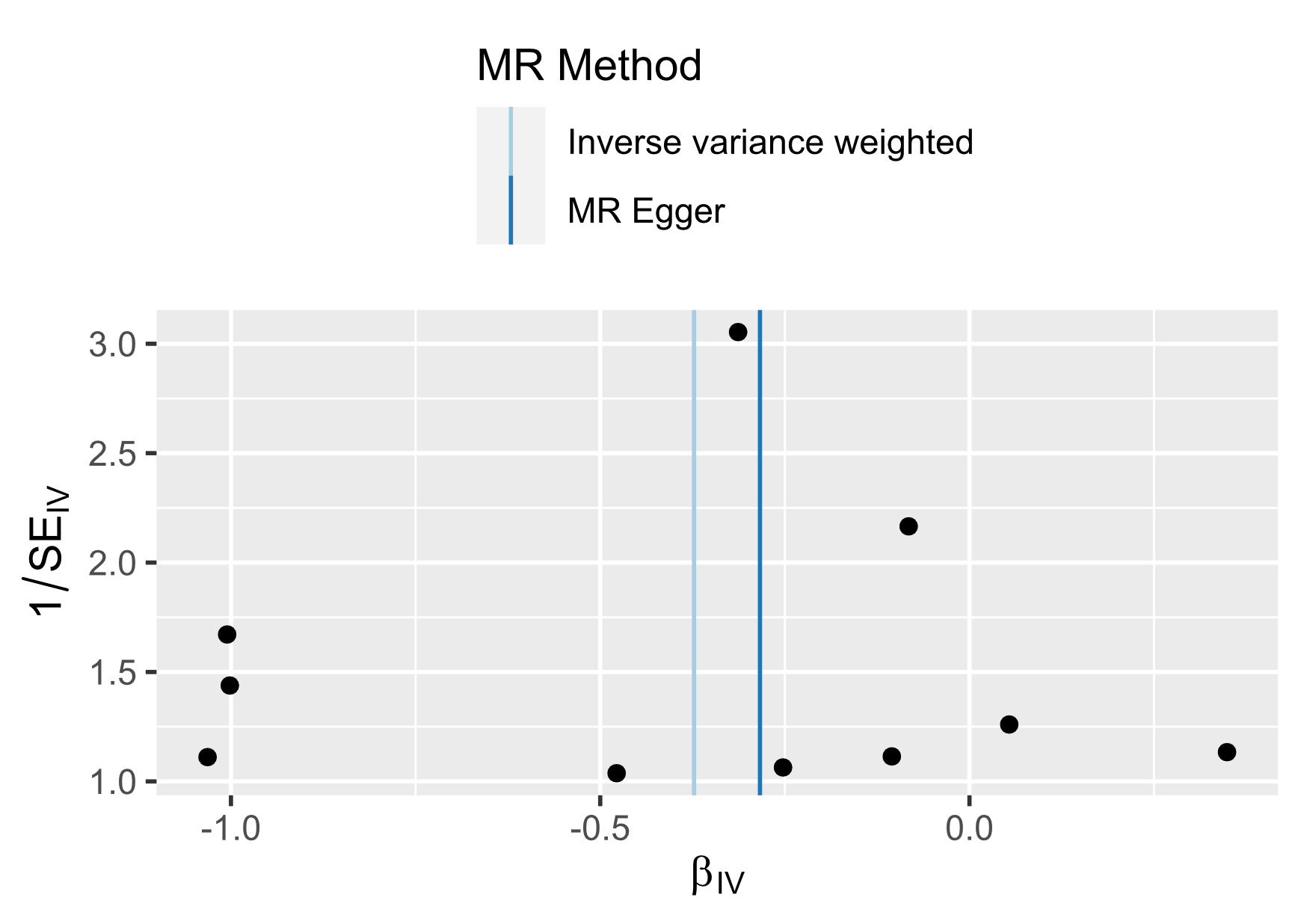


(D) Funnel plot of VitC for Diabetic nephropathy


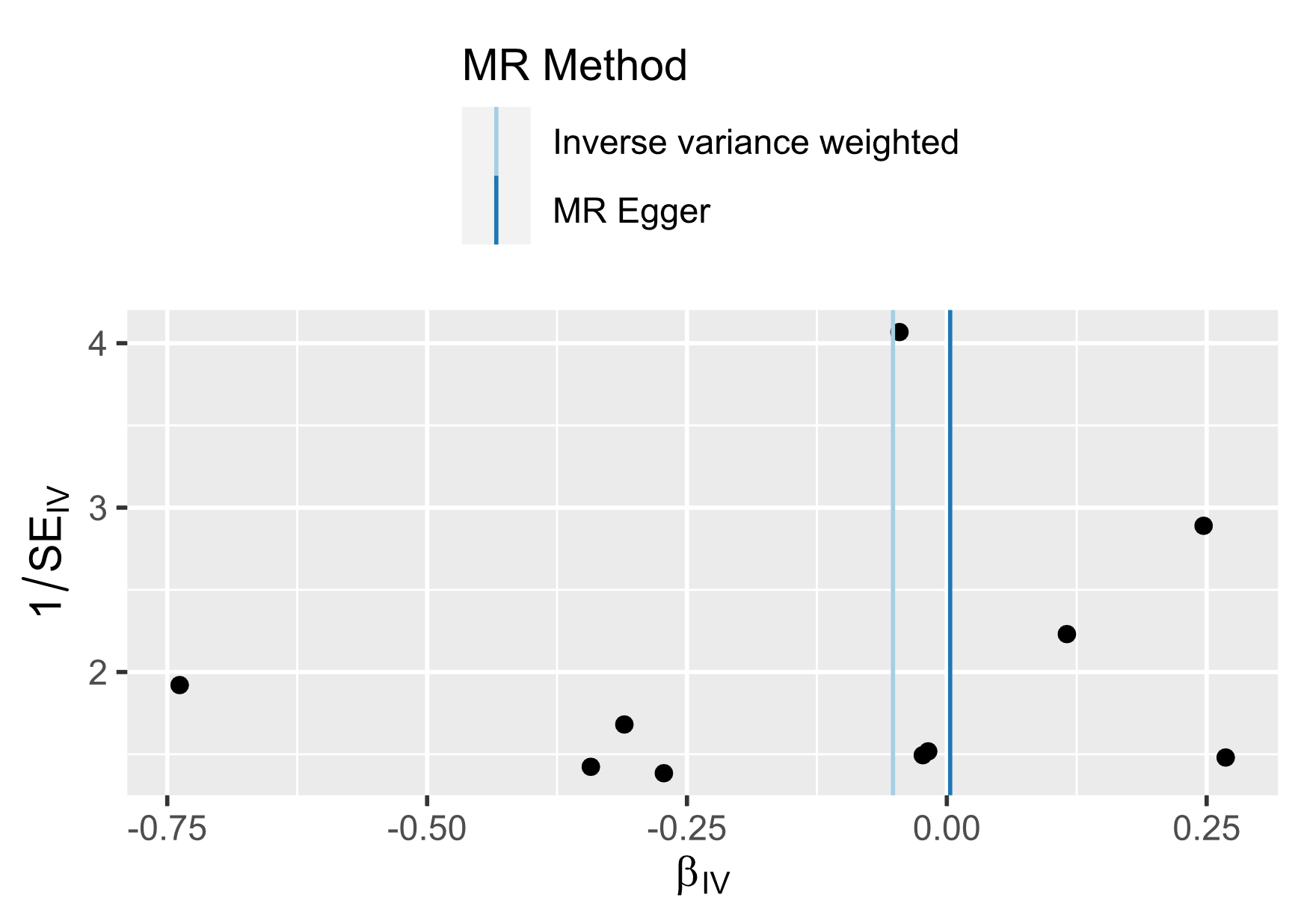


(E) Funnel plot of VitC for Diabetic neuropathy


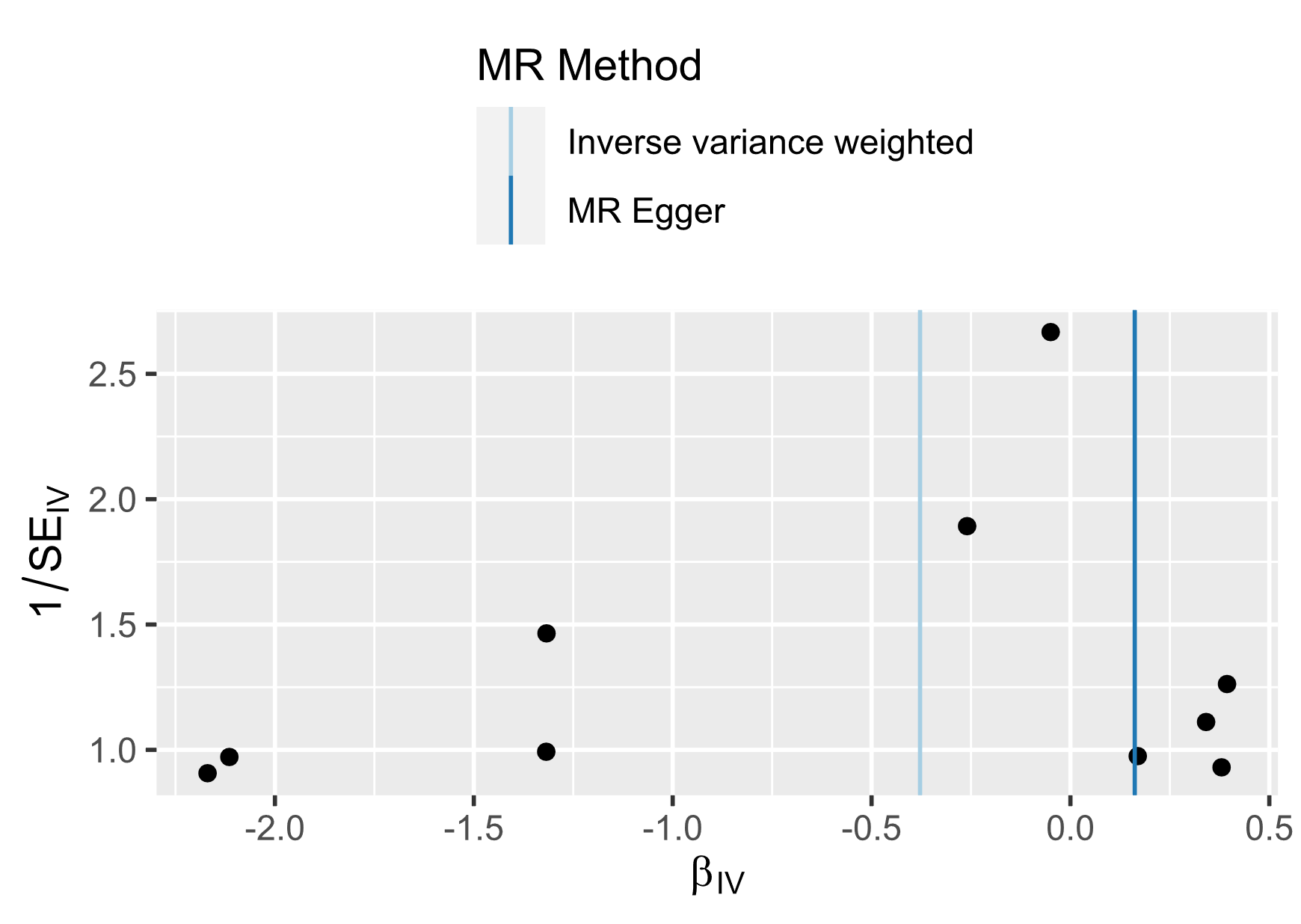


(F) Funnel plot of VitC for Diabetic retinopathy


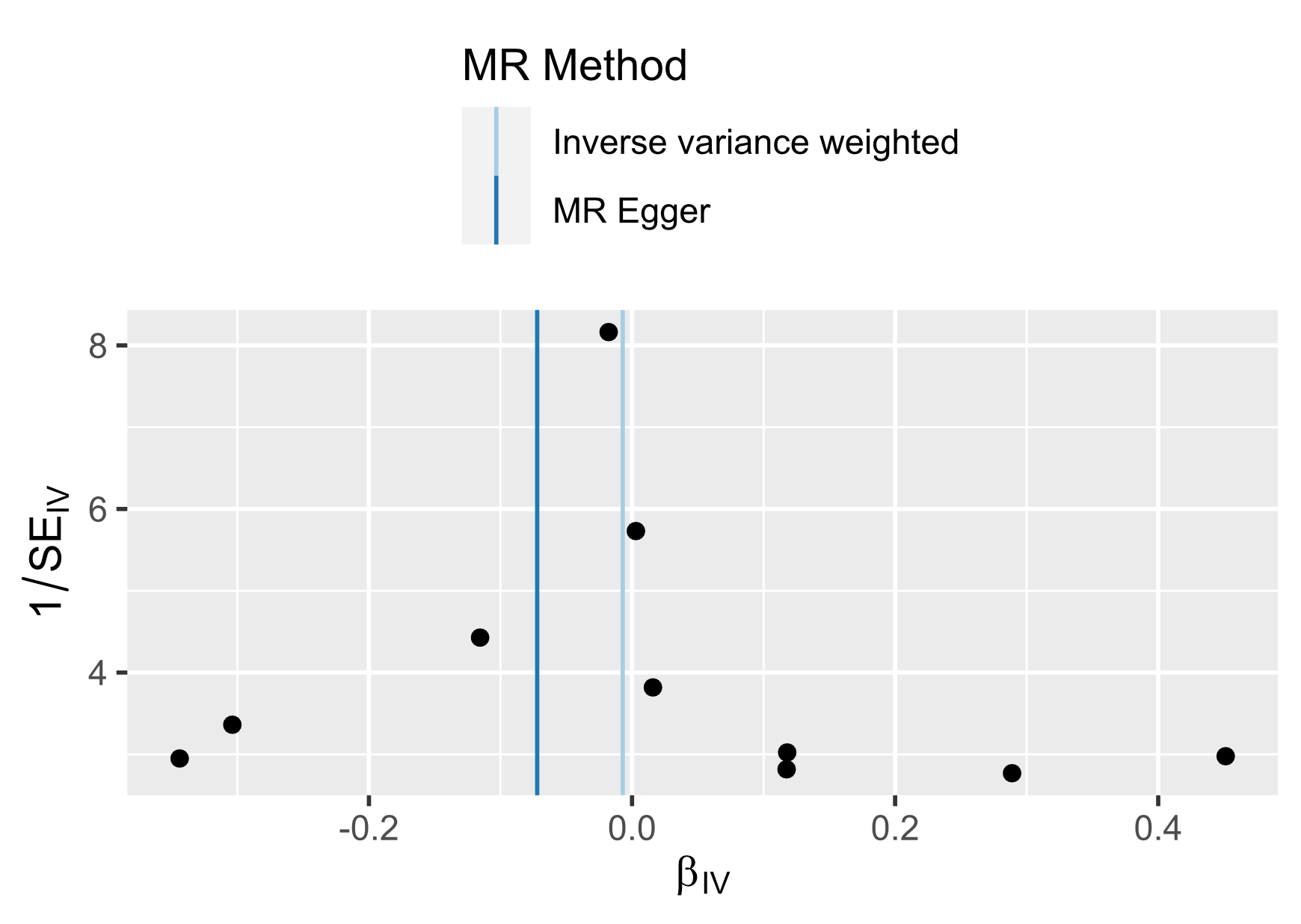


Supplementary material 3: Funnel plot of vitamin C for Diabetic complications, such as (A)Diabetic hypoglycemia, (B) Diabetic ketoacidosis, (C) Diabetic maculopathy, (D) Diabetic nephropathy, (E) Diabetic neuropathy and (F) Diabetic retinopathy.
