# Supplementary material for: Causal Relationship Between Various Vitamins and Different Diabetic Complications: A Mendelian Randomization Study
Source: Food Sci Nutr. 2025 Jul 7;13(7):e70536. doi: 10.1002/fsn3.70536 (PMC12230352; doi:10.1002/fsn3.70536)
Supplement: Supplementary file 4 — Appendix S4. Leave−one−out sensitivity analysis for Vitamin C on Diabetic complications, such as (A) Diabetic hypoglycemia, (B) Diabetic ketoacidosis, (C) Diabetic maculopathy, (D) Diabetic nephropathy, (E) Diabetic neuropathy and (F) Diabetic retinopathy. [file FSN3-13-e70536-s009.docx]

(A) Leave−one−out sensitivity analysis for VitC on Diabetic hypoglycemia.


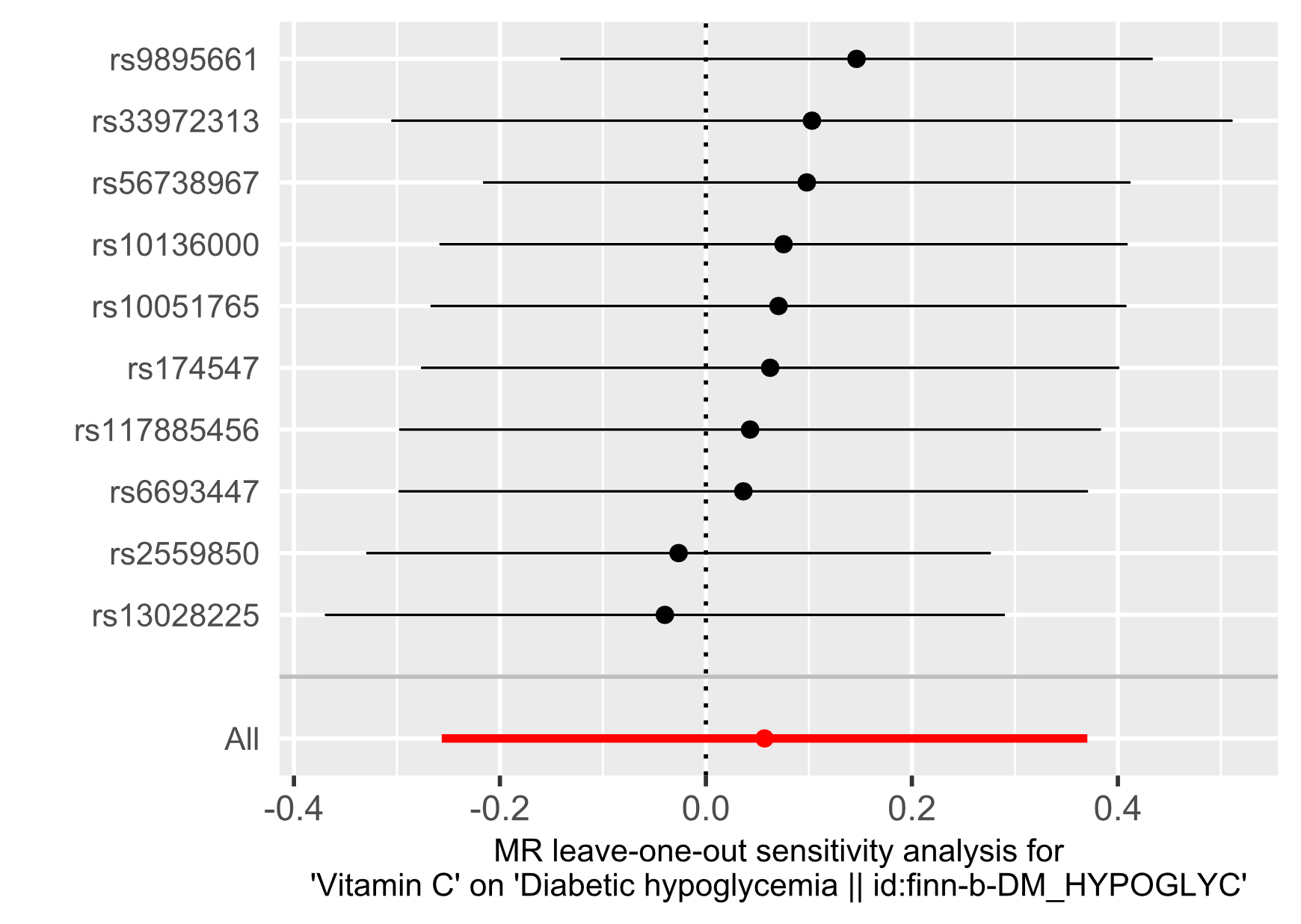


(B) Leave−one−out sensitivity analysis for VitC on Diabetic ketoacidosis.


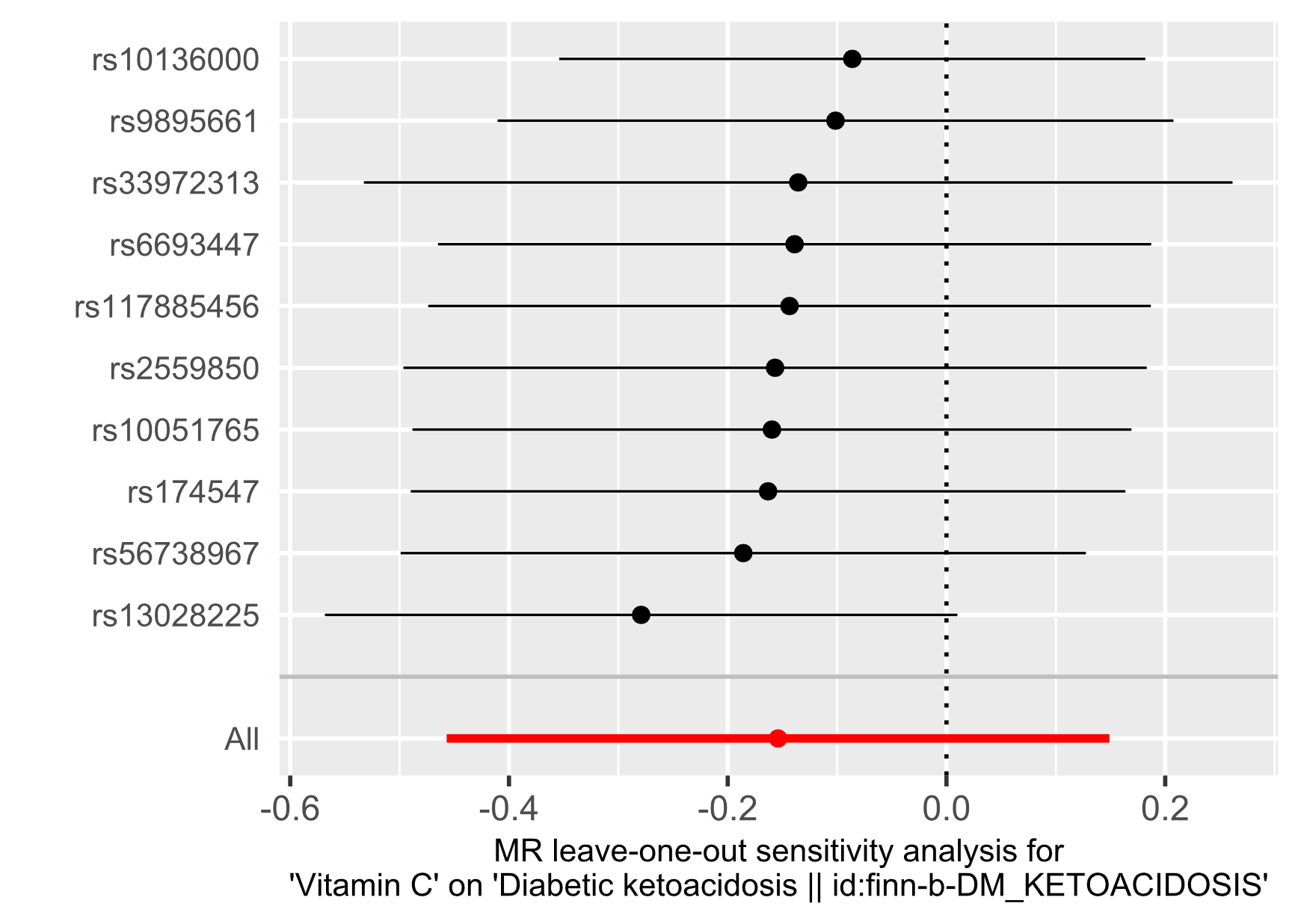


(C) Leave−one−out sensitivity analysis for VitC on Diabetic maculopathy.


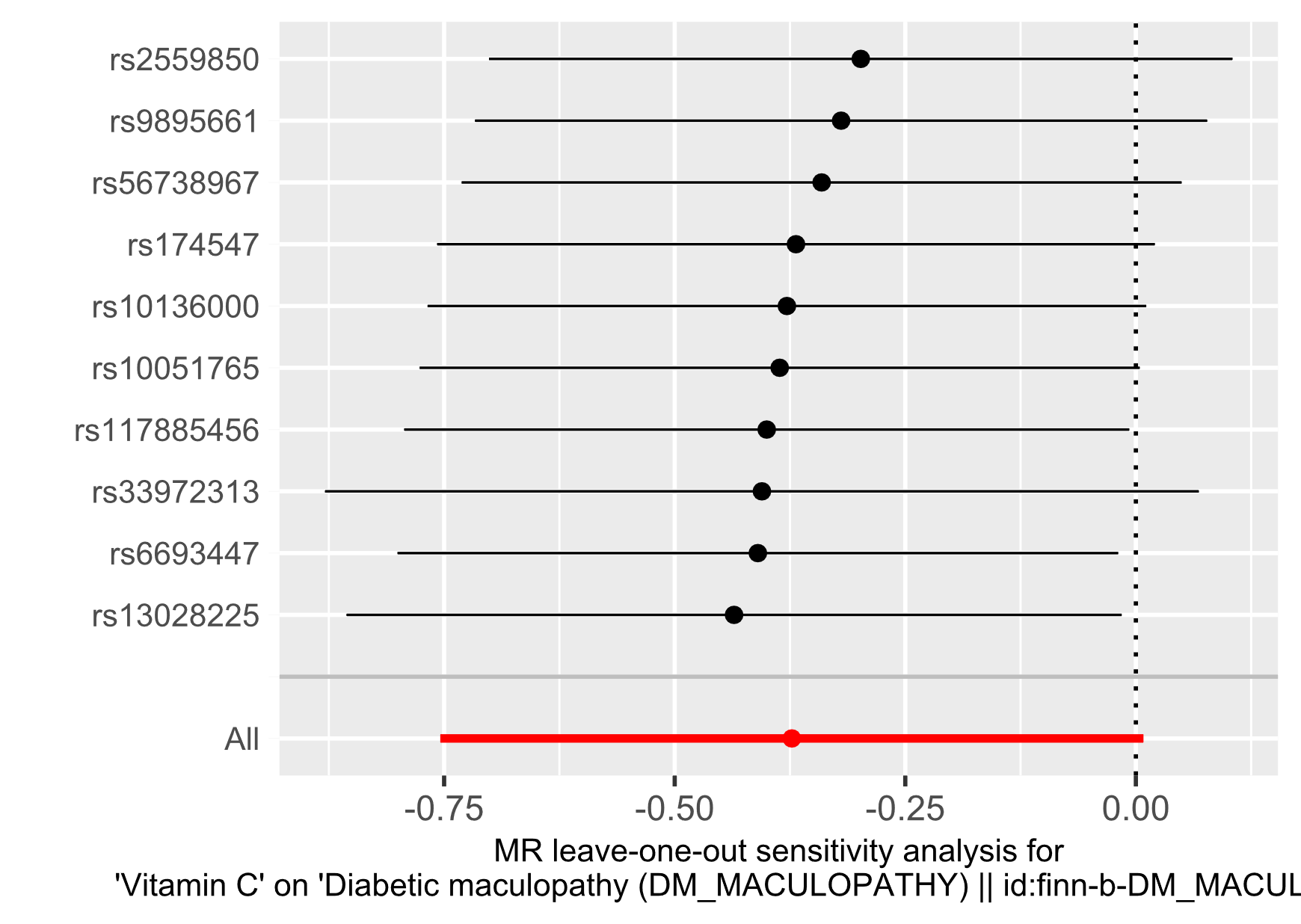


(D) Leave−one−out sensitivity analysis for VitC on Diabetic nephropathy.


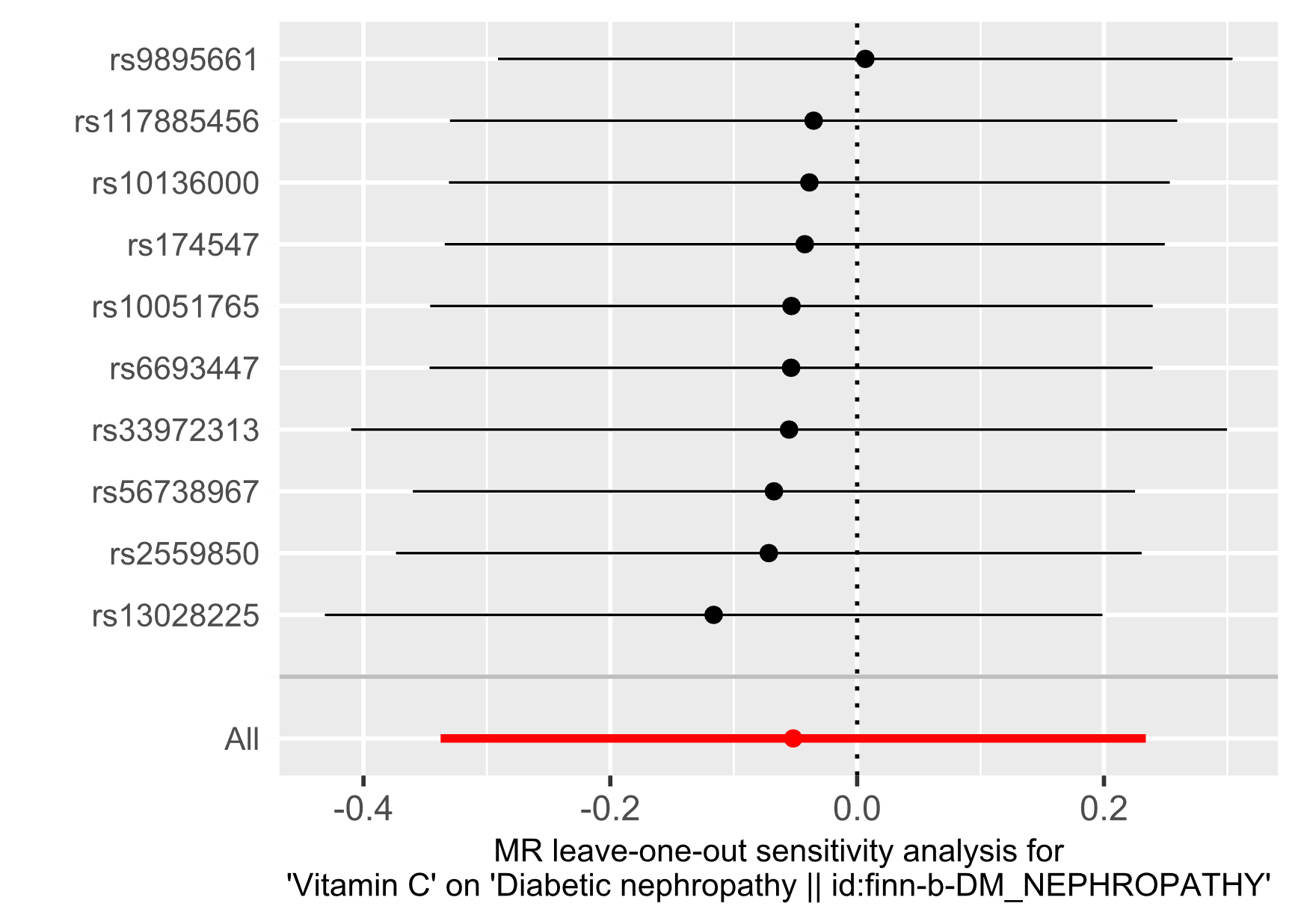


(E) Leave−one−out sensitivity analysis for VitC on Diabetic neuropathy.


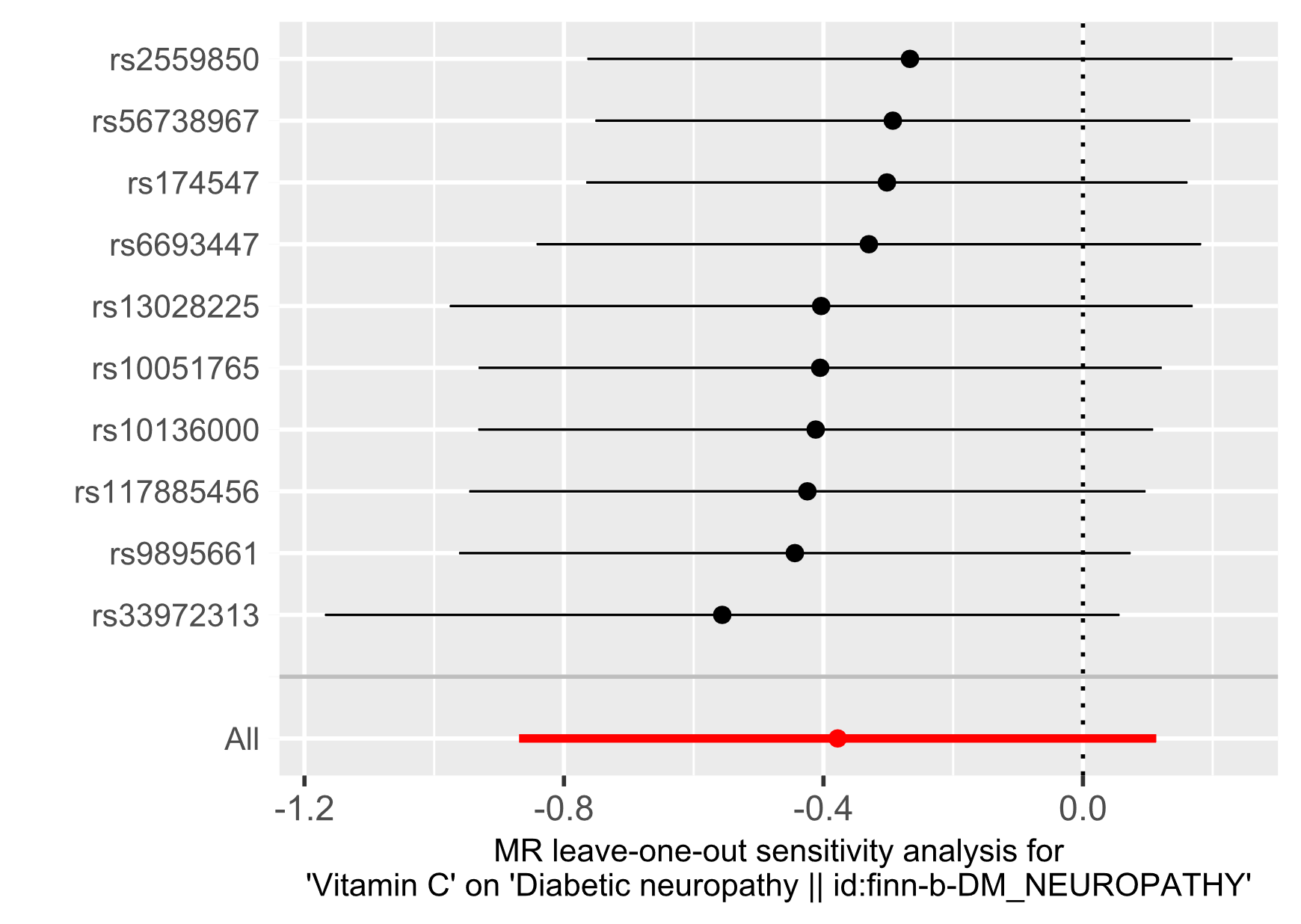


(F) Leave−one−out sensitivity analysis for VitC on Diabetic retinopathy.


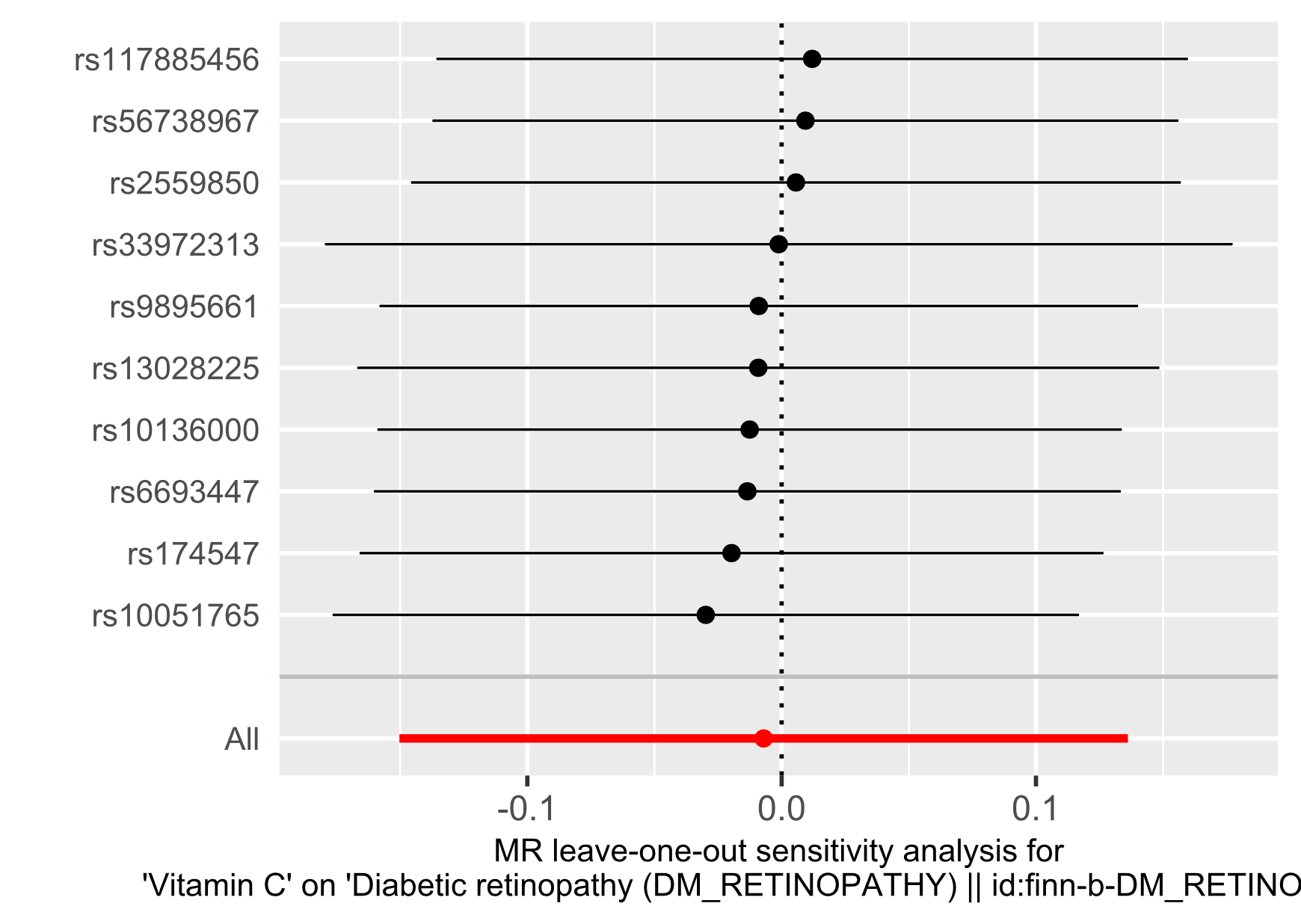


Supplementary material 4: Leave−one−out sensitivity analysis for Vitamin C on Diabetic complications, such as (A) Diabetic hypoglycemia, (B) Diabetic ketoacidosis, (C) Diabetic maculopathy, (D) Diabetic nephropathy, (E) Diabetic neuropathy and (F) Diabetic retinopathy.
