# Supplementary material for: Causal Relationship Between Various Vitamins and Different Diabetic Complications: A Mendelian Randomization Study
Source: Food Sci Nutr. 2025 Jul 7;13(7):e70536. doi: 10.1002/fsn3.70536 (PMC12230352; doi:10.1002/fsn3.70536)
Supplement: Supplementary file 5 — Appendix S5. Scatter plot of vitamin C for Diabetic complications, such as (A) Diabetic hypoglycemia, (B) Diabetic ketoacidosis, (C) Diabetic maculopathy, (D) Diabetic nephropathy, (E) Diabetic neuropathy and (F) Diabetic retinopathy. [file FSN3-13-e70536-s011.docx]

(A) Scatter plot of VitC on Diabetic hypoglycemia.


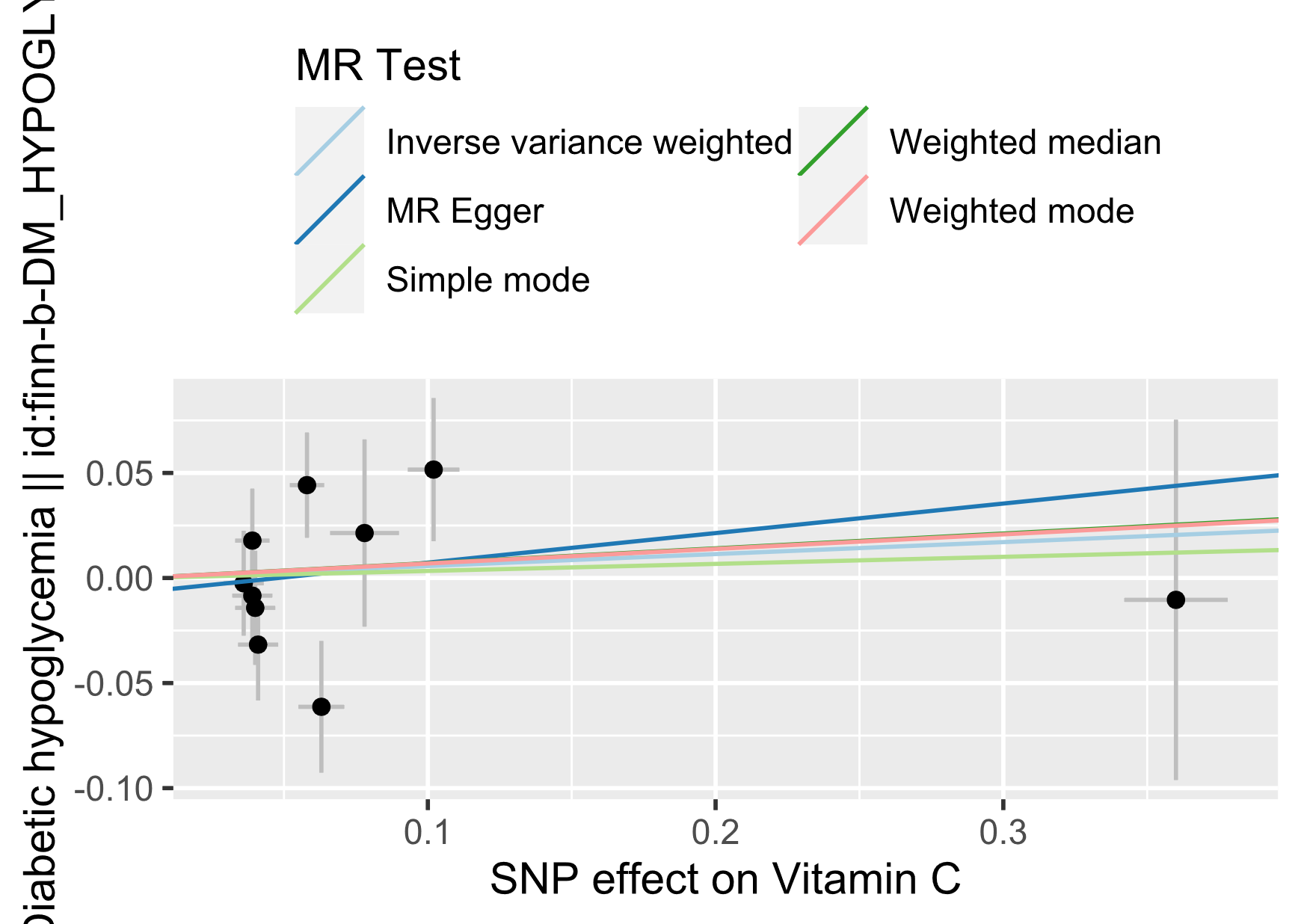


(B) Scatter plot of VitC on Diabetic ketoacidosis.


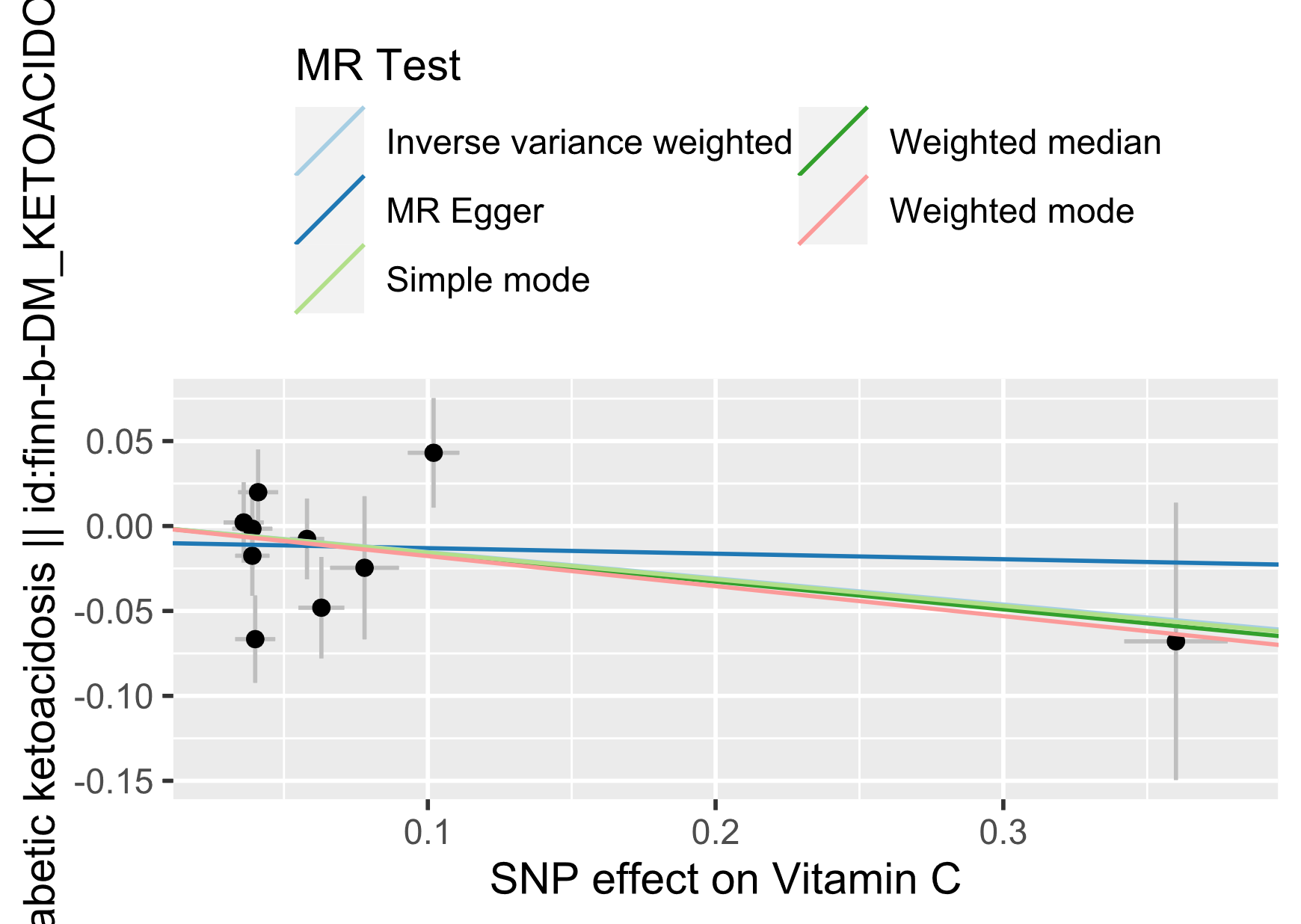


(C) Scatter plot of VitC on Diabetic maculopathy.


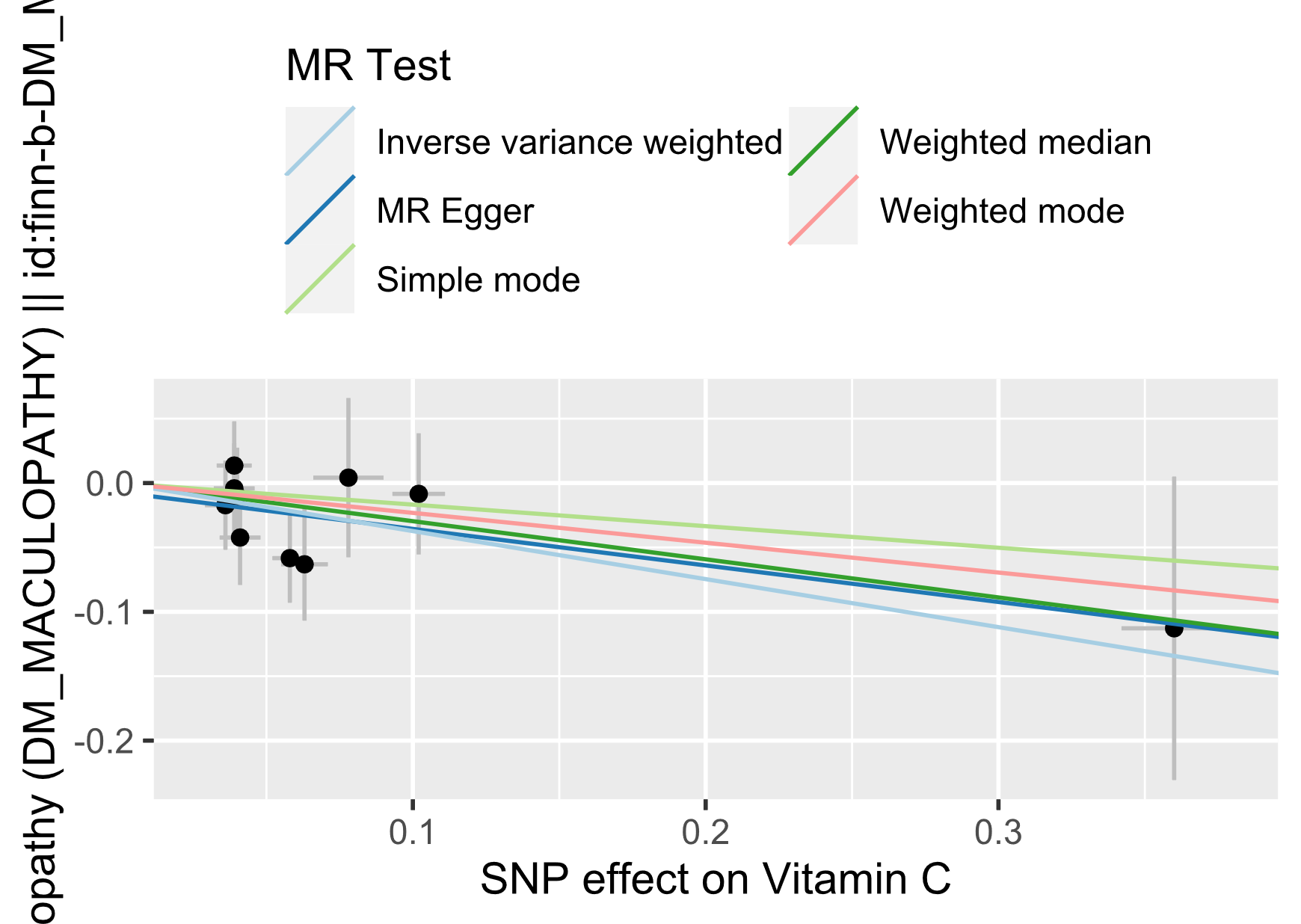


(D) Scatter plot of VitC on Diabetic nephropathy.


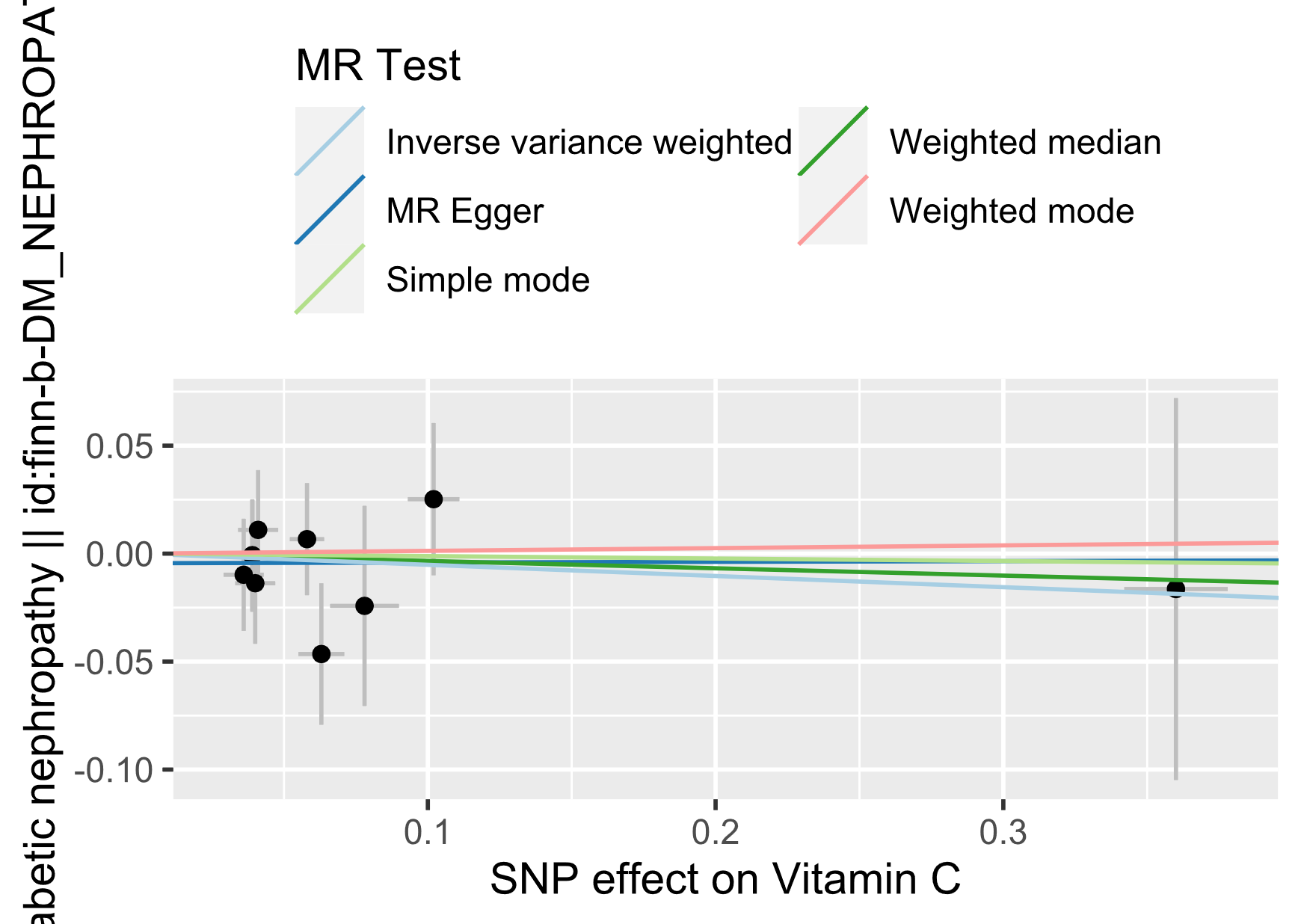


(E) Scatter plot of VitC on Diabetic neuropathy.


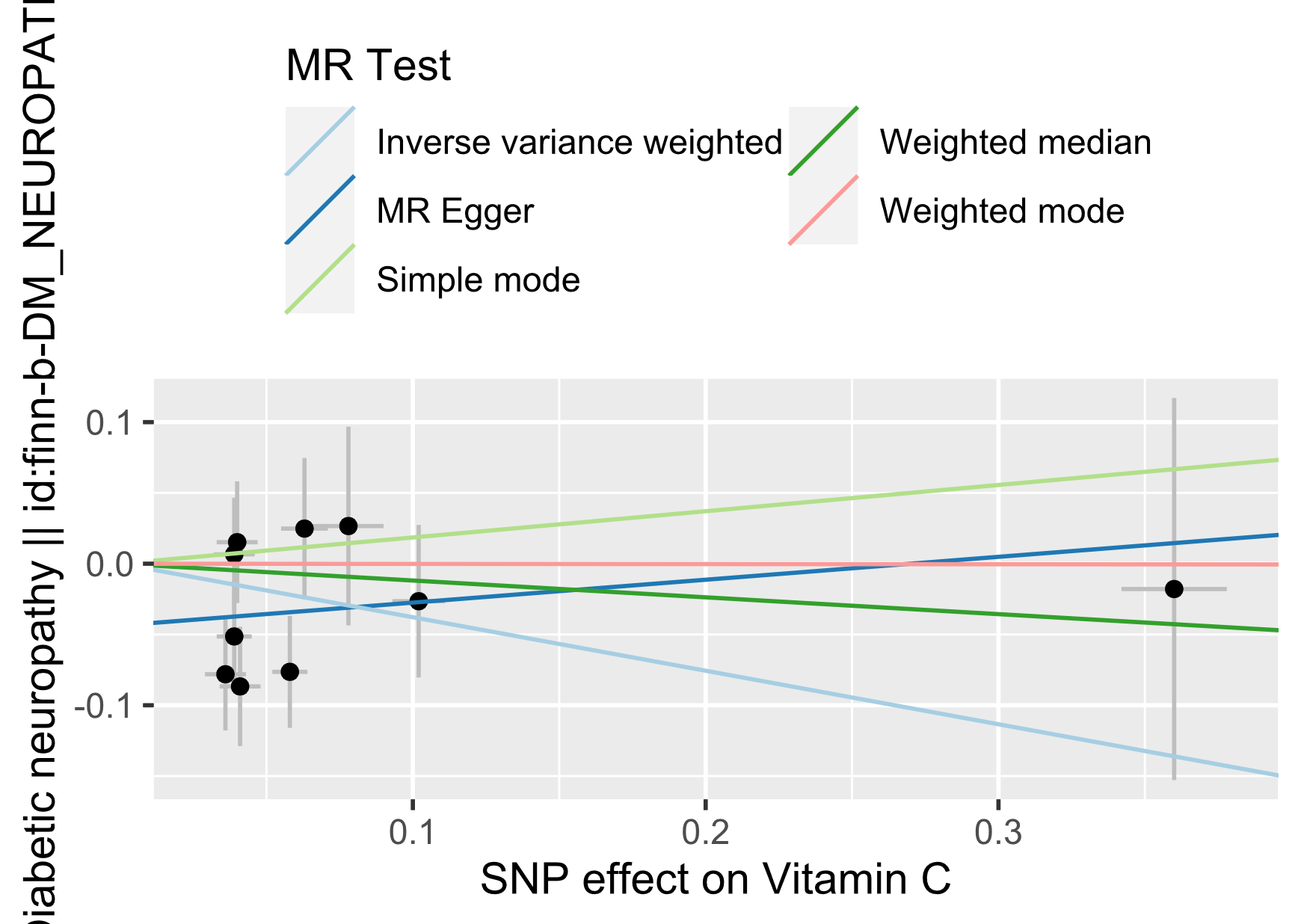


(F) Scatter plot of VitC on Diabetic retinopathy.


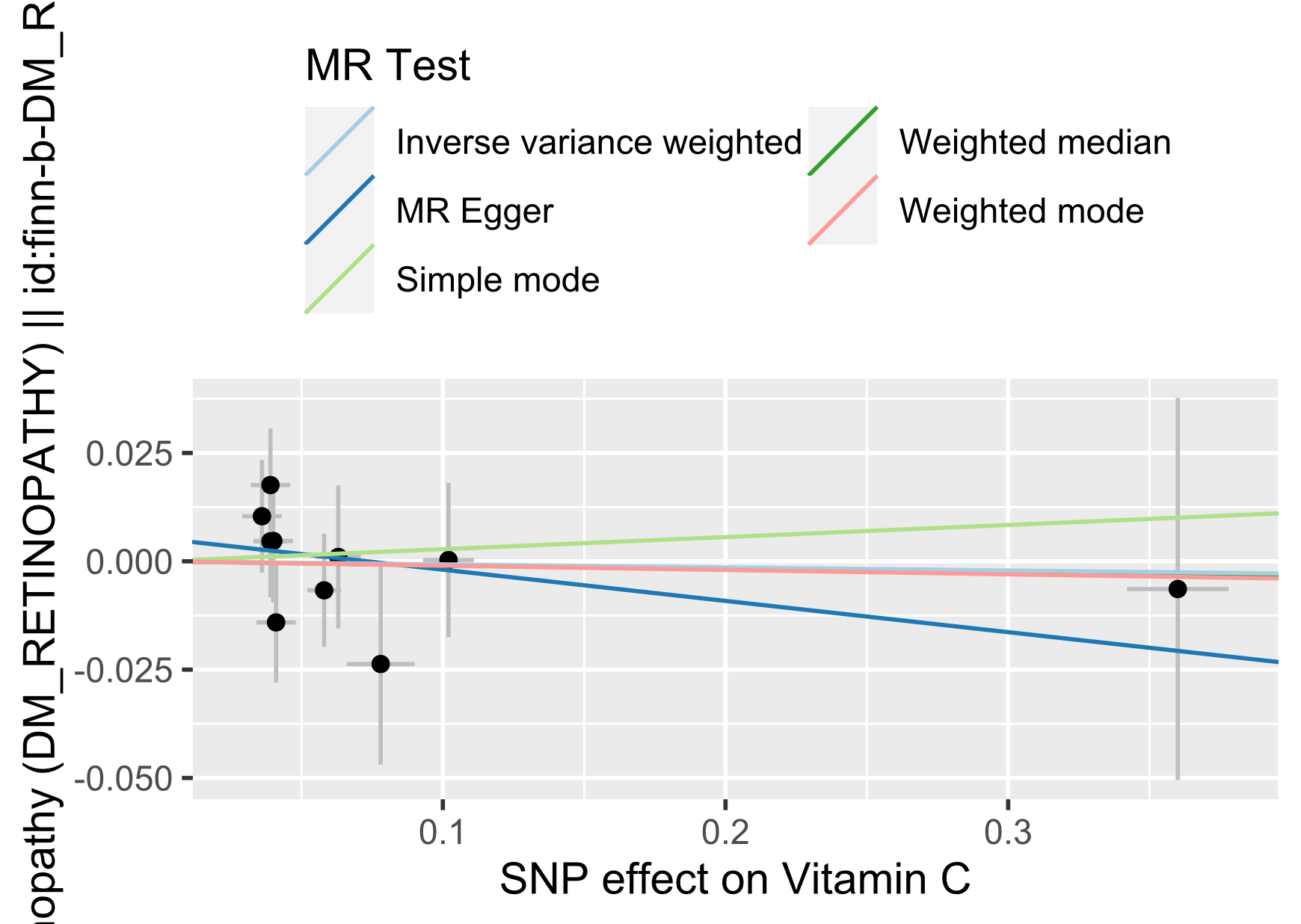


Supplementary material 5: Scatter plot of vitamin C for Diabetic complications, such as (A) Diabetic hypoglycemia, (B) Diabetic ketoacidosis, (C) Diabetic maculopathy, (D) Diabetic nephropathy, (E) Diabetic neuropathy and (F) Diabetic retinopathy.
