# Supplementary material for: Causal Relationship Between Various Vitamins and Different Diabetic Complications: A Mendelian Randomization Study
Source: Food Sci Nutr. 2025 Jul 7;13(7):e70536. doi: 10.1002/fsn3.70536 (PMC12230352; doi:10.1002/fsn3.70536)
Supplement: Supplementary file 6 — Appendix S6. Forest plot of vitamin D for Diabetic complications, such as (A) Diabetic hypoglycemia, (B) Diabetic ketoacidosis, (C) Diabetic maculopathy, (D) Diabetic nephropathy, (E) Diabetic neuropathy, and (F) Diabetic retinopathy. [file FSN3-13-e70536-s002.docx]

(A) Forest plot of VitD for Diabetic hypoglycemia


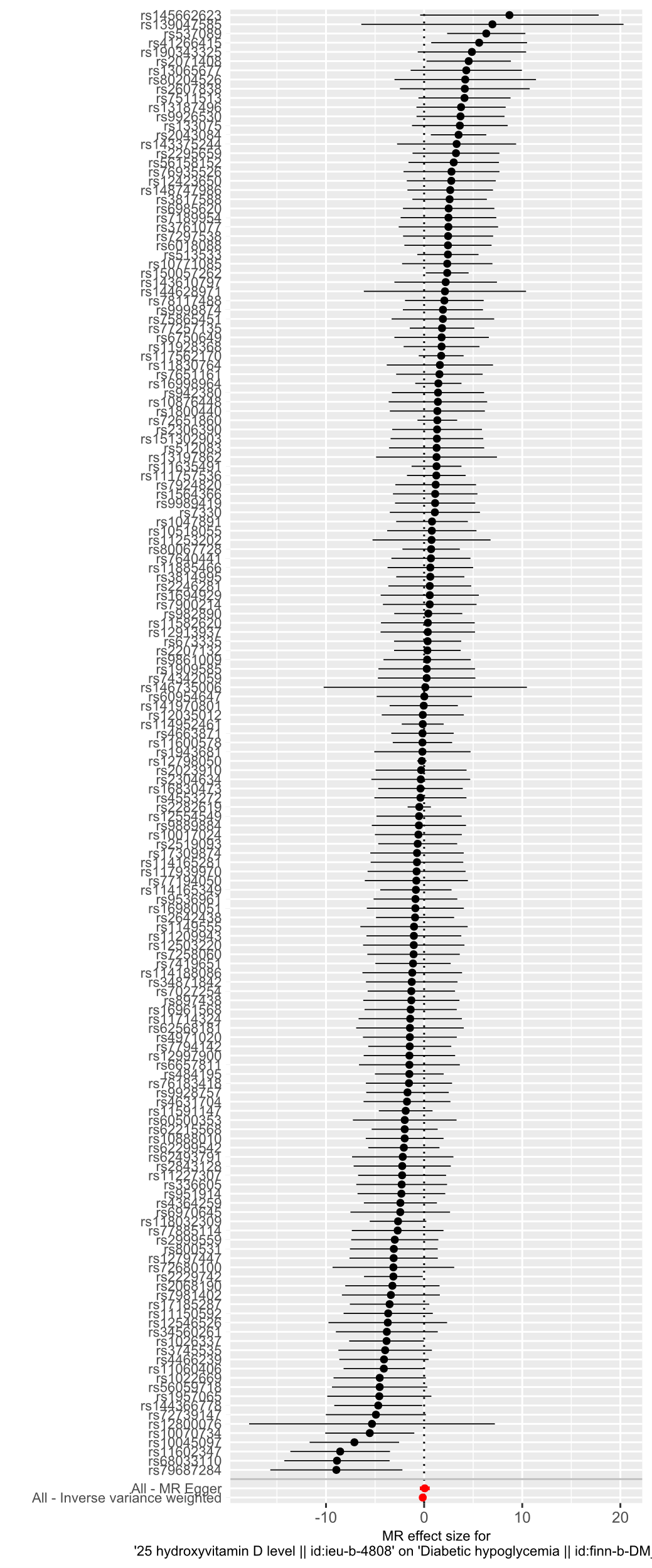


(B) Forest plot of VitD for Diabetic ketoacidosis


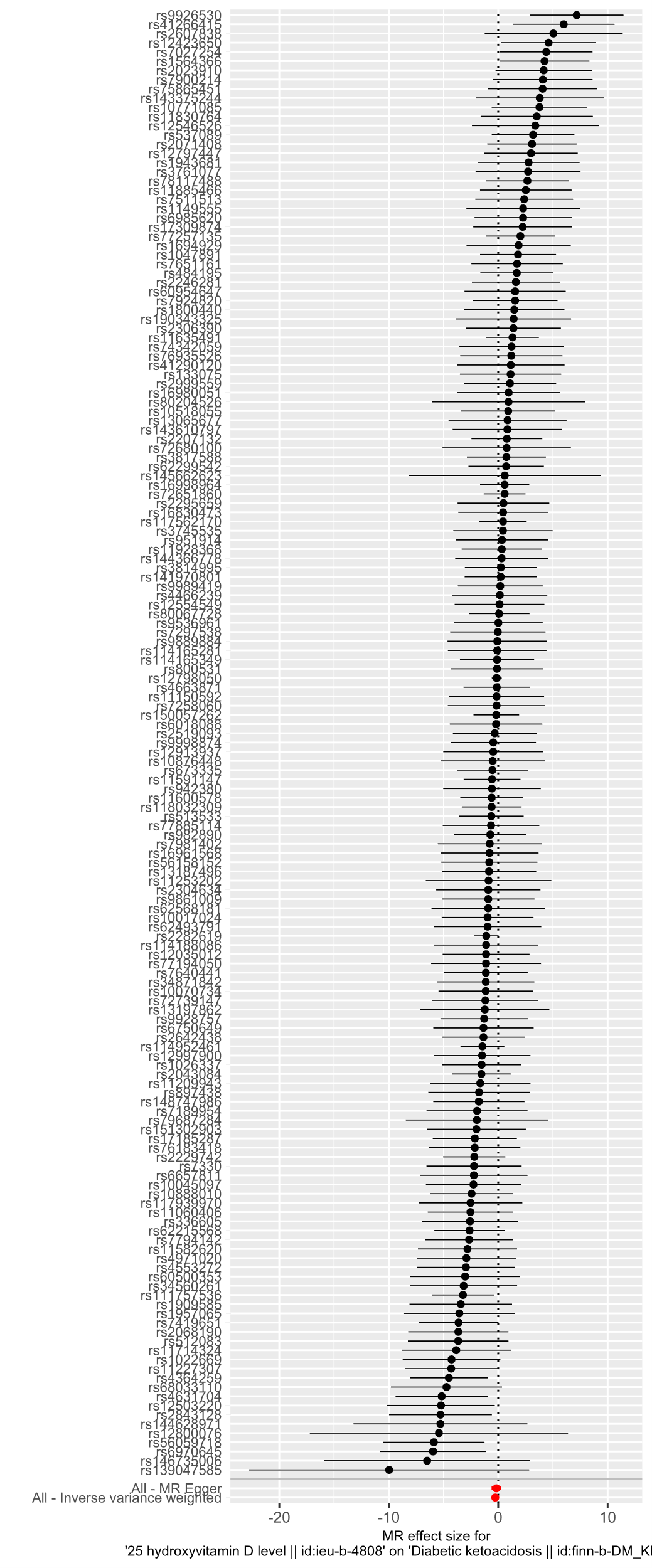


(C) Forest plot of VitD for Diabetic maculopathy


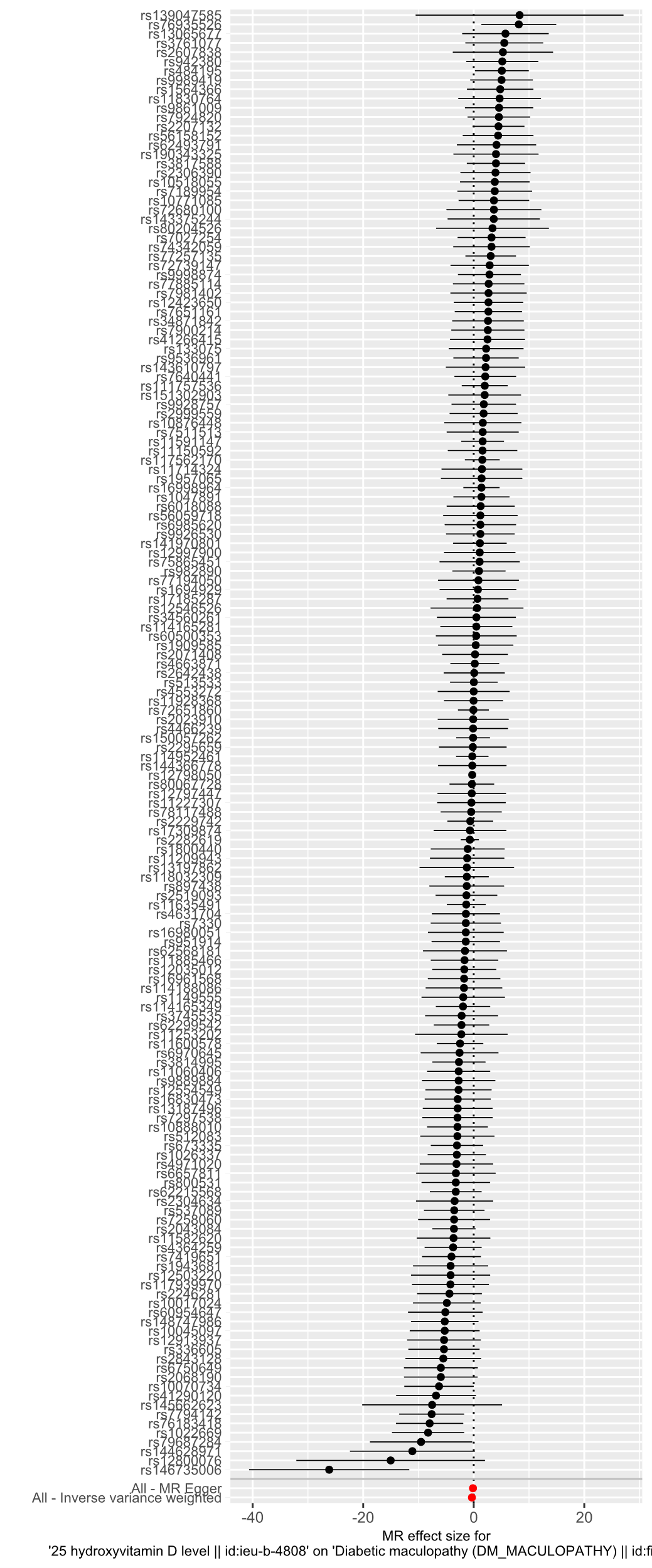


(D) Forest plot of VitD for Diabetic nephropathy


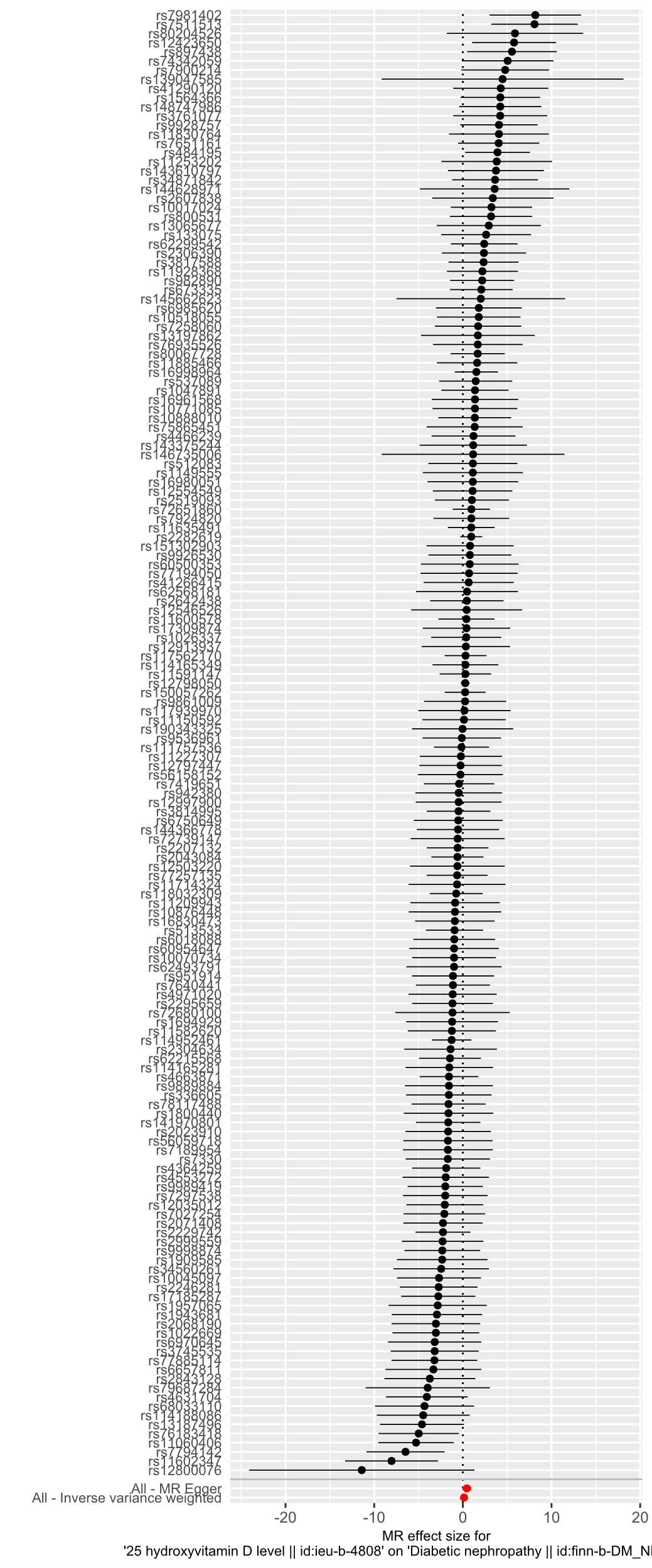


(E) Forest plot of VitD for Diabetic neuropathy


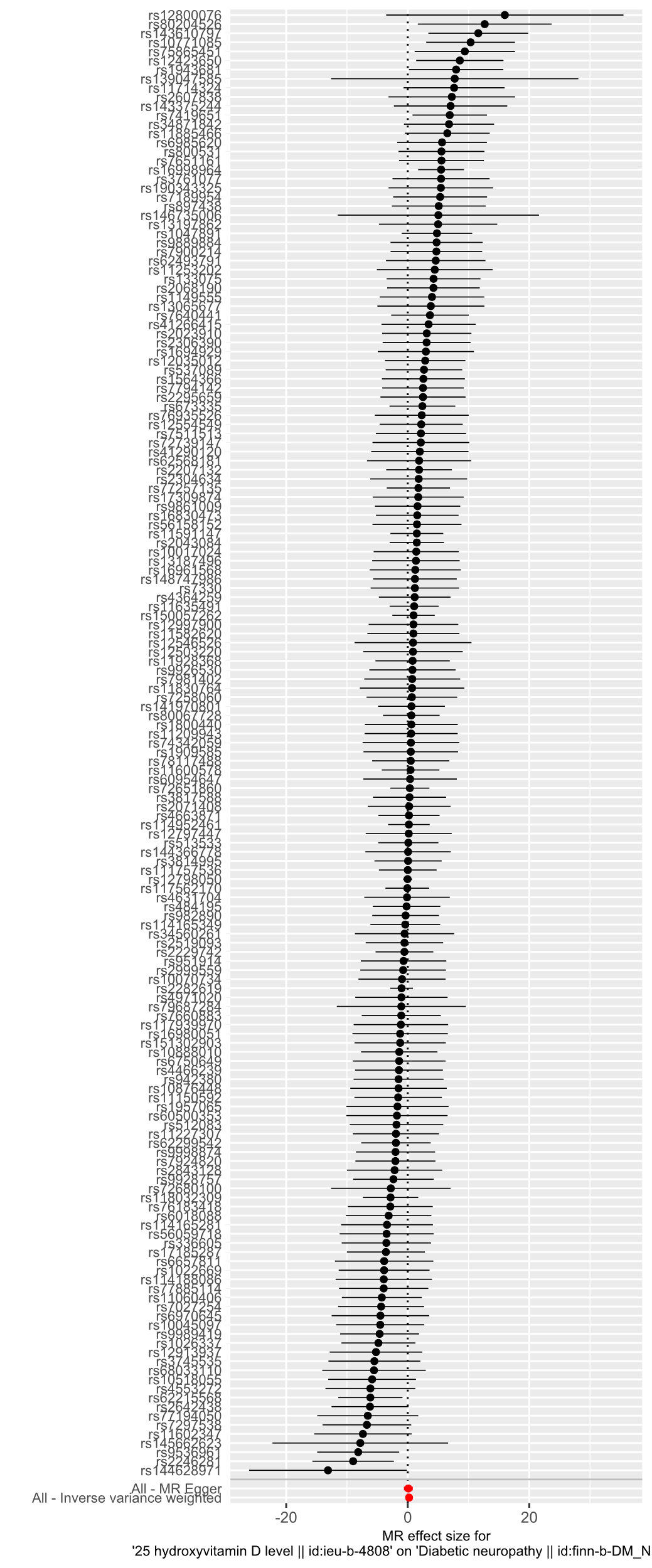


(F) Forest plot of VitD for Diabetic retinopathy


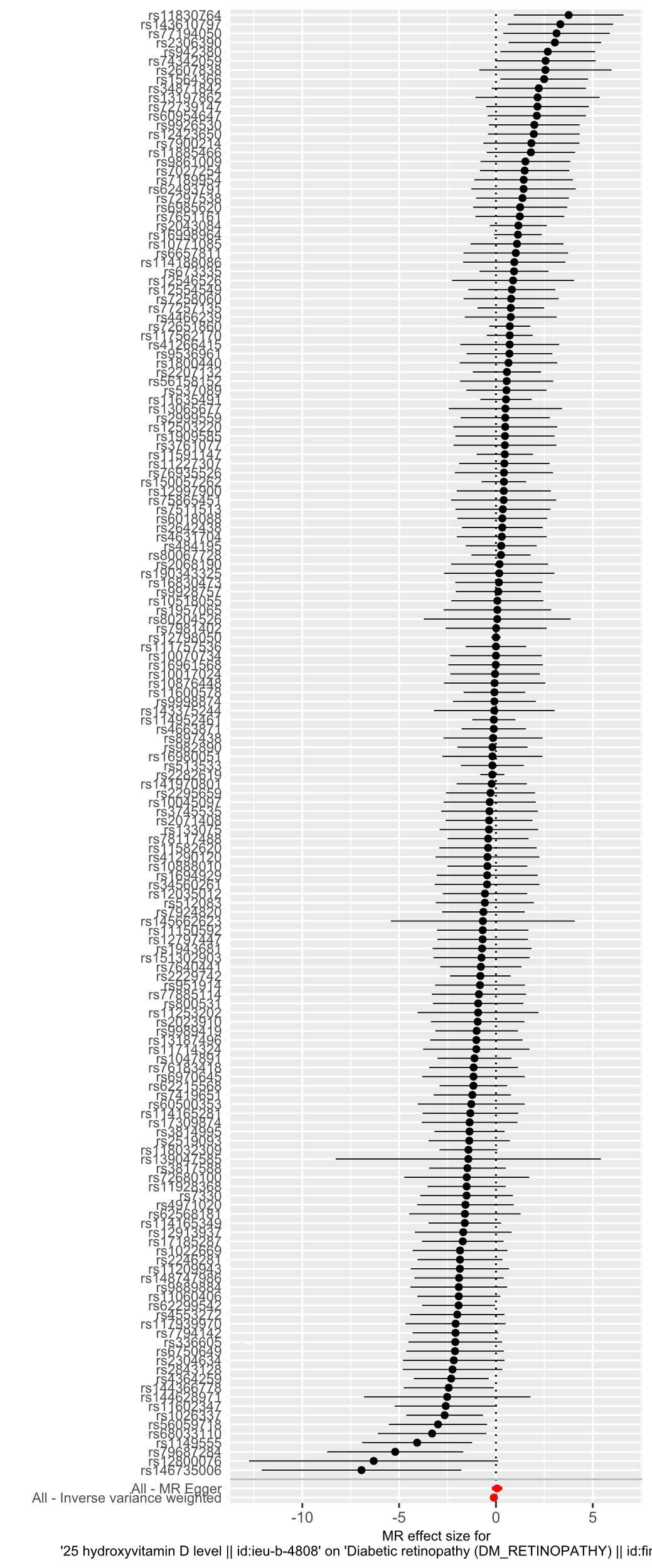


Supplementary material 6: Forest plot of vitamin D for Diabetic complications, such as (A)Diabetic hypoglycemia, (B) Diabetic ketoacidosis, (C) Diabetic maculopathy, (D) Diabetic nephropathy, (E) Diabetic neuropathy and (F) Diabetic retinopathy.
