# Supplementary material for: Causal Relationship Between Various Vitamins and Different Diabetic Complications: A Mendelian Randomization Study
Source: Food Sci Nutr. 2025 Jul 7;13(7):e70536. doi: 10.1002/fsn3.70536 (PMC12230352; doi:10.1002/fsn3.70536)
Supplement: Supplementary file 7 — Appendix S7. Funnel plot of vitamin D for Diabetic complications, such as (A) Diabetic hypoglycemia, (B) Diabetic ketoacidosis, (C) Diabetic maculopathy, (D) Diabetic nephropathy, (E) Diabetic neuropathy, and (F) Diabetic retinopathy. [file FSN3-13-e70536-s006.docx]

(A) Funnel plot of VitD for Diabetic hypoglycemia


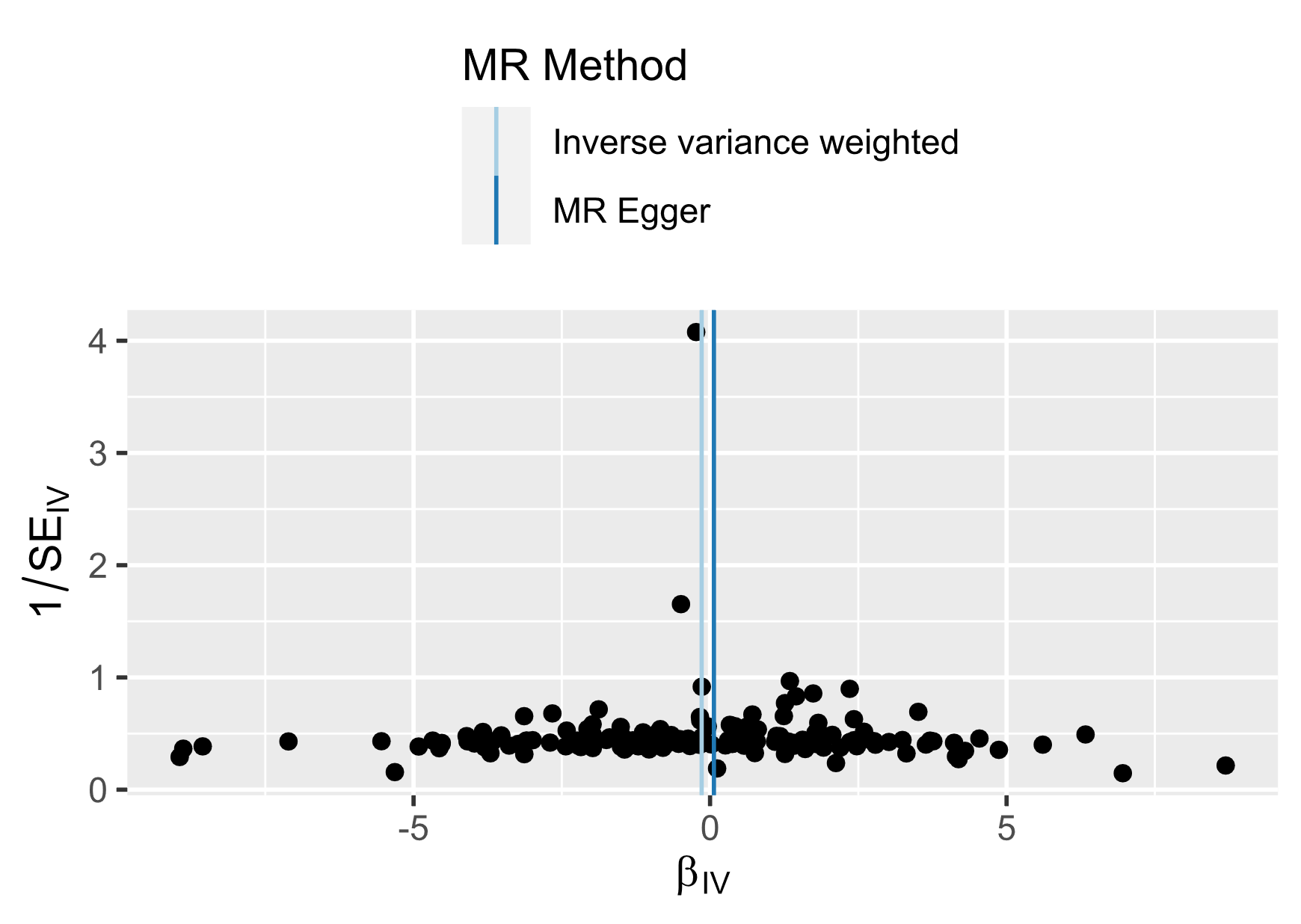


(B) Funnel plot of VitD for Diabetic ketoacidosis


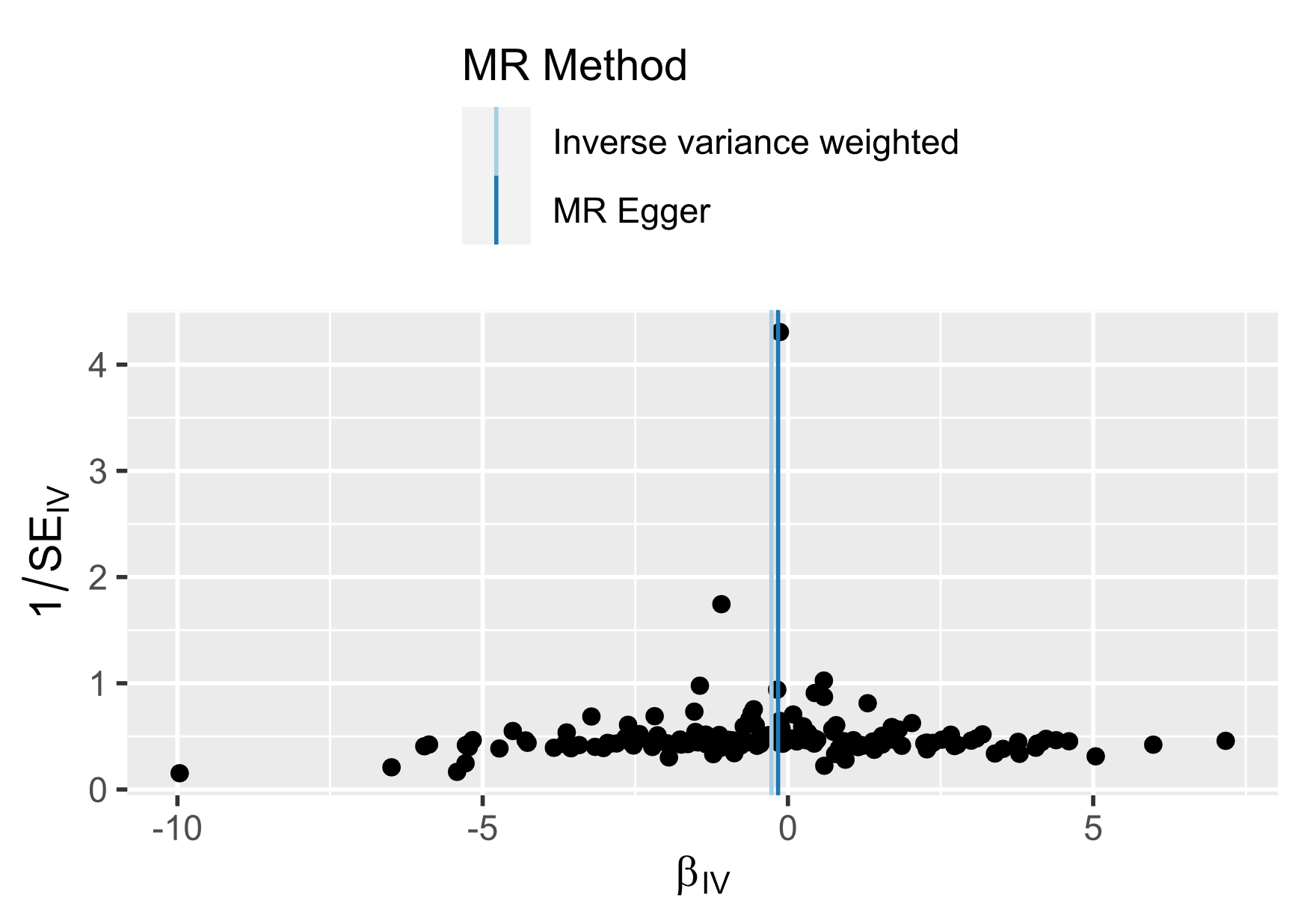


(C) Funnel plot of VitD for Diabetic maculopathy


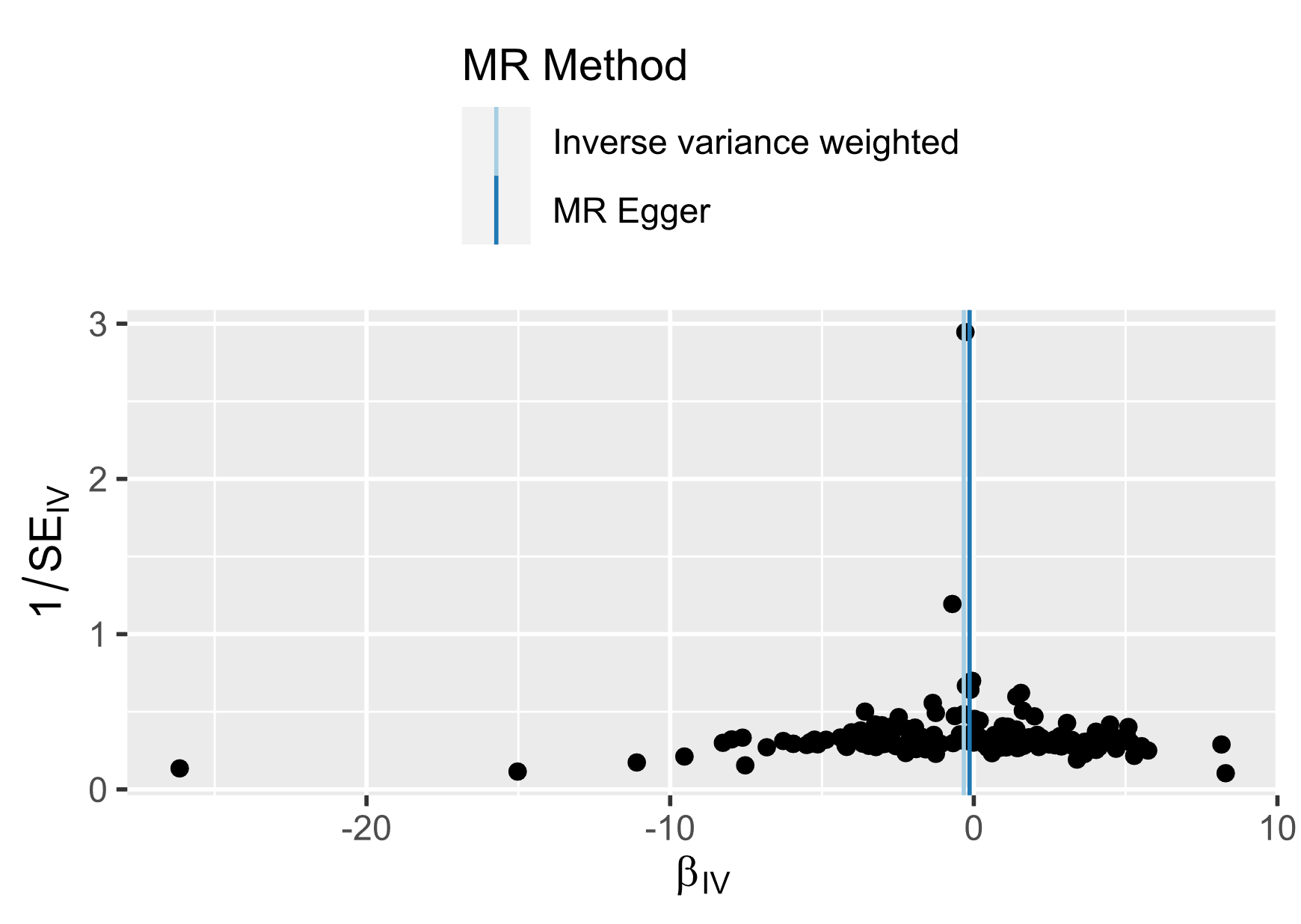


(D) Funnel plot of VitD for Diabetic nephropathy


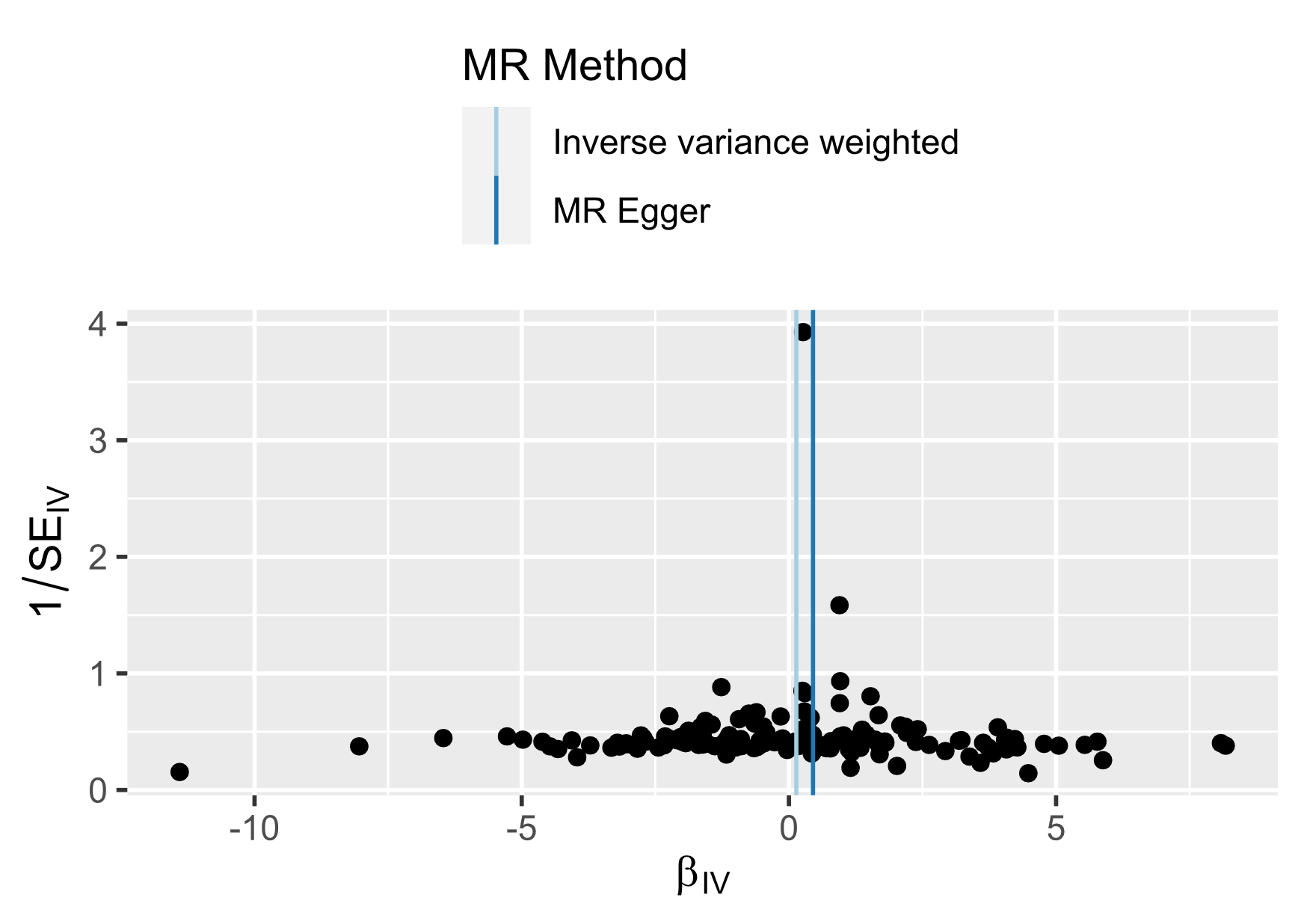


(E) Funnel plot of VitD for Diabetic neuropathy


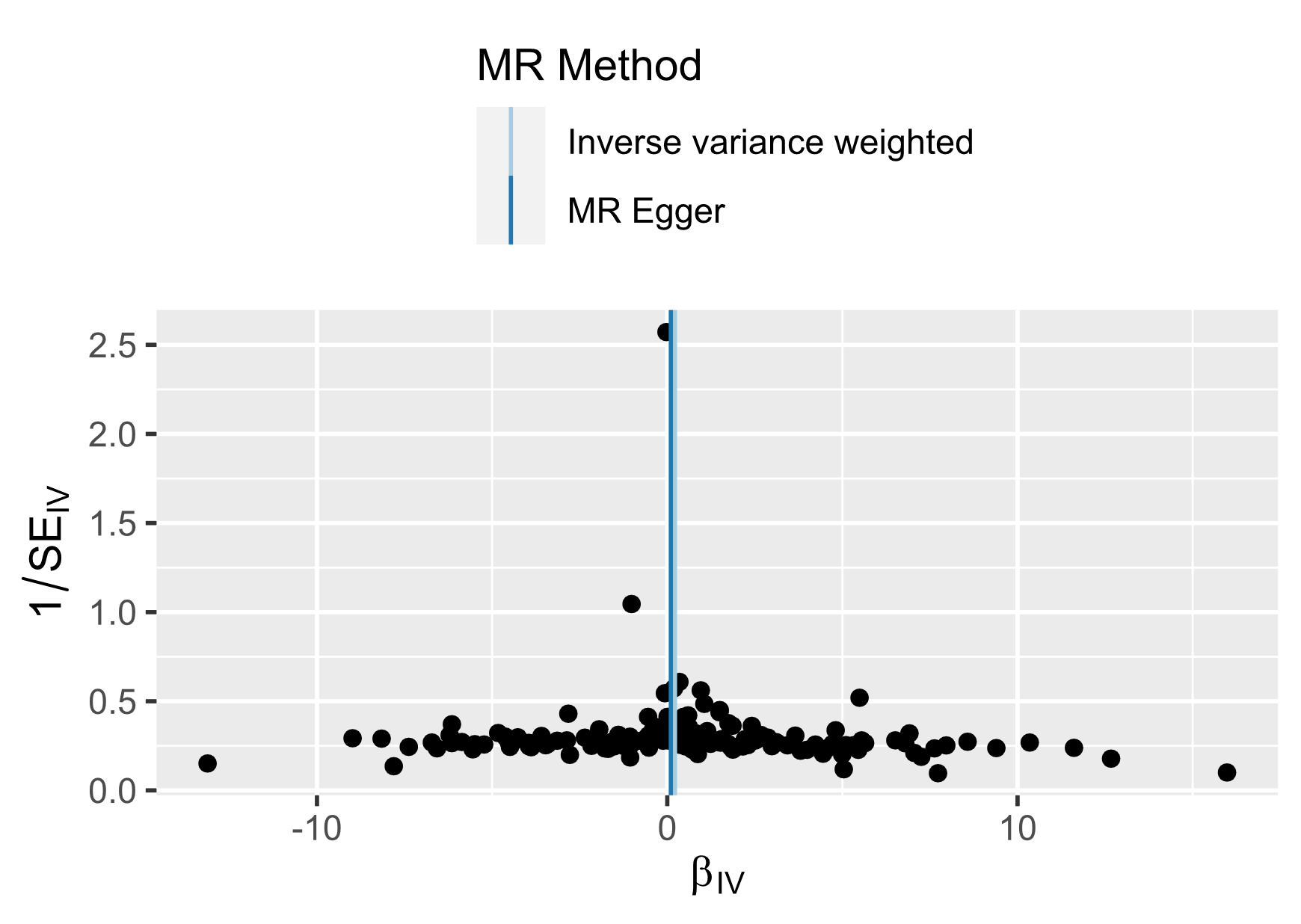


(F) Funnel plot of VitD for Diabetic retinopathy


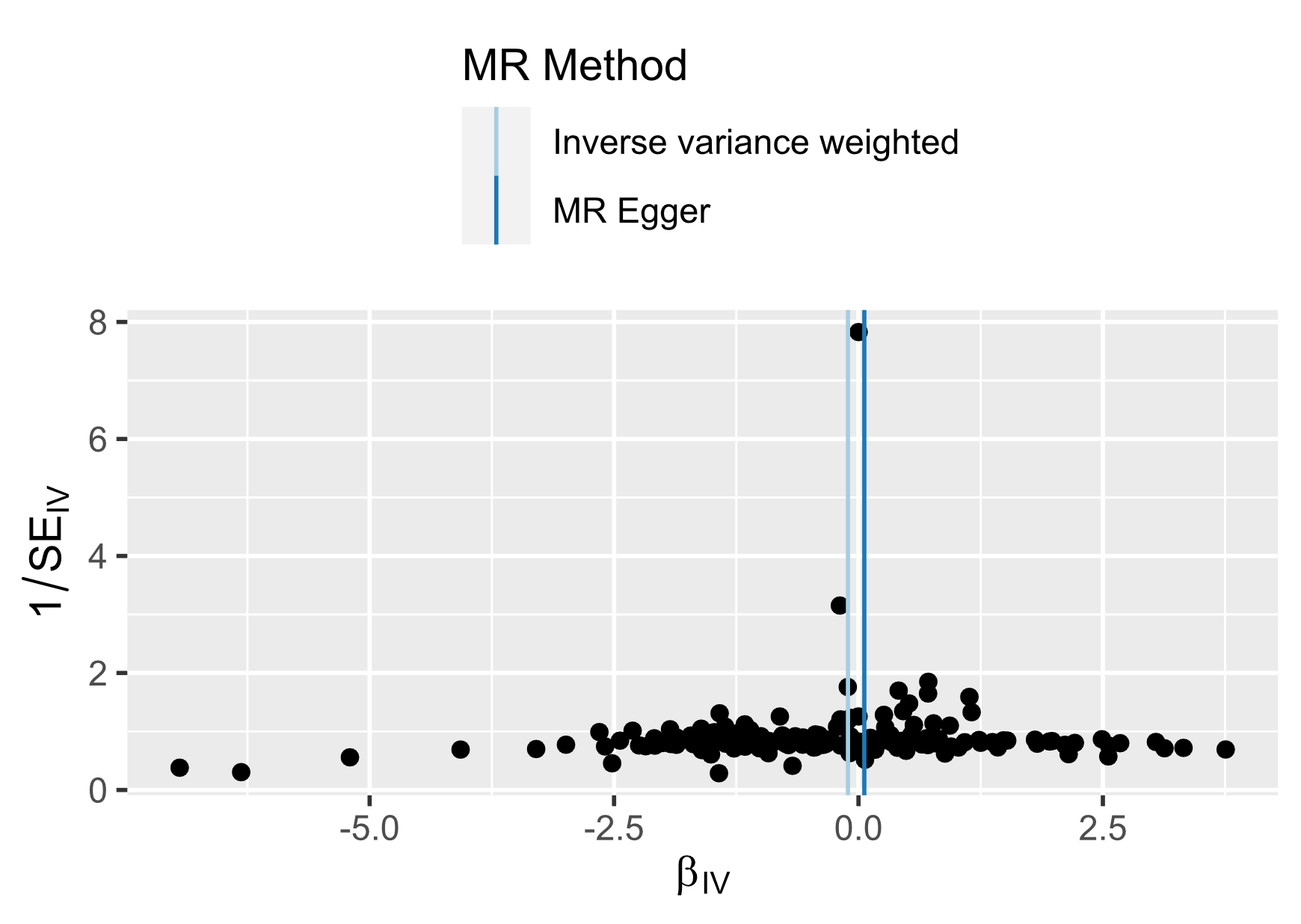


Supplementary material 7: Funnel plot of vitamin D for Diabetic complications, such as (A)Diabetic hypoglycemia, (B) Diabetic ketoacidosis, (C) Diabetic maculopathy, (D) Diabetic nephropathy, (E) Diabetic neuropathy and (F) Diabetic retinopathy.
