# Supplementary material for: Causal Relationship Between Various Vitamins and Different Diabetic Complications: A Mendelian Randomization Study
Source: Food Sci Nutr. 2025 Jul 7;13(7):e70536. doi: 10.1002/fsn3.70536 (PMC12230352; doi:10.1002/fsn3.70536)
Supplement: Supplementary file 9 — Appendix S9. Scatter plot of vitamin D for Diabetic complications, such as (A) Diabetic hypoglycemia, (B) Diabetic ketoacidosis, (C) Diabetic maculopathy, (D) Diabetic nephropathy, (E) Diabetic neuropathy and (F) Diabetic retinopathy. [file FSN3-13-e70536-s004.docx]

(A) Scatter plot of VitD on Diabetic hypoglycemia.


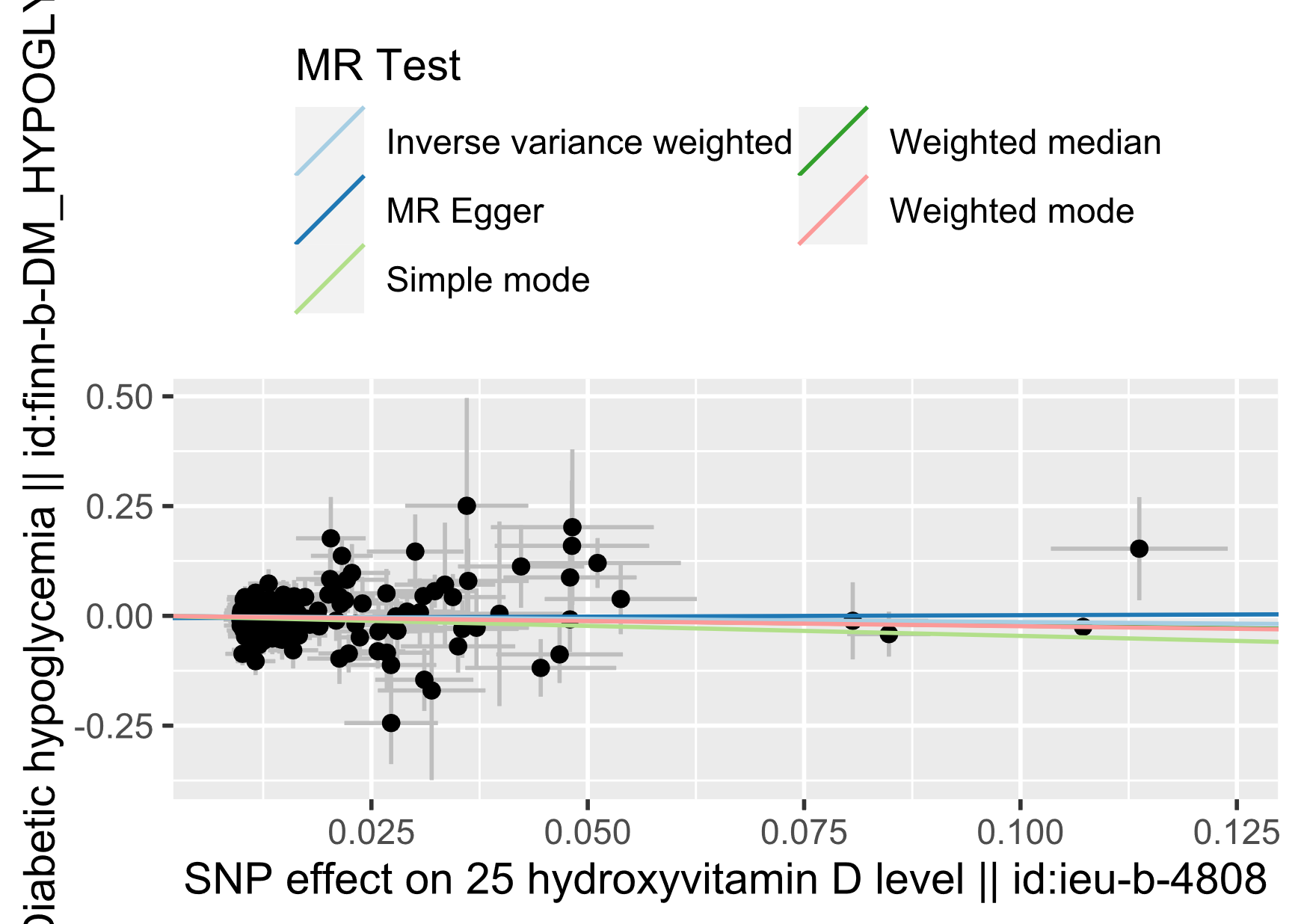


(B) Scatter plot of VitD on Diabetic ketoacidosis.


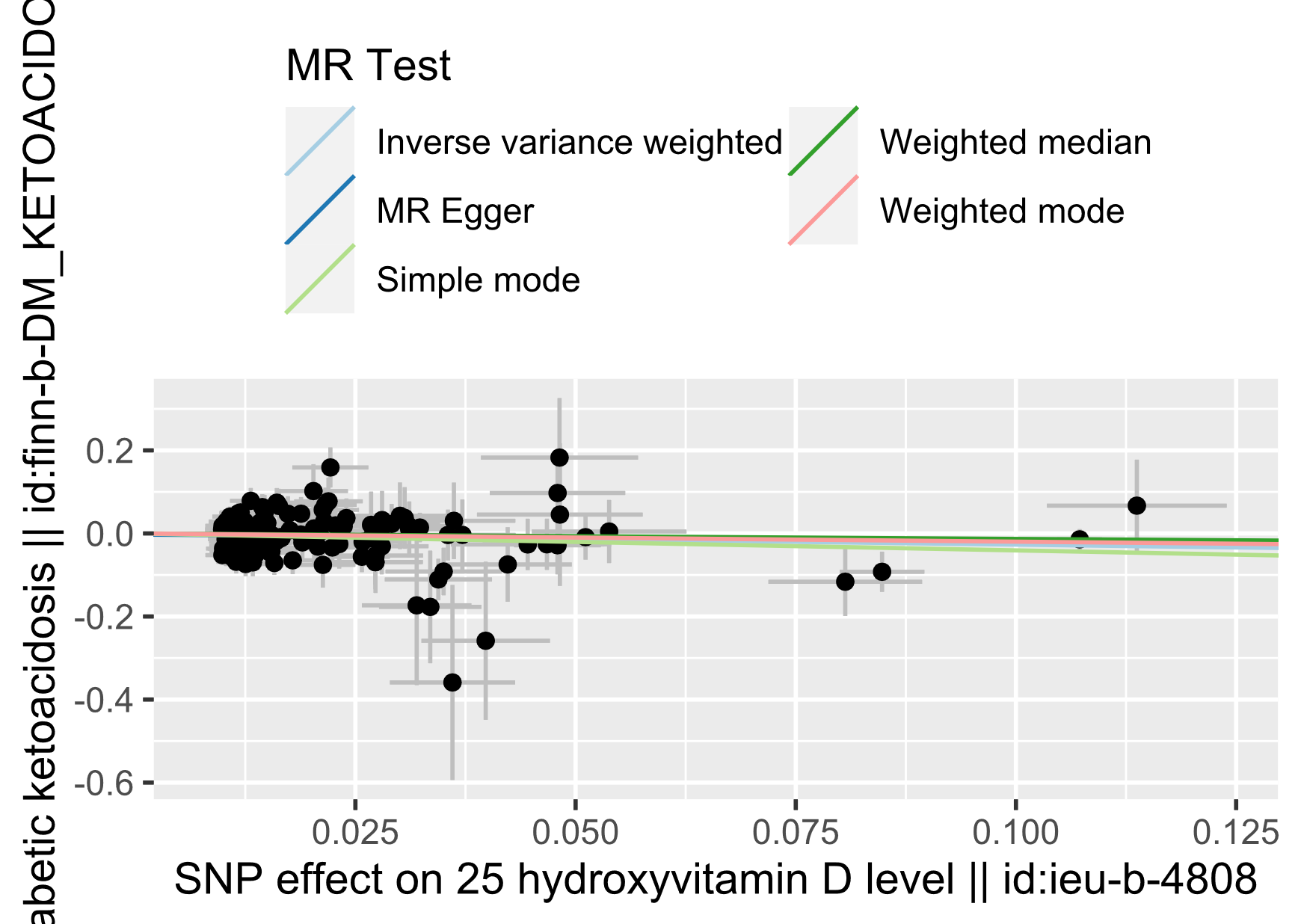


(C) Scatter plot of VitD on Diabetic maculopathy.


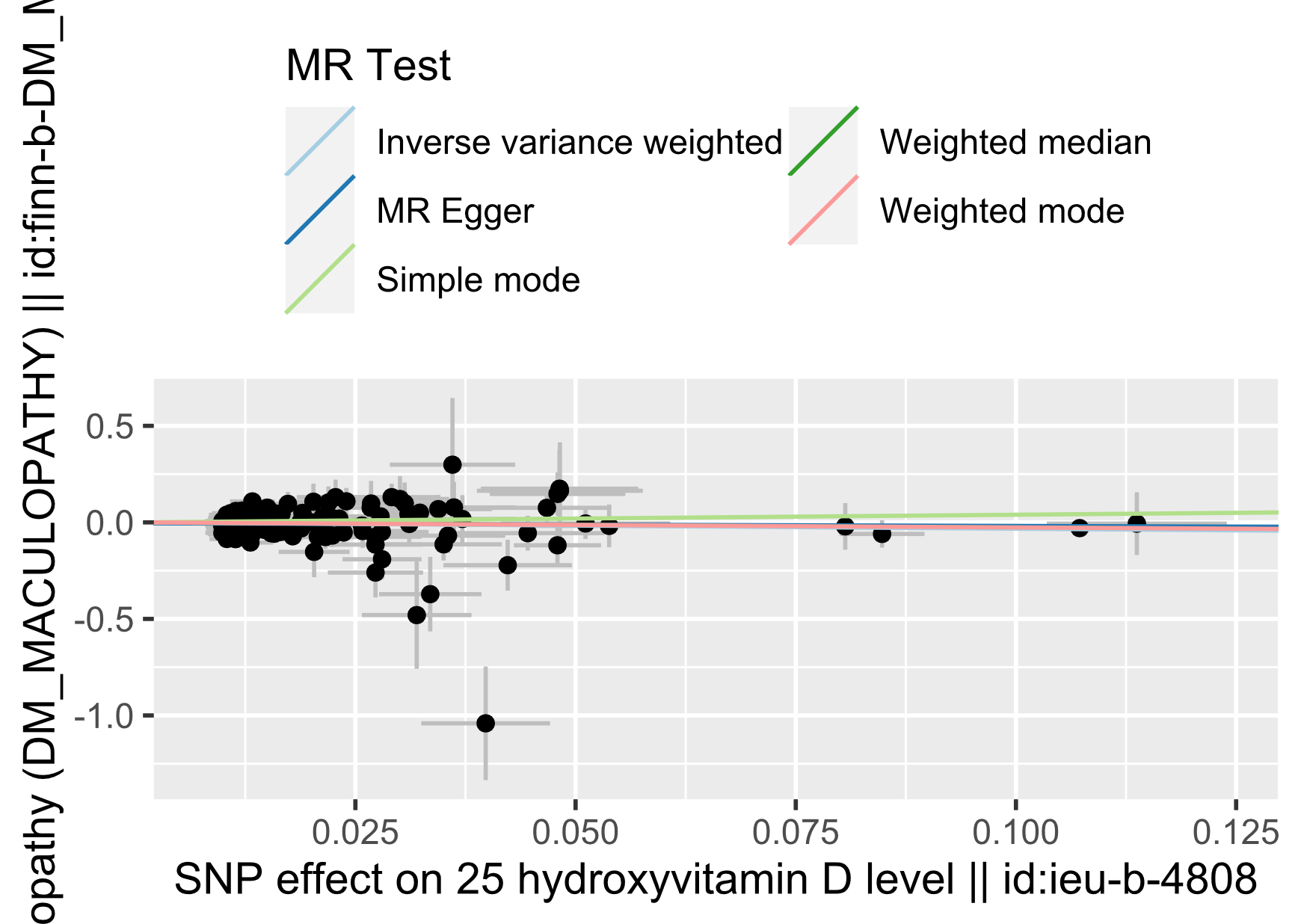


(D) Scatter plot of VitD on Diabetic nephropathy.


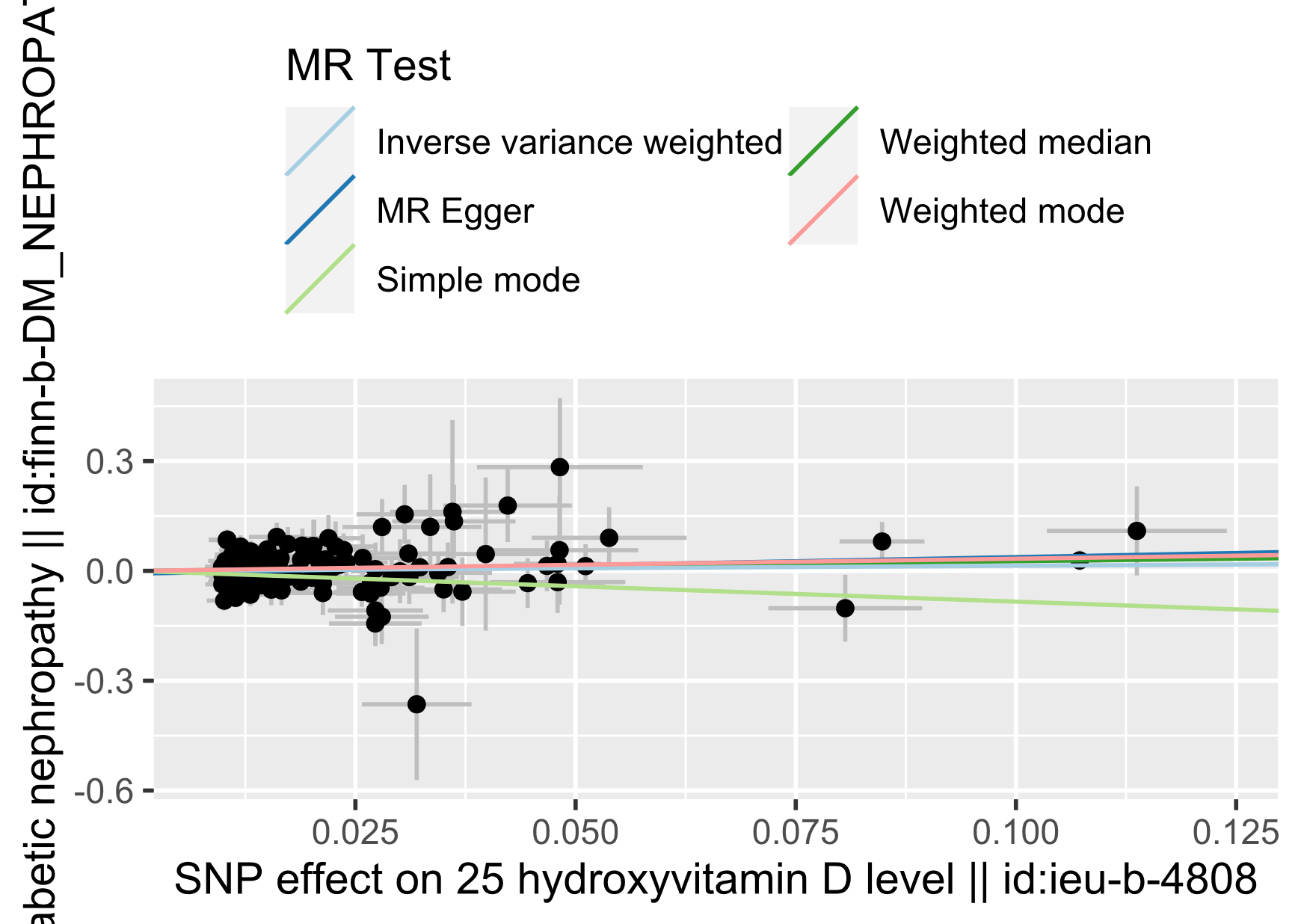


(E) Scatter plot of VitD on Diabetic neuropathy.


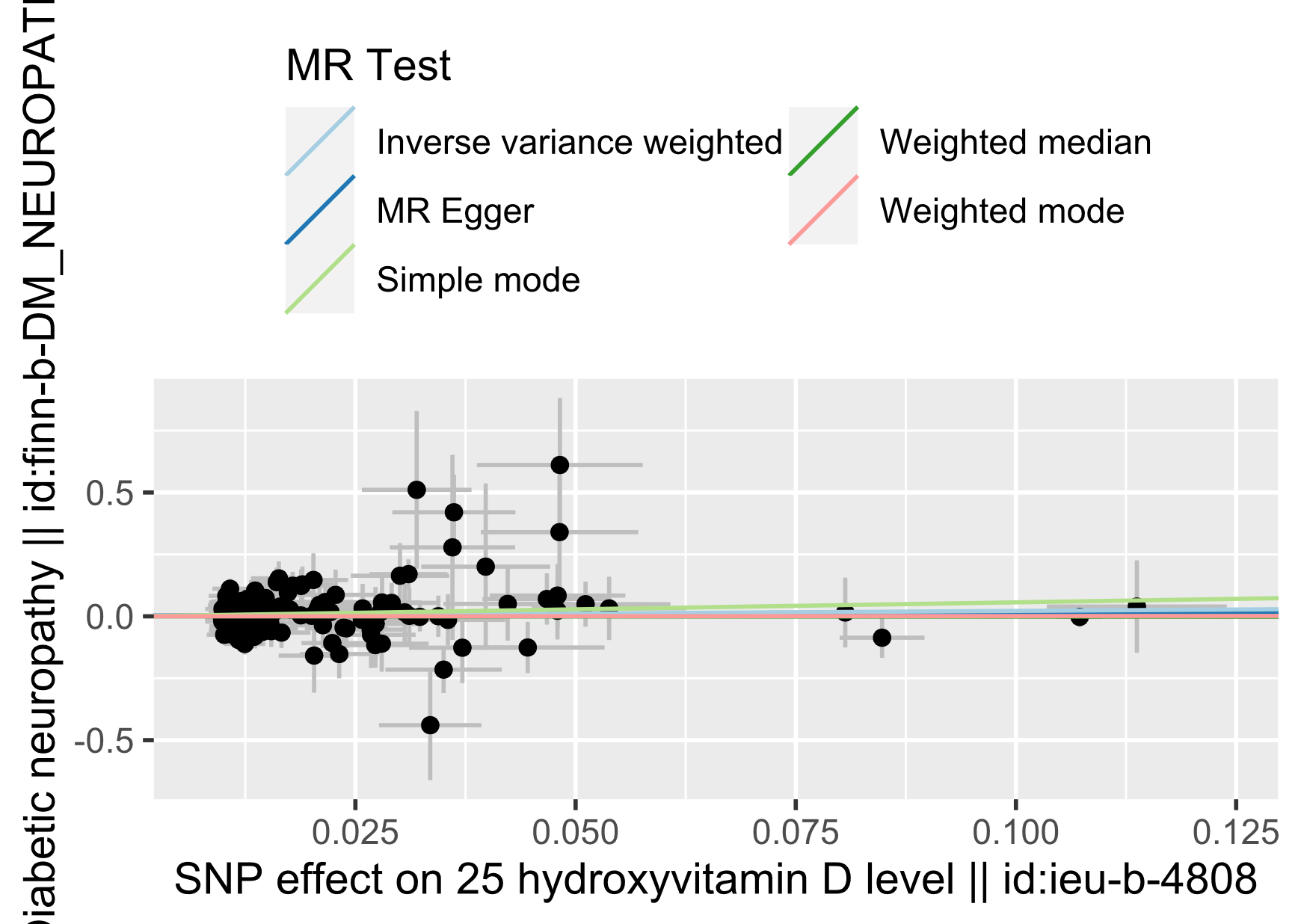


(F) Scatter plot of VitD on Diabetic retinopathy.


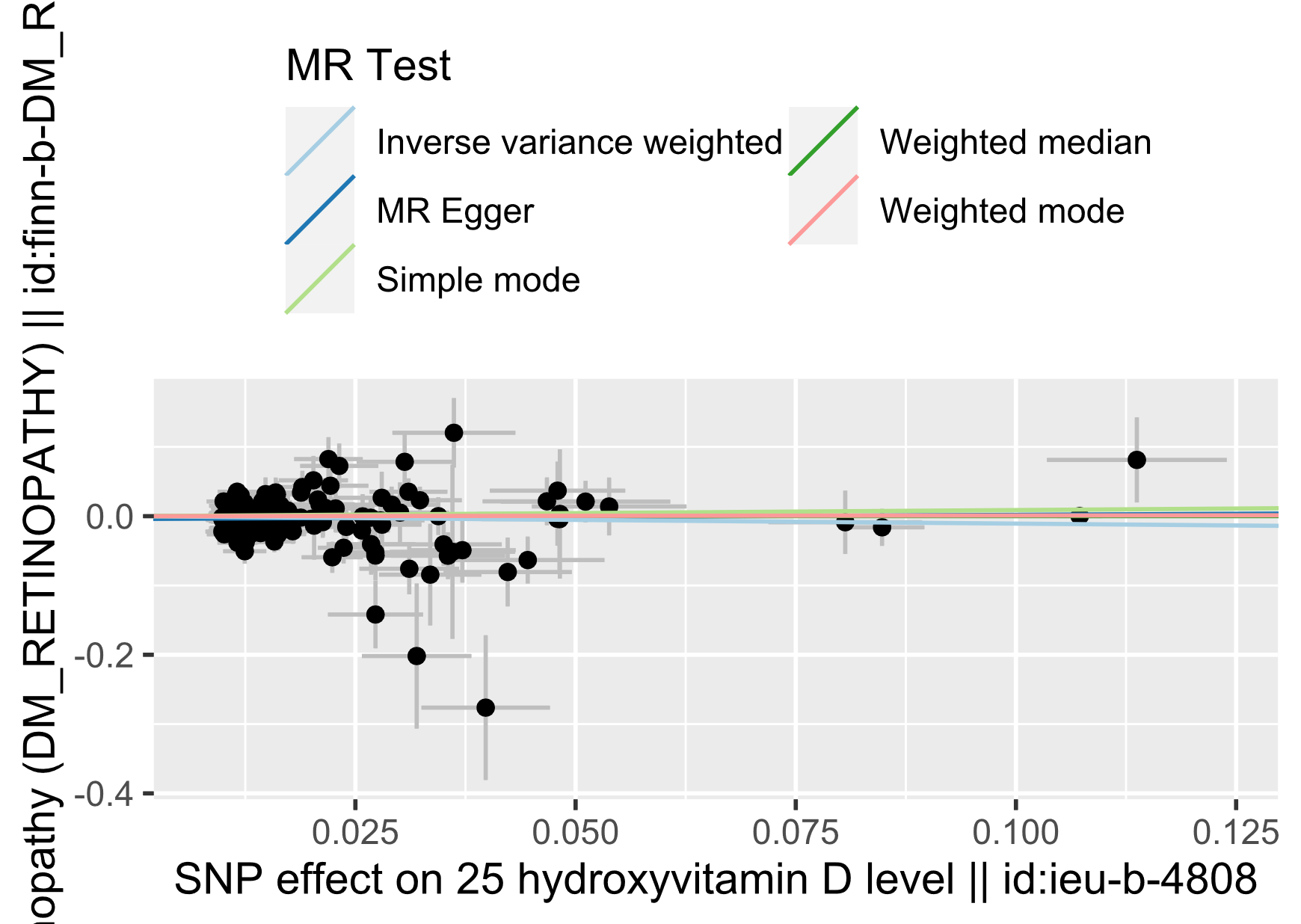


Supplementary material 9: Scatter plot of vitamin D for Diabetic complications, such as (A) Diabetic hypoglycemia, (B) Diabetic ketoacidosis, (C) Diabetic maculopathy, (D) Diabetic nephropathy, (E) Diabetic neuropathy and (F) Diabetic retinopathy.
