# Supplementary material for: Causal Relationship Between Various Vitamins and Different Diabetic Complications: A Mendelian Randomization Study
Source: Food Sci Nutr. 2025 Jul 7;13(7):e70536. doi: 10.1002/fsn3.70536 (PMC12230352; doi:10.1002/fsn3.70536)
Supplement: Supplementary file 11 — Appendix S11. Detailed results and sensitive analysis about reverse mendelian randomization of diabetic hypoglycemia and VitB6. [file FSN3-13-e70536-s005.docx]

**Table 1: Reverse Mendelian randomization of diabetic hypoglycemia and VitB_6_**

|  | Exposure | Outcome | Method | SNP | b | se | *P* | OR | LCI | UCI |
| --- | --- | --- | --- | --- | --- | --- | --- | --- | --- | --- |
| 1 | Diabetic hypoglycemia | VitB_6_ | MR Egger | 7 | 0.005542 | 0.016616 | 0.752248 | 1.005558 | 0.973336 | 1.038846 |
| 2 |  |  | Weighted median | 7 | 0.007331 | 0.008297 | 0.376925 | 1.007358 | 0.991109 | 1.023873 |
| 3 |  |  | Inverse variance weighted | 7 | 0.009695 | 0.009252 | 0.29472 | 1.009742 | 0.991596 | 1.02822 |
| 4 |  |  | Simple mode | 7 | -0.00747 | 0.014693 | 0.629514 | 0.992562 | 0.964387 | 1.021561 |
| 5 |  |  | Weighted mode | 7 | 0.005092 | 0.009392 | 0.607265 | 1.005105 | 0.986771 | 1.023779 |

SNP: Single nucleotide polymorphisms; LCI: 95% upper confidence interval; UCI: 95% Lower confidence interval; OR: Odds Ratio.

(A) Forest plot of hypoglycemia for VitB_6_


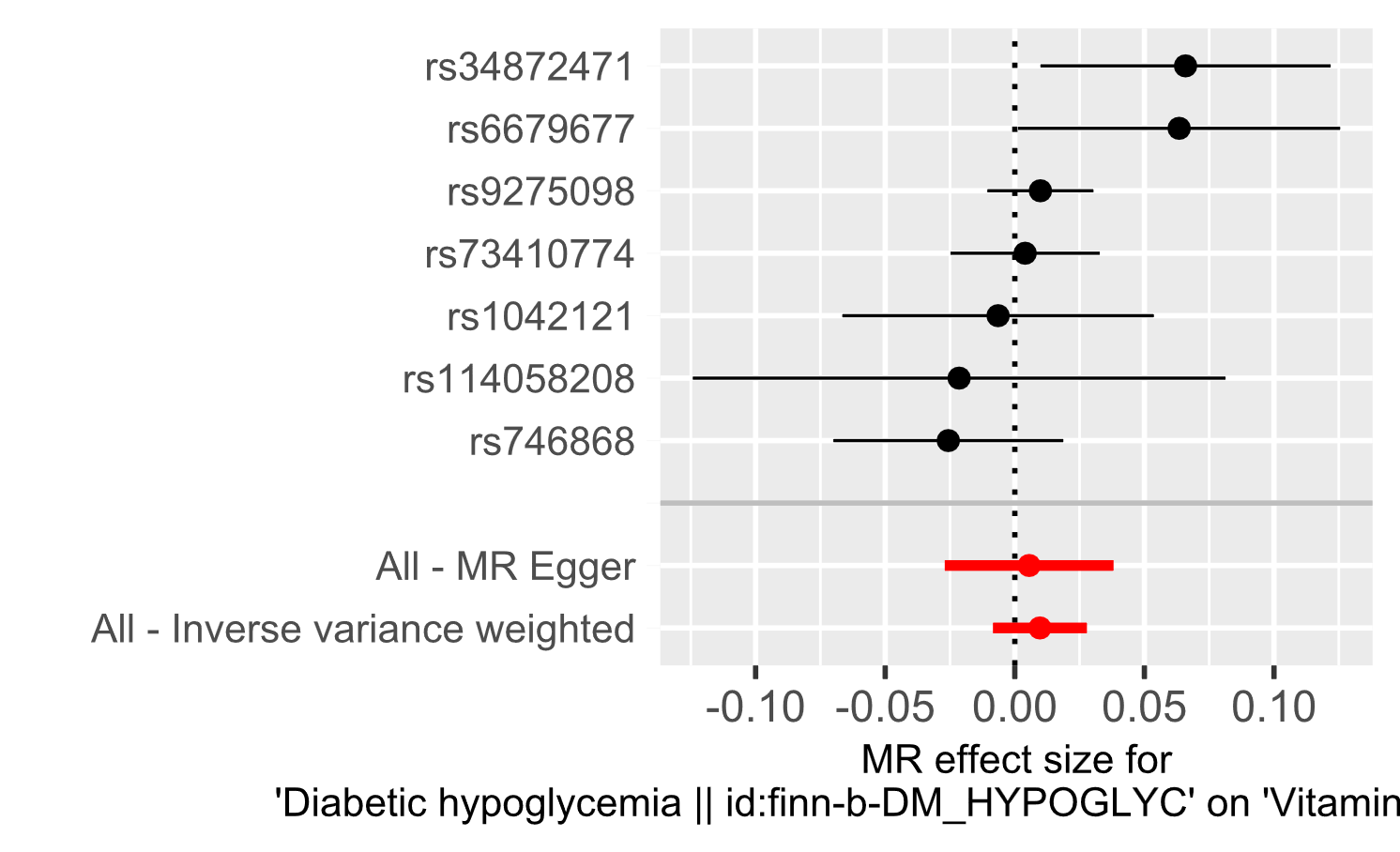


(B) Funnel plot of hypoglycemia for VitB_6_


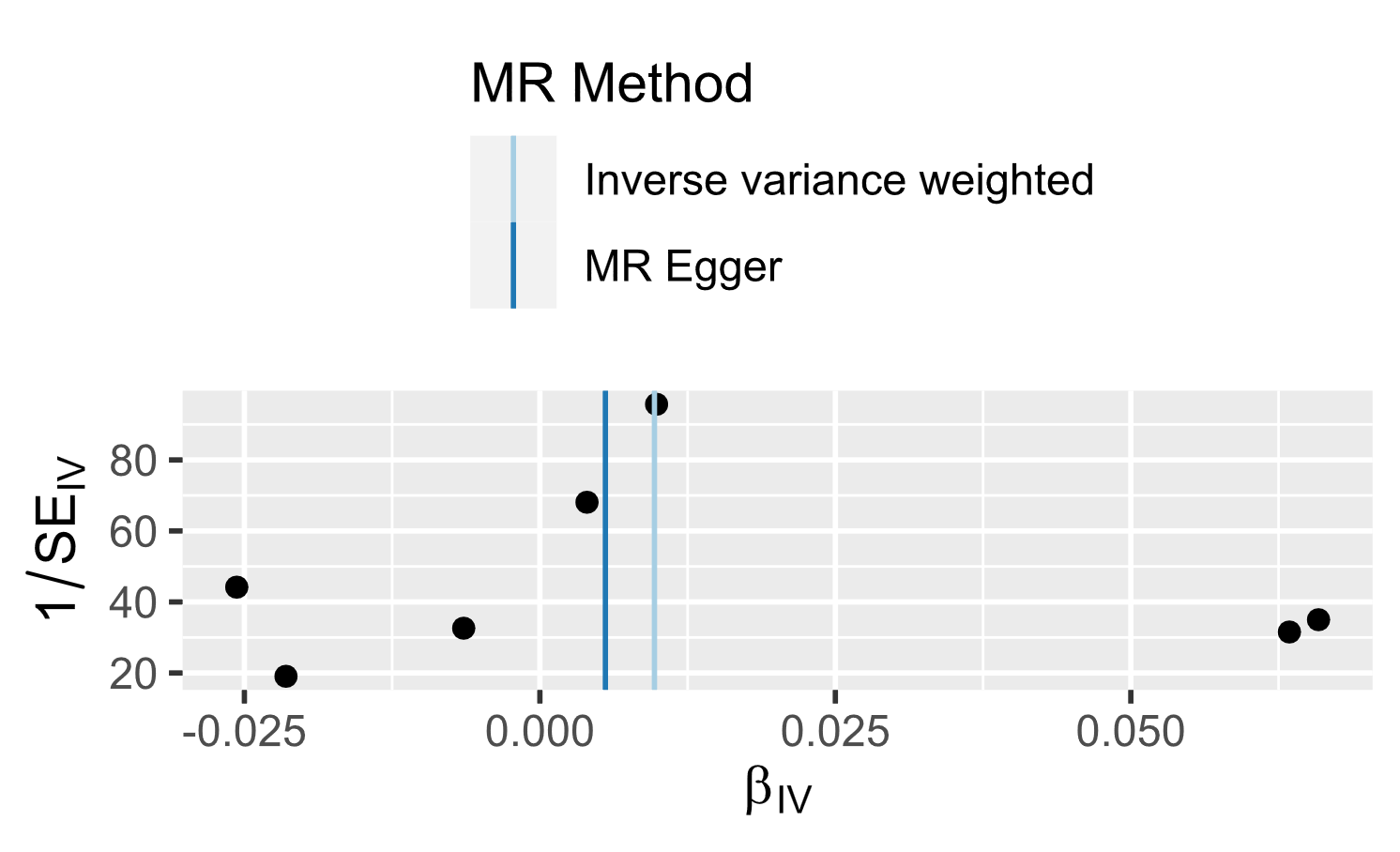


(C) Leave−one−out sensitivity analysis for hypoglycemia on VitB_6_


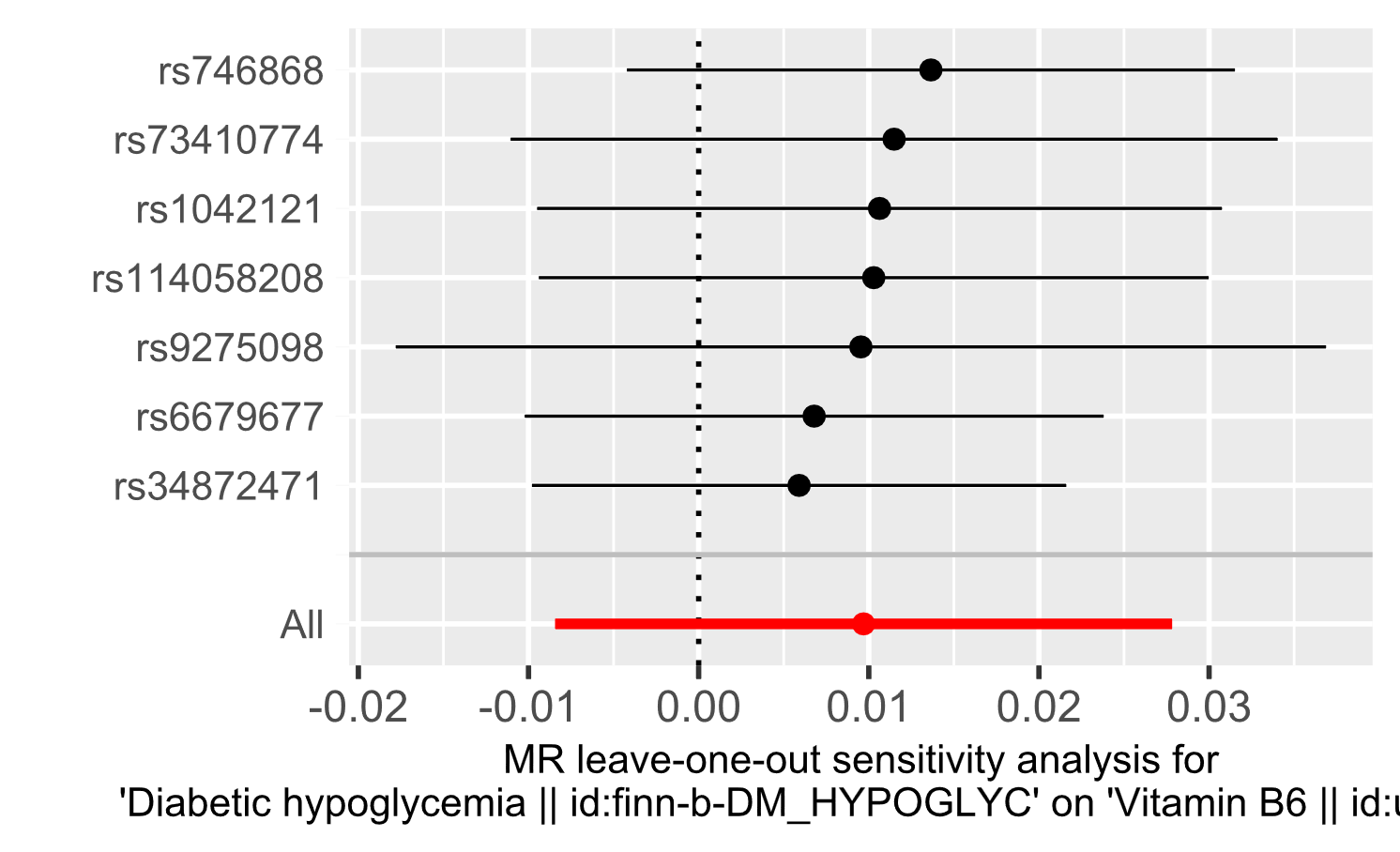


(D) Scatter plot of hypoglycemia on VitB_6._


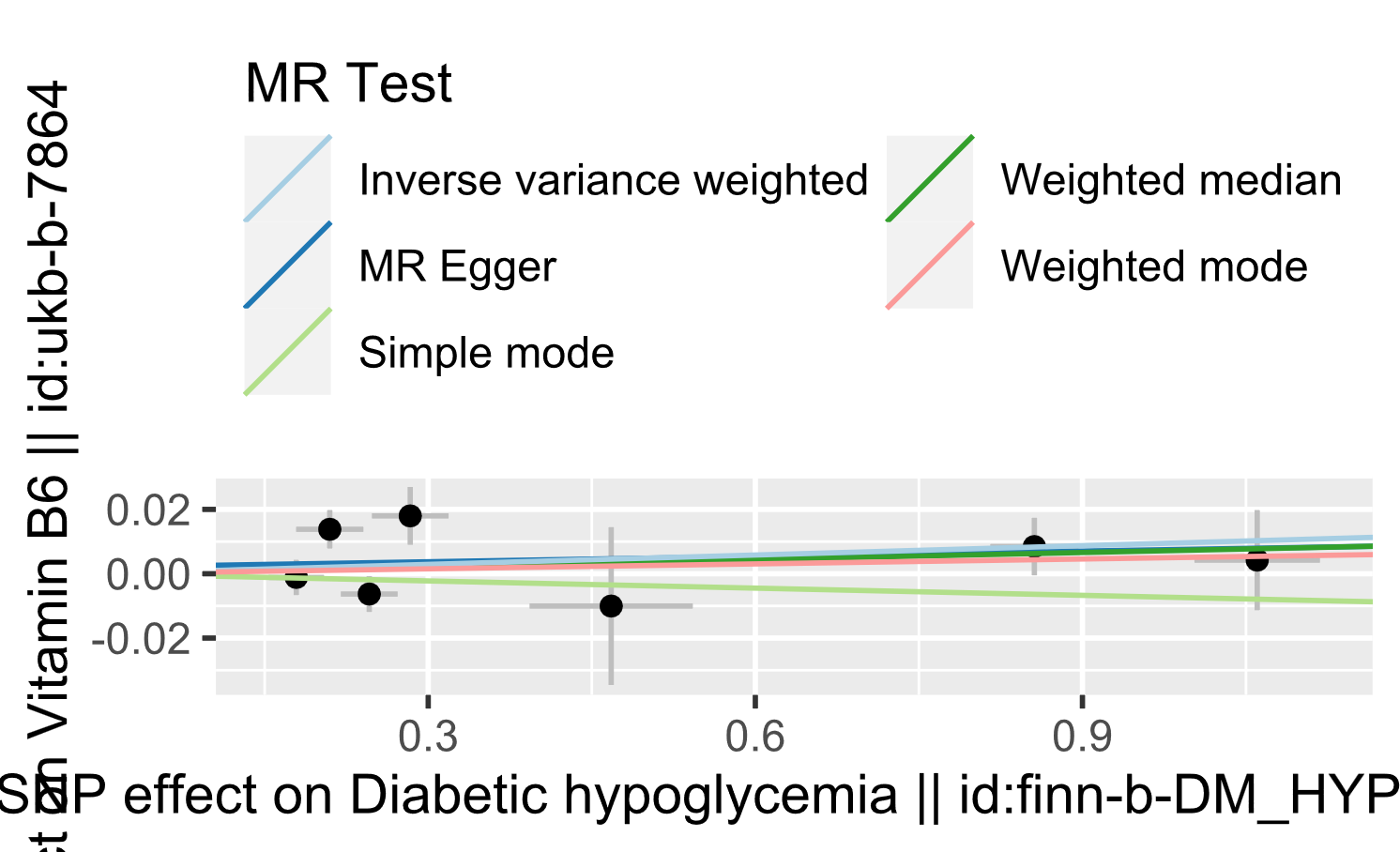


Figure 1: Detailed results and sensitive analysis about reverse mendelian randomization of diabetic hypoglycemia and VitB_6_, including: (A) Forest plot; (B) Funnel plot; (C) Leave−one−out sensitivity analysis and (D) Scatter plot.
